# Systematic Mining and Evaluation of the Sesquiterpene Skeletons as High Energy Aviation Fuel Molecules

**DOI:** 10.1002/advs.202300889

**Published:** 2023-06-04

**Authors:** Yanglei Huang, Ziling Ye, Xiukun Wan, Ge Yao, Jingyu Duan, Jiajia Liu, Mingdong Yao, Xiang Sun, Zixin Deng, Kun Shen, Hui Jiang, Tiangang Liu

**Affiliations:** ^1^ Key Laboratory of Combinatorial Biosynthesis and Drug Discovery Ministry of Education and School of Pharmaceutical Sciences Wuhan University Wuhan 430071 China; ^2^ State Key Laboratory of NBC Protection for Civilian Beijing 102205 China; ^3^ Frontier Science Center for Synthetic Biology and Key Laboratory of Systems Bioengineering (Ministry of Education) School of Chemical Engineering and Technology Tianjin University Tianjin 300072 China; ^4^ Frontier Technology Research Institute Tianjin University Tianjin 301700 China; ^5^ State Key Laboratory of Microbial Metabolism School of Life Sciences and Biotechnology Shanghai Jiao Tong University Shanghai 200030 China; ^6^ Hubei Engineering Laboratory for Synthetic Microbiology Wuhan Institute of Biotechnology Wuhan 430075 China

**Keywords:** aviation fuel, evaluation, mining, sesquiterpene

## Abstract

Sesquiterpenes have been identified as promising ingredients for aviation fuels due to their high energy density and combustion heat properties. Despite the characterization of numerous sesquiterpene structures, studies testing their performance properties and feasibility as fuels are scarce. In this study, 122 sesquiterpenoid skeleton compounds, obtained from existing literature reports, are tested using group contribution and gaussian quantum chemistry methods to assess their potential as high‐energy aviation fuels. Seventeen sesquiterpene compounds exhibit good predictive performance and nine compounds are further selected for overproduction in yeast. Through fed‐batch fermentation, all compounds achieve the highest reported titers to date. Subsequently, three representative products, pentalenene, presilphiperfol‐1‐ene, and *α*‐farnesene, are selected, produced, purified in large quantities, and tested for use as potential fuels. The performance of pentalenene, presilphiperfol‐1‐ene, and their derivatives reveals favorable prospects as high‐energy aviation fuels.

## Graphical abstract

A comprehensive database of 122 sesquiterpene molecules from plants, fungi, and bacteria, is established. The sesquiterpene compounds exhibit good predictive performance are selected and produced in yeast and tested performance compare with JP‐10 (high‐energy aviation fuel). The performance of pentalenene, presilphiperfol‐1‐ene, and their derivatives reveal favorable prospects as high‐energy aviation fuels.

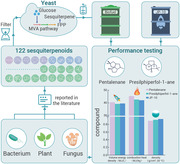

## 1 Introduction

The advancement of synthetic biology and metabolic engineering has opened up promising opportunities for the development of biofuels.^[^ ^1^ ^]^ Numerous biofuel molecules, such as short chain alcohols for gasoline,^[^ ^2^ ^]^ alkanes,^[^ ^3^ ^]^ fatty acid esters^[^ ^4^, ^5^, ^6^ ^]^ for alternative diesel, and isoprenoids^[^ ^7^ ^]^ for alternative aviation fuel, have been successfully biosynthesized.^[^ ^8^ ^]^ Despite the challenge of production costs,^[^ ^9^ ^]^ the structural diversity of advanced biofuels endows them with excellent properties as biofuels, indicating their potential to meet special application requirements.

Isoprenoids, particularly monoterpenes (C_10_H_16_) and sesquiterpenes (C_15_H_24_), have attracted significant attention as potential aviation fuels due to their low freezing points, high cetane numbers, and high energy densities.^[^ ^10^, ^11^, ^12^, ^13^, ^14^ ^]^ Among the various monoterpenes. Limonene, a common monoterpene widely used in food, pharmaceuticals, cosmetics, biomaterials, and biofuels. In recent studies, limonene has demonstrated excellent fuel properties.^[^ ^15^, ^16^, ^17^ ^]^ Similarly, linalool, a colorless liquid with a strong fragrance, has shown potential as a fuel due to its unique molecular structure.^[^ ^18^, ^19^, ^20^, ^21^ ^]^ Additionally, pinene's dimers exhibit comparable density and net heat of combustion to JP‐10.^[^ ^22^, ^23^ ^]^ Sesquiterpenes, like *β*‐farnesene, have promising fuel properties and the potential to be blended with other sustainable aviation fuel components to improve overall fuel performance.^[^ ^24^, ^25^ ^]^ Sesquiterpenes thujopsene, *α*‐cedrene, and *β*‐cedrene have a cetane number of 31 after hydrogenation and their volumetric net heat of combustion values are 12% higher than conventional biofuels.^[^ ^24^ ^]^ Furthermore, The combustion values of epi‐isozizaene, pentalenene, and *α*‐isocomene were predicted to be similar to Jet A‐1's value and researchers have produced all three compounds in *E. coli*. at milligram per liter levels.^[^ ^25^ ^]^

Monoterpenes have shown significant potential as aviation fuels. While sesquiterpenes, may play a different role in aviation fuels. Despite an increasing number of sesquiterpene discoveries, only a few have been tested as components of aviation fuels, and their feasibility analysis is still in the theoretical stage. This limitation is attributed to the low biosynthetic capacity of sesquiterpenes. Although farnesene has reached the stage of industrial production, its application is constrained by its high viscosity. However, it could still be used in jet fuel. There are still other potential sesquiterpenes that deserve to be discovered. A lack of studies that have been able to systematically predict, prepare through biosynthesis, and conduct practical property testing of potential sesquiterpene molecules, which hampers the application of sesquiterpenoids as aviation fuels.

In this study, we systematically describe the technological process for evaluating the performance of sesquiterpenes as potential biofuels. Through performance testing, pentalenene, and presilphiperfol‐1‐ene were identified as potential candidates for aviation fuel molecules. This study has significant implications for the exploration and industrial production of sesquiterpenes as aviation biofuels.

## 2 Results

### 2.1 Physicochemical and Energy Properties Calculation of Derivatives of Sesquiterpenoids

**Figure 1:**
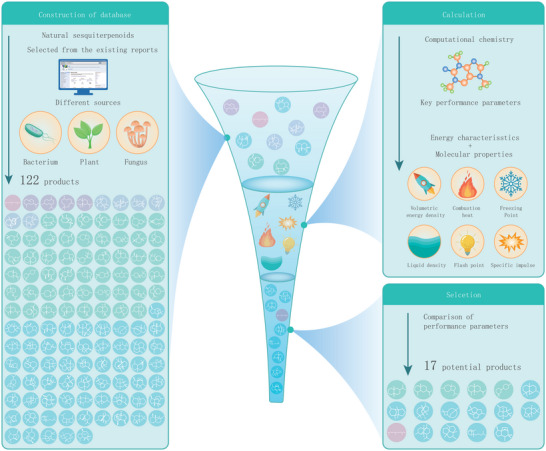
Overview of performance prediction of sesquiterpenoids from different sources. 122 compound molecules from different sources (plants, fungi, and bacteria) were selected from existing reports. The key fuel physical properties for the 122 hydrogenated products were calculated using computational chemistry methods. First, based on the QM9 database, a group contribution method of Marrero–Gani was designed to compute the molecular performance, including freezing point, liquid density, and flash point. Sequentially, the specific energy of molecules was computed according to the isodesmic reactions, including volumetric energy density, specific impulse, and combustion heat. Finally, the six key fuel physical properties for the 122 hydrogenated products were compared with JP‐10, which is high energy fuel currently used for military aviation fuel.

We collected the skeletal molecules of sesquiterpenoids reported in the literature and selected 122 representative molecules from various sources such as plants, fungi, and bacteria (**Figure** **1**). These skeletal molecules were categorized into acyclic (1), monocyclic (4), bicyclic (51), tricyclic (60), and tetracyclic (6) compounds based on their structural characteristics. The properties of the compounds after hydrogenation were calculated using the group contribution and gaussian quantum chemistry method.^[^ ^26^, ^27^ ^]^ These properties include net heats of combustion, volumetric energy density, freezing point, flash point, density, and specific impulse (Table S1, Supporting Information). Higher net heats of combustion indicate a greater amount of heat released per kilogram of the target molecule, while higher volumetric heat is advantageous for increasing the range for volume limited aircraft. Lower freezing points are preferred for use in low‐temperature, high‐altitude environments. Flash point is an important safety indicator for fuel storage, transportation, and use. Additionally, specific impulse represents the power performance of the fuel. The JP‐10 fuel is currently utilized for high energy military aviation and serves as a useful benchmark for assessing new fuel discoveries.^[^ ^22^, ^28^, ^29^, ^30^, ^31^ ^]^ The results obtained from the calculations revealed that the majority of the 122 hydrogenated sesquiterpenes demonstrated higher combustion heat compared to JP‐10. Furthermore, a number of sesquiterpenes exhibited better performance than JP‐10 in terms of density, flash point, freezing point, and specific impulse (Table S1, Supporting Information).

### 2.2 Rapid Characterization of Sesquiterpenoids Synthases

**Figure 2:**
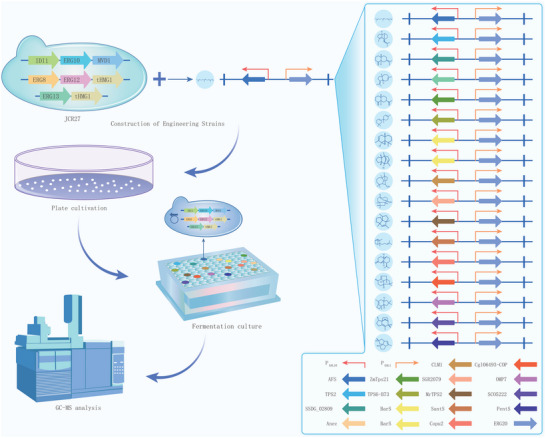
Characterization of sesquiterpenoids synthases. Flowchart of sesquiterpenoids synthases characterization in engineered yeast JCR27. The P*_GAL1_*‐P*_GAL10_* promoter was used to control the expression of the sesquiterpene synthase genes and farnesyl pyrophosphate synthase gene (*ERG20*); the relevant plasmids were expressed in the JCR27 strain to construct the engineering strains; then, the strains were fermentation in 96‐deep well microtiter plates, and the cultures were used to analyze the product by gas chromatography‐mass spectrometry (GC‐MS).

The results of the performance prediction revealed that sesquiterpenoids exhibit significant potential as raw materials for aviation fuels. Seventeen compounds, with well‐characterized biosynthetic genes and superior performance parameters, were selected (**Figure** **2**). These compounds include farnesene, the sole acyclic compound, several bicyclic compounds such as valerene (ranked first in volumetric energy), selina‐4,11‐diene (ranked fifth in volumetric energy), daucene (ranked seventh in volumetric energy), *β*‐selinene (ranked fifteenth in volumetric energy), and macrocarpene (ranked first in flash point). Furthermore, tricyclic compounds such as *α*‐barbatene (ranked second in volumetric energy), longibornene (ranked third in volumetric energy), caryolene (ranked sixth in volumetric energy), *α*‐isocomene (ranked seventh in volumetric energy), thujopsene (ranked fourteenth in volumetric energy), *α*‐santalene (ranked first in freezing point), *α*‐copaene (ranked 7th in freezing point), presilphiperfol‐1‐ene (ranked 24th in specific impulse), and Δ6‐protoilludene (ranked 27th in specific impulse) were also included in the selection. Additionally, epi‐isozizaene and pentalenene were included in the test compounds based on previous studies that identified them as potential candidates for aviation fuels^[^ ^25^ ^]^ (**Table** **1**; and Tables S2–S6, Supporting Information).

**Table 1:** Biosynthetic information of 17 target sesquiterpenoids

Structure	Compound^a)^	Performance	Rank	Sesquiterpene	Synthase	Plasmid	Strain	Product profile
acyclic	*α*‐Farnesene	Volumetric energy density	1 st	*α*‐Farnesene	AFS	pZY901	JTPS‐AFS	Single
bicyclic	Valerene	Volumetric energy density	1 st	Valerena‐4,7(11)‐diene	TPS2	pTPS‐C15‐69	JTPS‐C15‐69	Multiple products
	Selina‐4,11‐diene	Volumetric energy density	5 th	Selina‐4(15),7(11)‐diene	SSDG_02809	pTPS‐C15‐65	JTPS‐C15‐65	Multiple products
	Daucene	Volumetric energy density	7 th	dauca‐4,7‐diene	Anec	pTPS‐C15‐30	JTPS‐C15‐30	Multiple products
	*β*‐selinene	Volumetric energy density	15 th	*β*‐selinene	ZmTps21	pTPS‐C15‐61	JTPS‐C15‐61	Multiple products
	Macrocarpene	Flash point	1 st	(−) *β*‐Macrocarpene	TPS6‐B73	pTPS‐C15‐53	JTPS‐C15‐53	Multiple products
tricyclic	Thujopsene	Volumetric energy density	14th	Thujopsene	BarS	pTPS‐BarS	JTPS‐BarS	Double products
	*α*‐Barbatene	Volumetric energy density	2 nd	*α*‐Barbatene	BarS	pTPS‐BarS	JTPS‐BarS	Double products
	Longibornene	Volumetric energy density	3 rd	Longiborneol	CLM1	pTPS‐C15‐49	JTPS‐C15‐49	Multiple products
	Caryolene	Volumetric energy density	6 th	(+)‐Caryolan‐1‐ol	SGR2079	pTPS‐C15‐21	JTPS‐C15‐21	Single
	*α*‐isocomene	Volumetric energy density	7 th	*α*‐isocomene	MrTPS2	pTPS‐C15‐47	JTPS‐C15‐47	Multiple products
	*α*‐santalene	Freezing point	1 st	*α*‐santalene	SantS	pTPS‐SantS	JTPS‐SantS	Single
	*α*‐Copaene	Freezing point	7 th	*β*‐Copaene	Copu2	pTPS‐Copu2	JTPS‐Copu2	Single
	Presilphiperfol‐1‐ene	Specific impulse	24 th	Presilphiperfol‐1‐ene	Cgl06493‐COP	pXZ136	JTPS‐Cgl06493	Single
	Δ6‐Protoilludene	Specific impulse	27 th	Protoilludene	OMP7	pTPS‐Omp7	JTPS‐Omp7	Single
	epi‐isozizaene	Freezing point	14 th	epi‐isozizaene	SCO5222	pKZ762	JTPS‐SCO5222	Single
	Pentalenene	Freezing point	14 th	Pentalenene	PentS	pKZ761	JTPS‐pents‐sc	Single

^a)^ Compounds were ranked by the value of performance. For volumetric energy density, flash point and specific impulse, the larger the values, the higher the ranking of the compound. For freezing point, the smaller the value, the higher the ranking of the compound.

In recent research, there has been a growing interest in utilizing microorganisms for the synthesis of terpenoids. Particularly, isoprene, has been found to be direct produced from carbon dioxide in cyanobacteria.^[^ ^32^, ^33^ ^]^ Based on the requirements of our study, we have selected *Saccharomyces cerevisiae* as the host organism for biosynthesis. *Saccharomyces cerevisiae*, known for its safety, growth dominance, and mature genetic manipulation capabilities, has been widely regarded as the ideal microbial cell factory. In *S. cerevisiae*, sesquiterpenoids are synthesized from the precursor farnesyl pyrophosphate, which is derived from the mevalonate (MVA) pathway. However, the weak flux of the MVA pathway often limits the production of sesquiterpenoids.^[^ ^34^ ^]^ To overcome this limitation, the JCR27 strain,^[^ ^35^, ^36^, ^37^ ^]^ which has an enhanced MVA pathway, was chosen for efficient microbial synthesis of sesquiterpenes. Among the 17 compounds tested in study, the synthases corresponding to 16 compounds have been reported previously (Table 1). Interestingly, the synthase corresponding to presilphiperfol‐1‐ene was discovered during gene mining. The genes were codon‐optimized for *S. cerevisiae*, and the sesquiterpene synthase and FPP synthase genes (*ERG20*) were expressed under the control of galactose‐inducible promoters P*_GAL1_*‐P*_GAL10_* (plasmids information listed in Table 1). These plasmids were introduced into the JCR27 strain to generate mutant strains, including JTPS‐BarS, JTPS‐SCO5222, JTPS‐pents‐sc, JTPS‐Cgl06493, JTPS‐Copu2, JTPS‐AFS, JTPS‐SantS, JTPS‐Omp7, JTPS‐C15‐69, JTPS‐C15‐21, JTPS‐C15‐49, JTPS‐C15‐53, JTPS‐C15‐65, JTPS‐C15‐61, JTPS‐C15‐30, and JTPS‐C15‐47. The 16 mutant strains were fermented with YPDHG medium in 96‐well plates, followed by extraction of the products from the yeast cells with isopropyl myristate (IPM). The organic phase was isolated at the end of fermentation and analyzed using gas chromatography‐mass spectrometry (GC‐MS). The results revealed that strains JTPS‐SCO5222, JTPS‐pents‐sc, JTPS‐Cgl06493, JTPS‐Copu2, JTPS‐AFS, JTPS‐SantS, and JTPS‐Omp7 produced single products: epi‐isozizaene, pentalenene, presilphiperfol‐1‐ene, *β*‐copaene, *α*‐farnesene, *α*‐santalene, and protoilludene, with high intensity in GC‐MS (Figures S3–S9, Supporting Information). Strain JTPS‐C15‐21 produced caryolan‐1‐ol as the main product, but with low intensity in GC‐MS (Figure S10, Supporting Information). On the other hand, strains JTPS‐C15‐69 (Figure S11, Supporting Information), JTPS‐C15‐49 (Figure S12, Supporting Information), JTPS‐C15‐53 (Figure S13, Supporting Information), JTPS‐C15‐65 (Figure S14, Supporting Information), JTPS‐C15‐61 (Figure S15, Supporting Information), JTPS‐C15‐30 (Figure S16, Supporting Information), and JTPS‐C15‐47 (Figure S17, Supporting Information) produced valerena‐4,7(11)‐diene, longiborneol, *β*‐macrocarpene, selina‐4(15),7(11)‐diene, *β*‐selinene, dauca‐4,7‐diene, and *α*‐isocomene, respectively, as the main products along with byproducts. Strain JTPS‐BarS (Figure S2, Supporting Information) can produce *α*‐barbatene and thujopsene simultaneously in similar proportion.

### 2.3 Efficient Synthesis of Nine Target Sesquiterpenes in *Saccharomyces cerevisiae*

The efficient and singular production of seven compounds, *α*‐farnesene, pentalenene, presilphiperfol‐1‐ene, epi‐isozizaene, protoilludene, *α*‐santalene, and *β*‐copaene, can be achieved through fermentation. Additionally, two compounds, thujopsene and *α*‐barbatene, with potential as biofuels, were synthesized together by one strain. These nine compounds were selected as the target products for achieving efficient synthesis in *S. cerevisiae* through further metabolic engineering.

**Figure 3:**
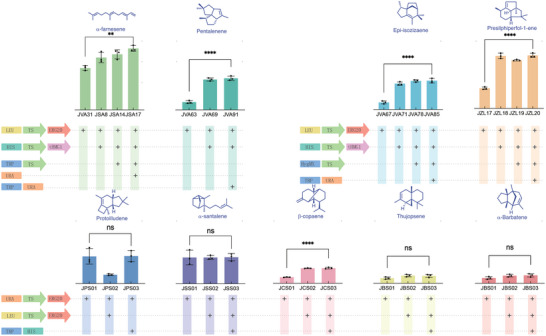
Sesquiterpenes production in shake flask fermentation. Gene in blue arrow (*tHMG1*), Gene in red arrow (*ERG20*), Gene in yellow arrow (terpenoid synthase, TS), rectangles (selection markers *LEU*, *HIS*, *URA*, and *TRP*). Bottom table indicates presence (+) of a specific gene in the listed strain. The strains were used for fermentation in shake flask at 72 h. Error bars indicate ± standard deviation (*n* = 3). Statistical differences were analyzed using the one‐way ANOVA (two‐tailed, two‐sample equal variance, **** represents *p*‐value <0.0001, ** represents *p*‐value <0.01, * represents *p*‐value <0.05, ns represents no significant difference).

In industrial fermentation, the gene expression of chromosomal integration is better than that of an episomal plasmid.^[^ ^38^ ^]^ Therefore, linearized plasmids pZY901, pKZ761, pKZ762, pXZ136, pTPS‐Omp7, pTPS‐SantS, pTPS‐Copu2, and pTPS‐BarS were integrated into the chromosome of strain JCR27, resulting in the generation of strains JVA31, JVA63, JVA67, JZL17, JPS01, JSS01, JCS01, and JBS01 (**Figure** **3**). After shake‐flask fermentation for 72 h, strain JVA31 produced 406 mg L^−1^ of *α*‐farnesene, strain JVA63 produced 76 mg L^−1^ of pentalenene, strain JVA67 produced 70 mg L^−1^ of epi‐isozizaene, strain JZL17 produced 196 mg L^−1^ of presilphiperfol‐1‐ene, strain JPS01 produced 263 mg L^−1^ of protoilludene, strain JSS01 produced 254 mg L^−1^ of *α*‐santalene, strain JCS01 produced 60 mg L^−1^ of *β*‐copaene, and strain JBS01 produced 51 mg L^−1^ of thujopsene and 53 mg L^−1^ of *α*‐barbatene. Further optimization of the sesquiterpene synthesis pathway can be achieved by adjusting the copy number of rate‐limiting enzymes such as *tHMG1*, *ERG20*, and sesquiterpene synthase, which may lead to improved titers of the target products.^[^ ^39^, ^40^ ^]^

After optimizing the metabolic pathway, high titer strains for nine compounds were achieved. After 72 h shake‐flask fermentations, the titer of strains exhibiting optimal performance was determined (Figure 3 and **Table** **2**). Strain JSA17, derived from JCR27 and engineered with an additional copy of *ERG20*, *tHMG1*, and three copies of *α*‐farnesene synthase, produced 595 mg L^−1^ of *α*‐farnesene. Strain JVA91, derived from JCR27 and engineered with an additional copy of *ERG20*, *tHMG1*, and two copies of pentalenene synthase, produced 306 mg L^−1^ of pentalenene. Strain JVA85, derived from JCR27 and engineered with an additional copy of *ERG20, tHMG1*, and three copies of epi‐isozizaene synthase, produced 280 mg L^−1^ of epi‐isozizaene. Strain JZL20, derived from JCR27 and engineered with an additional copy of *ERG20*, *tHMG1*, and three copies of presilphiperfol‐1‐ene synthase, produced 515 mg L^−1^ of presilphiperfol‐1‐ene. Strain JPS03, derived from JCR27 and engineered with an additional copy of *ERG20* and one copy of protoilludene synthase, produced 266 mg L^−1^ of protoilludene. Strain JSS03, derived from JCR27 and engineered with two copies of *ERG20* and two copies of *α*‐santalene synthase, produced 257 mg L^−1^ of *α*‐santalene. Strain JCS03, derived from JCR27 and engineered with two copies of *ERG20* and two copies of *β*‐copaene synthase, produced 148 mg L^−1^ of *β*‐copaene. Finally, strain JBS03, derived from JCR27 and engineered with two copies of *ERG20* and two copies of thujopsene and *α*‐barbatene synthase, produced 73 mg L^−1^ of thujopsene and 78 mg L^−1^ of *α*‐barbatene. Notably, to the best of our knowledge, this is the first reported synthesis of protoilludene, presilphiperfol‐1‐ene, and *β*‐copaene in *S. cerevisiae*.

**Table 2:** The titers of 9 sesquiterpenes in this study

Sesquiterpene	Strain	Titer (shake‐flask fermentation) [mg L^−1^]	Titer (Fed‐batch fermentation) [g L^−1^]
*α*‐Farnesene	JSA17	595	38.8
Pentalenene	JVA91	306	10.8
epi‐isozizaene	JVA85	280	4.7
Presilphiperfol‐1‐ene	JZL20	515	22.7
Protoilludene	JPS03	266	12.1
*α*‐santalene	JSS03	257	10.2
*β*‐Copaene	JCS03	148	6.8
Thujopsene	JBS03	73	1.2
*α*‐Barbatene	JBS03	78	1.6

### 2.4 Fed‐Batch Fermentation and Purification of Sesquiterpenes

**Figure 4:**
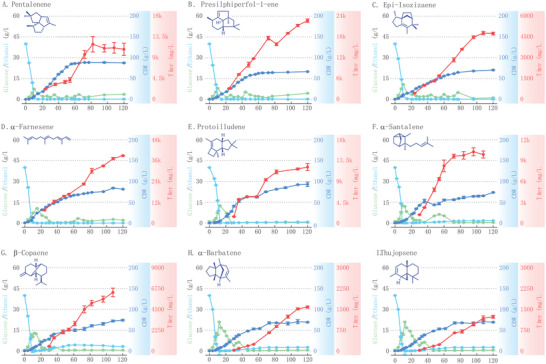
High‐density fed‐batch fermentation for sesquiterpene production in the engineered *S. cerevisiae*. Time courses showing changes in cell dry weight (CDW), glucose consumption, ethanol concentration, and sesquiterpene production. A) Pentalenene production in the strain JVA 91. B) Presilphiperfol‐1‐ene production in the strain JZL 20. C) Epi‐isozizaene production in the strain JVA 85. D) *α*‐Farnesene production in the strain JSA 17. E) Protoilludene production in the strain JPS 03. F) *α*‐Santalene production in the strain JSS 03. G) *β*‐Copaene production in the strain JCS 03. H) *α*‐Barbatene production in the strain JBS 03. I) Thujopsene production in the strain JBS 03. Titers of compound were measured by GC–MS at multiple time points. Data represent averages of three biological replicates with standard deviation as error bars.

In order to evaluate the suitability of various strains for industrial production and to obtain sufficient quantities of target products for performance assessment, we employed the following strains: JVA91 (pentalenene), JZL20 (presilphiperfol‐1‐ene), JVA85 (epi‐isozizaene), JSA17 (*α*‐farnesene), JPS03 (protoilludene), JSS03 (*α*‐santalene), JCS03 (*β*‐copaene), and JBS03 (*α*‐barbatene and thujopsene) for fed‐batch fermentation in a 15 L bioreactor. The fermentation process consisted of two stages. The first stage is a growth stage; the initial glucose concentration in the medium was 40 g L^−1^. Once it dropped to around 1 g L^−1^, the glucose concentration was controlled at 1–2 g L^−1^ to ensure growth of the strains by adjusting the glucose feed rate. The second stage was the product accumulation stage, where IPM was added to the medium at 24 h, and the ethanol concentration was controlled at 0–5 g L^−1^ to facilitate gene expression and product accumulation by adjusting the sucrose feed rate. Fermentation was terminated when production accumulation ceased to increase. This method was proven to enhance the titer due to improved strain growth and appropriate feeding strategies.^[^ ^37^ ^]^ Ultimately, we achieved fermentation titers of 10.8 g L^−1^ for pentalenene, 22.7 g L^−1^ for presilphiperfol‐1‐ene, 4.7 g L^−1^ for epi‐isozizaene, and 38.8 g L^−1^ for *α*‐farnesene, 12.1 g L^−1^ for protoilludene, 10.2 g L^−1^ for *α*‐santalene, 6.8 g L^−1^ for *β*‐copaene, 1.6 g L^−1^ for *α*‐barbatene, and 1.2 g L^−1^ for thujopsene (**Figure** **4** and Table 2). These titers represent the highest reported levels for microbial heterologous synthesis to date.

After the completion of fermentation, the organic phase containing the desired target products was separated from the fermentation broth. It was anticipated that the boiling points of the products would be lower than 260 °C at 760 mmHg, whereas the boiling point of IPM is 320 °C at 760 mmHg. To achieve purification, vacuum distillation was employed. However, the purified products of protoilludene, *α*‐santalene, *β*‐copaene, *α*‐barbatene, thujopsene, and epi‐isozizaene were insufficient to meet the demand for subsequent performance testing. In future studies, optimization of the biosynthetic pathway could be explored to enhance the titer of the target compounds. This could involve optimizing cofactor supply, reducing competing pathways, and improving precursor supply based on previous studies.^[^ ^41^, ^42^, ^43^ ^]^

On the other hand, pentalenene, presilphiperfol‐1‐ene (solid), and *α*‐farnesene were found to meet the requirements as they exhibited high purity (exceeding 95%) and were available in large quantities, making them suitable for further performance testing.

### 2.5 Performance Testing of Hydrogenated Products from Bio‐Fermented Sesquiterpenes

In order to assess their viability as potential aviation fuel precursors, pentalenene, presilphiperfol‐1‐ene, and *α*‐farnesene were subjected to hydrogenation using a Pd/C catalyst. Intriguingly, pentalenene and *α*‐farnesene remained colorless and transparent liquids both before and after hydrogenation, whereas presilphiperfol‐1‐ene, originally in solid form at room temperature, transformed into a liquid after hydrogenation. The three hydrogenated products were then tested for volumetric energy density, net heats of combustion, density, and freezing point (**Table** **3**). Deviation of the measured values for farnesane, presilphiperfol‐1‐ane, and pentalenane from the predicted values (except for freezing point) was less than 7%, indicating overall consistency with the predicted results. Specifically, the net heats of combustion for pentalenane, presilphiperfol‐1‐ane, and farnesane were measured at 45.4, 42.7, and 44.08 MJ kg^−1^, respectively, representing increases of 8%, 1%, and 5% compared to JP‐10. The densities of pentalenane, presilphiperfol‐1‐ane, and farnesane were found to be 0.8932, 0.919, and 0.7698 g mL^−1^ (at 20 °C), respectively, which were 5%, 2%, and 18% lower than the density of JP‐10. Notably, farnesane exhibited the lowest density, resulting in a lower volume energy density of 33.93 MJ L^−1^, 14% lower than JP‐10, suggesting limited potential as a high‐energy aviation fuel. Conversely, pentalenane demonstrated a volume energy density of 40.55 MJ L^−1^, 2% higher than JP‐10, while presilphiperfol‐1‐ane exhibited a volume energy density of 39.24 MJ L^−1^, less than 1% lower than JP‐10. These results exhibit the potential of presilphiperfol‐1‐ane and pentalenane as promising candidates for new high‐energy aviation fuels.

**Table 3:** Measurement of important performance parameters of sesquiterpene molecules, including volume energy density, net heats of combustion, density, and freezing point

Compound	Structure	Performance	Volume energy density	Net heats of combustion	Density	Freezing point
			[MJ L^−1^]	[MJ kg^−1^]	[g cm^−3^, 20 °C]	[°C]
Pentalenane (hydrogenation)	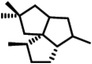	Actual	40.55	45.4	0.8932	<−67
		Predicted	38.35	42.47	0.903	25.794
		SD%	5.74	6.9	−1.09	−359.75
Farnesane (hydrogenation)	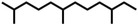	Actual	33.93	44.08	0.7698	‐52
		Predicted	34.56	44.14	0.783	−60.306
		SD%	−1.83	−0.14	−1.69	11.1
Presilphiperfol‐1‐ane (hydrogenation)	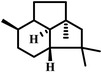	Actual	39.24	42.7	0.919	‐47
		Predicted	38.98	42.83	0.91	43.535
		SD%	0.68	−0.3	0.99	−253.9
Pentalenene	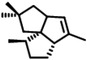	Actual	40.73	44.79	0.9093	−63.5
Pentalenene cyclopropanation	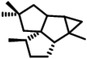	Actual	38.43	42.75	0.8991	<−53
JP‐10^[^ ^22^, ^28^, ^30^, ^31^ ^]^	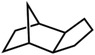	Actual	39.6	42.127	0.94	<−79

The freezing point data revealed significant discrepancies between predicted and measured values. Specifically, the predicted freezing point of pentalenane was projected to be 25.8 °C, whereas the actual measured value was lower than −60 °C. Likewise, the predicted freezing point of presilphiperfol‐1‐ane was estimated at 43.5 °C, while the measured value was −47 °C. Furthermore, the predicted freezing point of farnesane was −60 °C, whereas the actual measured value was −52 °C. The predicted value differed greatly from the measured value and may be due to the degree of chiral isomerism, which was not limited in the calculation. The predicted values obtained were the combined parameters of various isomers. Therefore, further the chiral structure of the compounds can be further clarified to obtain more accurate freezing point prediction values.

### 2.6 Performance Testing of Pentalenene and Cyclopropanation of Pentalenene

Pentalenene has been identified as a sesquiterpene with superior performance properties compared to JP‐10 in certain parameters. Its unique double bond structure allows for the introduction of different groups through addition reactions, enabling the exploration of derivatization modification effects on sesquiterpene properties. The cyclopropanation has been shown to improve the compound performance.^[^ ^44^, ^45^ ^]^ In our study, we conducted cyclopropanation on pentalenene and evaluated its effects on volumetric energy density, net heats of combustion, and density (Table 3). The measured values for pentalenene were found to be 40.73 MJ L^−1^, 44.79 MJ kg^−1^, and 0.9093 g cm^−3^ (20 °C), while the values for pentalenene after cyclopropanation were measured to be 38.43 MJ L^−1^, 42.75 MJ kg^−1^, and 0.8991 g cm^−3^ (20 °C). Despite the lower measurements in volumetric energy density, net heats of combustion, and density for pentalenene after cyclopropanation, it is possible that pentalenene after cyclopropanation may possess other superior properties compared to pentalenene. Additionally, the cyclopropanation reactions could potentially eliminate the adverse effects of double bonds on fuel stability, offering significant potential for optimizing the properties of sesquiterpenes.

### 2.7 Summary and Discussion

Sesquiterpenes have gained increasing attention as promising precursors for advanced high‐energy aviation fuel due to their structural diversity, suitable carbon number, and active olefin functionality. The exploration of sesquiterpenes with diverse structural characteristics could serve as a valuable reference for the discovery and design of novel aviation fuel molecules.

In this study, we established a comprehensive database of 122 sesquiterpene skeletal molecules using a combination of group contribution and Gaussian quantum chemistry methods. Then we calculated various performance parameters for these sesquiterpene skeletons, including volumetric energy density, net heats of combustion, freezing point, flash point, density, and specific impulse. Based on structural characteristics and predicted performance, 17 compounds were selected and their gene functions were characterized. Through metabolic engineering in yeast, we successfully achieved overproduction of nine sesquiterpene compounds, all of which reached the highest reported levels to date. Subsequently, pentalenene, presilphiperfol‐1‐ene, and *α*‐farnesene were isolated and purified, and these three sesquiterpenes were further hydrogenated to test their performance parameters. To the best of our knowledge, this is the first reported combustion performance test of biosynthetic sesquiterpenes.

Our results revealed that although farnesane exhibits the highest combustion heat, its low density leads to a low volumetric energy density, which may limit its potential as an aviation fuel candidate. On the other hand, presilphiperfol‐1‐ane, with a volumetric energy density of 39.24 MJ L^−1^, comparable to that of JP‐10, shows promise as a new type of aviation fuel. Notably, biosynthesized presilphiperfol‐1‐ene is solid at 20°C, while presilphiperfol‐1‐ane is liquid at the same temperature, indicating that presilphiperfol‐1‐ene could be utilized in different environments after hydrogenation. Moreover, both pentalenene and pentalenane exhibit higher volumetric energy densities than JP‐10 (39.6 MJ L^−1^), suggesting that pentalenene holds great potential as a high‐energy aviation fuel. However, the predicted freezing point values were found to be inaccurate, and further optimization of the prediction method is warranted in future studies.

Furthermore, the molecular structure of sesquiterpenes can be modified using unsaturated bonds, and cyclopropanation has been demonstrated to increase the net heats of combustion of fuels.^[^ ^45^ ^]^ However, the performance of pentalenene after cyclopropanation was found to be not ideal, possibly due to structural changes that are not conducive to increased combustion heat compared to its original structure. Nevertheless, given the structural diversity of sesquiterpenes, other sesquiterpene structures may yield different results, and cyclopropanation could still potentially enhance the fuel performance of other compounds. Other derivative reactions may also yield positive effects, indicating that the unsaturated double bond of pentalenene offers versatility for various applications in different environments.

In conclusion, our systematic evaluation of the potential of sesquiterpenes as high‐energy aviation fuels demonstrates a technological process that combines calculations and practical tests. Although the cost of biosynthesized sesquiterpenes using glucose as a carbon source is currently higher than conventional fuels, the diverse fuel properties conferred by biosynthetic sesquiterpenes create new possibilities for biofuel development and guide a brand‐new direction for the development of carbon neutralization strategies and fuel fields.

## 3 Experimental Section

### Materials and Reagents

Prime STAR GXL DNA Polymerase (TaKaRa, Kyoto, Japan) and Phusion High‐Fidelity polymerase (New England Biolabs, NEB; Ipswich, MA) were used for fragments amplification. Purification was performed using the Hipure Gel Pure DNA Mini Kit (Magen, Guangzhou, China). The polymerase chain reaction primers were synthesized by GeneCreate. Sesquiterpenes synthases genes were codon‐optimized for *S. cerevisiae* and synthesized by GeneScript (GenScript USA Inc., Piscataway, NJ). Enzymes for Goldengate were purchased from New England Biolabs. Yeast extracts and tryptone used for preparing Luria–Bertani medium were purchased from Oxoid (Hampshire, England, UK). Yeast extracts and tryptone used for YPD (2% tryptone, 1% yeast extract, and 2% glucose) and YPDHG medium (2% tryptone, 1% yeast extract, and 1% glucose, 1% galactose) were purchased from Angel Yeast Co., Ltd (Hubei, China). Salts, glucose, and galactose were purchased from Sinopharm Chemical Reagent Co., Ltd. (Wuhan, China).

### Plasmids and Strains

All plasmids, strains, primers, and fragments used in this study are listed in Tables S7, S8, and S9 (Supporting Information). Plasmids were constructed using yeast assembly^[^ ^46^ ^]^ or golden gate method.^[^ ^47^ ^]^ Gene deletion and genomic integrations for engineered yeast strains construction were conducted using LiAc/SS carrier DNA/PEG method^[^ ^48^ ^]^ and verified using diagnostic polymerase chain reaction.

The plasmid p1‐vector was constructed by yeast assembly method. For achieving the biosynthesis of 17 sesquiterpene compounds in *S. cerevisiae*, terpene synthase genes were amplified from gene templates synthesized by GenScript to replace the LacZ fragments in the P1‐vector plasmid using golden gate method; the reconstructed plasmids were expressed in the strain JCR27 to construct the engineering strains.

For the efficient synthesis of nine target sesquiterpene compounds in *S. cerevisiae*, the plasmid p2‐vector and p3‐vector were constructed by yeast assembly method; some terpene synthase genes were amplified to replace the LacZ fragments in the P2‐vector or P3‐vector plasmid using golden gate method; some terpene synthase genes were amplified to constitute new plasmids with other fragments. All the plasmids were digested into fragments using restriction enzymes and integrated into the chromosome of the engineering strains utilizing LiAc/SS carrier DNA/PEG method.

### Ninety‐Six‐Deep Well Microtiter Plates Cultivation

Single colonies of recombinant yeast strains harboring plasmid each with different sesquiterpene synthase were picked into 96‐deep well microtiter plates (1.2 mL, Axygen Inc., Union City, CA) with 350 µL Sc‐ura medium and cultured for 24 h at 30 °C in a rotary shaker (Multitron pro, Infors HT, China) at 999 rpm. Then 15 µL seed broth was transferred into 350 µL YPDHG with 20% v/v IPM overlay and grown for 72 h at 30 °C in a rotary shaker (Multitron pro, Infors HT, China) at 999 rpm. In the end, the 96‐deep well microtiter plates were centrifuged at 4000 rpm for 10 min using the centrifuge (Allegra X‐15R, Beckman coulter, Brea, CA); further, the IPM were isolated for detection.

### Shake‐Flask Fermentation and Fed‐Batch Fermentation

The 20 µL seed cultures were prepared by inoculating glycerol stock into 5 mL YPD medium and cultured overnight at 30 °C in a rotary shaker (ZHWY‐211C, ZhiCheng, Shanghai) at 220 rpm. Seed cultures were transferred into 50 mL YPD medium with initial OD_600_ = 0.1, and further, 10% v/v IPM was added into the medium to capture the sesquiterpene. Fermentation was performed at 30 °C in a rotary shaker at 220 rpm for 72 h. The 50 mL fermentation broth was poured into a 50 mL centrifuge tubes and centrifuged at 5000 rpm for 6 min.

Fifteen‐liter fermenter (Bailun bio, Shanghai, China) was used for fed‐batch fermentation based on a previously method.^[^ ^37^ ^]^ The medium contained 40 g glucose, 15 g (NH_4_)2SO_4_, 8.0 g KH2PO_4_, 6.2 g MgSO_4_·7H_2_O, 15 mL vitamin solution, and 12 mL trace metals solution per liter. Trace metals solution contained: 5 g ethylenediaminetetraacetic acid, 5.75 g ZnSO_4_∙7H_2_O, 0.32 g MnCl_2_∙4H_2_O, 0.75 g CuSO_4_∙5H_2_O, 0.47 g CoCl_2_∙6H_2_O, 0.48 g Na_2_MoO_4_∙2H_2_O, 2.9 g CaCl_2_∙2H_2_O, and 2.8 g FeSO_4_∙7H_2_O per liter. The vitamin solution contained biotin, 0.1 g; calcium pantothenate, 3.0 g; nicotinic acid, 1.0 g; myo‐inositol, 25.0 g; thiamine hydrochloride, 1.5 g; pyridoxol hydrochloride, 1 g; and p‐aminobenzoic acid, 0.2 g per liter. Seven‐hundred milliliter of feeding solution consisted of 500 g L^−1^ glucose, 9 g L^−1^ KH_2_PO4, 5.2 g L^−1^ MgSO_4_∙7H_2_O, 3.5 g L^−1^ K_2_SO_4_, 0.28 g L^−1^ Na_2_SO_4_, 15 mL vitamin solution, and 12 mL trace metals solution. Two liter of feeding solution consisted of 800 g L^−1^ sucrose, 9 g L^−1^ KH_2_PO_4_, 5.2 g L^−1^ MgSO_4_∙7H_2_O, 3.5 g L^−1^ K_2_SO_4_, 0.28 g L^−1^ Na_2_SO_4_, 12 mL trace metal solution, and 15 mL vitamin solution. Twenty milliliter seed cultures were prepared by inoculating glycerol stock into 5 mL of YPD medium overnight at 30 °C in a rotary shaker at 220 rpm. Then, 2% seed cultures were transferred to 200 mL YPD medium at 30 °C with shaking at 220 rpm for 14 h. Further, 10% seed cultures were inoculated into 7 L fermentation medium in a 15 L fermenter for fed‐batch fermentation at 30 °C, with the pH maintained at 5.0 using NH_3_·H_2_O. Fermentation was performed at an agitation speed between 300 and 500 rpm and an airflow rate ranging from 0.5 to 2 Nm^3^ h^−1^. A 10% volume of IPM was added for extracting sesquiterpene production.

### Detection of Residual Glucose, and Ethanol in Fermentation Broth

Residual glucose and ethanol concentrations in the medium during fed‐batch fermentation were determined using a Bioanalyzer (SBA‐40C, Shandong Academy of Sciences, China).

### Detection of Sesquiterpenes with GC‐MS

IPM layers were collected after fermentation and centrifuged for 6 min at 10 000 rpm to remove impurities. The concentration of the product was diluted with hexane for GC‐MS analysis. The initial column temperature for the GC‐MS condition was 50 °C, maintained for 1 min, rising to 280 °C for 1 min at 15 °C, and then at 20 to 300°C and maintained for 2 min. The products were analyzed in total ion monitoring mode and selective ion monitoring mode (m/z 161). The location of the product can be determined by the database alignment, and the standard curve was used for the quantification. The standards of *α*‐farnesene, pentalenene, presilphiperfol‐1‐ene, epi‐isozizaene, protoilludene, *α*‐santalene, and *β*‐copaene were purified from the experiments. Thujopsene and *α*‐barbatene were quantified using pentalenene as internal standard.

### Product Purification and NMR Characterization

When the fermentation finished, cell broth was centrifuged for 10 min at 10 000 rpm; then, the IPM overlay was collected. Product and IPM were separated through a 1.2 m height fractionating tower filled with 4 × 4 mm^2^ Dixon Ring under vacuum distillation. The pure products were obtained as colorless oil for nuclear magnetic resonance (NMR) analysis. The structure of the isolated sesquiterpenes dissolved in CDCl_3_ were analyzed by ^1^H and using a 400 MHz or 300 MHz NMR spectrometer. NMR spectrums of these compounds were shown in Additional file 1: Figures S18–S23 (Supporting Information).

### Physicochemical and Energy Properties Calculation of the Sesquiterpene Products

Physicochemical properties and energy properties of these hydrogenated compounds were calculated using the quantum chemical method and group contribution method.^[^ ^49^ ^]^ The net heat of combustion was calculated using the quantum chemical method. Molecular structures were optimized at the Density functional theory level of (B3LYP/6‐31G (d, p)) by using Gaussian 09. The atomic location data are given by Cartesian coordinates XYZ formats. Frequency calculations at the same computational level were performed to determine whether the optimized structure is a thermodynamic stable state structure, and the standard enthalpy value was calculated. Then, the net heat of combustion can be calculated. Density, flash point, and other physicochemical properties were calculated by the values of corresponding groups using group contribution method. According to the molecular structure of the fuel, the molecular properties are regarded as the sum of the contributions of the groups that make up them to the physical properties. The correlation between the contribution values of various groups and the physical properties is deduced by using the thermodynamic principle. Then, the UNIQUAC model is used to calculate the interaction parameters between substances, including two parts, one is the chemical process caused by the change of various chemical bond within the molecule. The other part is determined by the intermolecular interactions caused by changes in intermolecular forces.

### Hydrogenation Reaction

To a stirred solution of feedstock (14.3 g) in EtOH (80 mL) was added 5 wt% Pd/C (0.7 g) at room temperature. Then, air in the reaction bottle was replaced with nitrogen three times, and the nitrogen in the bottle was replaced with hydrogen three times. After being stirred with hydrogen balloon for 4 h, the Pd/C was removed by filtration. After removal of the solvent, the products was obtained with the yield of ≈90–95% (14.4 g farnesane, 95% yield; 14.0 g presilphiperfol‐1‐ane, 92% yield; 13.4 g pentalenane, 90% yield). NMR spectrums of these compounds were shown in Additional file 1: Figures S24 and S25 (Supporting Information).

### Cyclopropanation Reaction

Charge pentalanene (20.4 g, 100 mmol, and 1.0 eq) and 1,2‐dichloromethane (200 mL and 5 vol) were placed in 500 mL 3‐necked round bottom flask and cooled to 0 °C under N2. Et2Zn solution (2 m, 100 mL, 2 eq) was added dropwise to the solution (maintain system temperature ≤5 °C) and then left to stir at 0 °C for at least 30 min. CH2I2 (80.3 g, 300 mmol, 3 eq) was added dropwise to the mixture above (maintain system temperature ≤5 °C), and then, the mixture was stirred for 7 d at room temperature. The reaction was monitored by GC‐MS. Quench reaction by saturated NH4Cl (150 mL); the separated aqueous layer was extracted by hexane (50 mL × 3). The solvent was removed under vacuum. Purification by reduced‐pressure distillation afforded product (20.5 g, 95% yield) as colorless oil. NMR spectrums of the compound were shown in Additional file 1: Figure S26 (Supporting Information).

### Performance Testing of the Sesquiterpene Product

Density was determined in accordance with GB/T 1884 using the densitometer (Mettler DE40). Further, 10 mL samples were transferred to a measuring cylinder, and a densitometer was placed in the samples, and the densitometer scale values were read when the densitometer was stationary.

Freezing points were determined in accordance with GB/T 2430 using the ultralow temperature test chamber. Further, 10 mL samples were poured into a double‐walled glass test tube. The temperature was recorded when the crystals began to appear in the samples.

Net heats of combustion were determined in accordance with GB/T 384 using oxygen bomb calorimeter. Then, 1 g of the samples were loaded in the bottle (300 mL) and burnt in oxygen at 2 Mpa. Water temperature changes were measured to determine the net heats of combustion.

### Identification of Presilphiperfol‐1‐ene Synthase

Based on the whole genome sequencing results of C. gloeosporioides ES026, combined with bioinformatic analysis, potential terpene synthase genes were selected, and the cDNA sequences were obtained by integrating the exons of the predicted target genes through genomic Polymerase Chain Reaction, Reverse transcription‐Polymerase Chain Reaction, and gene synthesis. The predicted genes were detected to have the function of terpene synthase by heterologous expression in *E. coli* and *S. cerevisiae*, respectively. Finally, the terpene synthase Cgl06493‐COP were obtained, which can catalyze the synthesis of presilphiperfol‐1‐ene.

## Conflict of Interest

The authors declare no conflict of interest.

## Author Contributions

Y.H., Z.Y., X.W., and G.Y. contributed equally to this work. Y.H., Z.Y., T.L., K.S., H.J., and Z.D. designed the work. X.W., G.Y., and M.Y. performed the energy calculation of derivatives of sesquiterpenoids and actual performance testing. Y.H., Z.Y., X.W., G.Y., J.D., and J.L. constructed strains and screened the terpene synthases. X.S. discovered presilphiperfol‐1‐ene synthase. Y.H., X.W., and G.Y. performed the fermentation and product purification. All authors prepared the manuscript.

## Supporting information

Supporting Information

## Data Availability

Research data are not shared.
