# Supplementary material for: Systematic Mining and Evaluation of the Sesquiterpene Skeletons as High Energy Aviation Fuel Molecules
Source: Adv Sci (Weinh). 2023 Jun 4;10(23):2300889. doi: 10.1002/advs.202300889 (PMC10427387; doi:10.1002/advs.202300889)
Supplement: Supplementary file 1 — Supporting Information [file ADVS-10-2300889-s001.pdf]

ADVANCED SCIENCE
Open Access

Supporting Information

for *Adv. Sci.*, DOI 10.1002/advs.202300889

Systematic Mining and Evaluation of the Sesquiterpene Skeletons as High Energy Aviation Fuel Molecules

Yanglei Huang, Ziling Ye, Xiukun Wan, Ge Yao, Jingyu Duan, Jiajia Liu, Mingdong Yao, Xiang Sun, Zixin Deng, Kun Shen, Hui Jiang* and Tiangang Liu**

Supplementary Information

Systematic mining and evaluation of the sesquiterpene skeletons as high energy aviation fuel molecules

Yanglei Huang[1, #], Ziling Ye[1, #], Xiukun Wan[2, #], Ge Yao[2, #], Jingyu Duan[1], Jiajia Liu[2], Mingdong Yao[3], Xiang Sun[1], Zixin Deng[1,4], Kun Shen[1, *], Hui Jiang[2, *], Tiangang Liu[1, 5, *]

[1]Key Laboratory of Combinatorial Biosynthesis and Drug Discovery, Ministry of Education and School of Pharmaceutical Sciences, Wuhan University, Wuhan 430071, China.
[2]State Key Laboratory of NBC Protection for Civilian, Beijing, 102205, China.
[3]Frontier Science Center for Synthetic Biology and Key Laboratory of Systems Bioengineering (Ministry of Education), School of Chemical Engineering and Technology, Tianjin University, Tianjin 300072, China; Frontier Technology Research Institute, Tianjin University, Tianjin 301700, China.
[4]State Key Laboratory of Microbial Metabolism, School of Life Sciences and Biotechnology, Shanghai Jiao Tong University, Shanghai 200030, China
[5]Hubei Engineering Laboratory for Synthetic Microbiology, Wuhan Institute of Biotechnology, Wuhan 430075, China.

*Corresponding author at School of Pharmaceutical Sciences, Wuhan University, 185 Donghu Road, Wuhan 430071, China. Email: liutg@whu.edu.cn (T. Liu).
*Corresponding author at State Key Laboratory of NBC Protection for Civilian, Beijing, 102205, China. Email: jiangtide@sina.cn (H. Jiang).
*Corresponding author at School of Pharmaceutical Sciences, Wuhan University, 185 Donghu Road, Wuhan 430071, China. Email: kun.shen@whu.edu.cn (K. Shen).
[#]These authors contributed equally to this work.

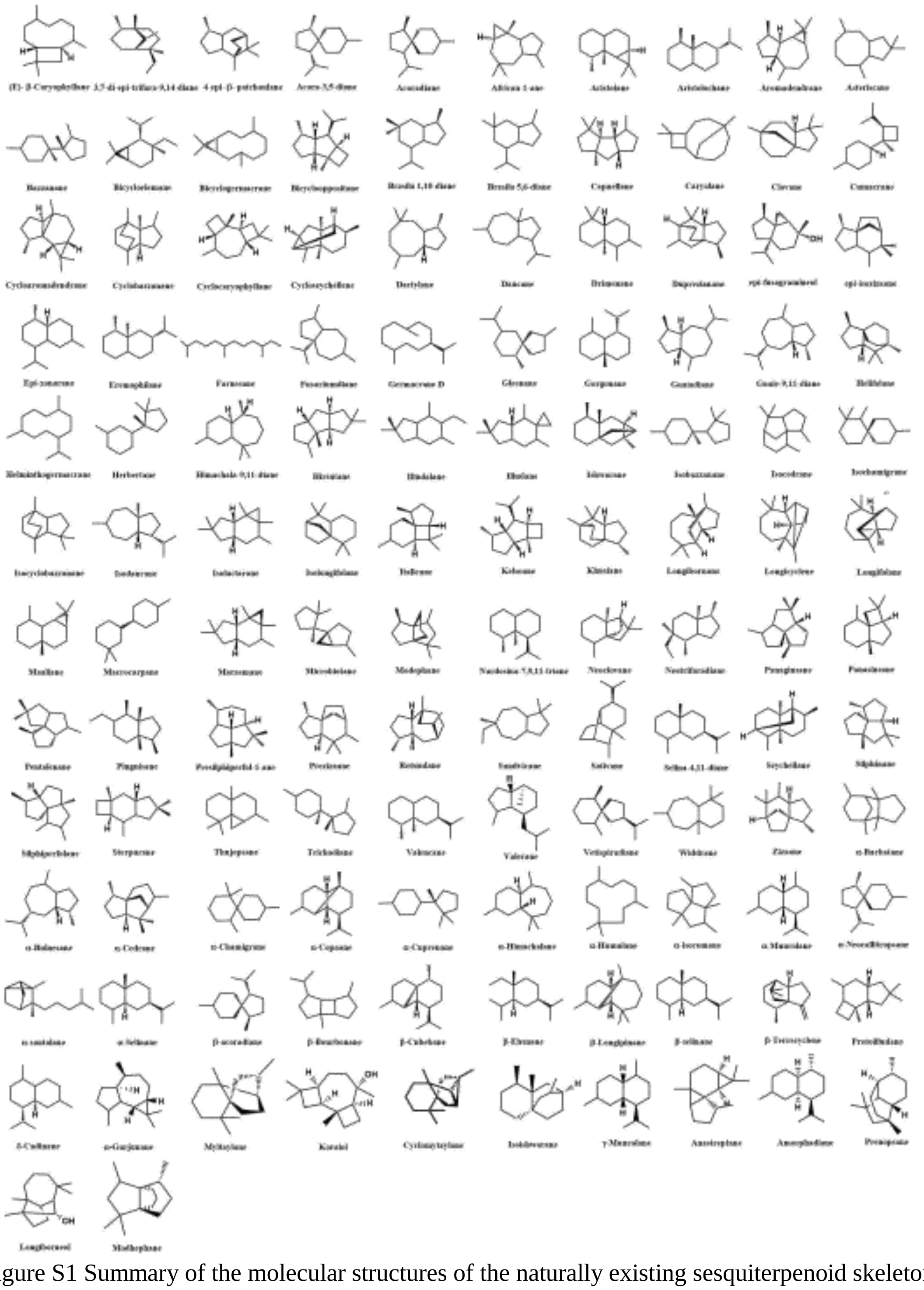

Figure S1 Summary of the molecular structures of the naturally existing sesquiterpenoid skeleton

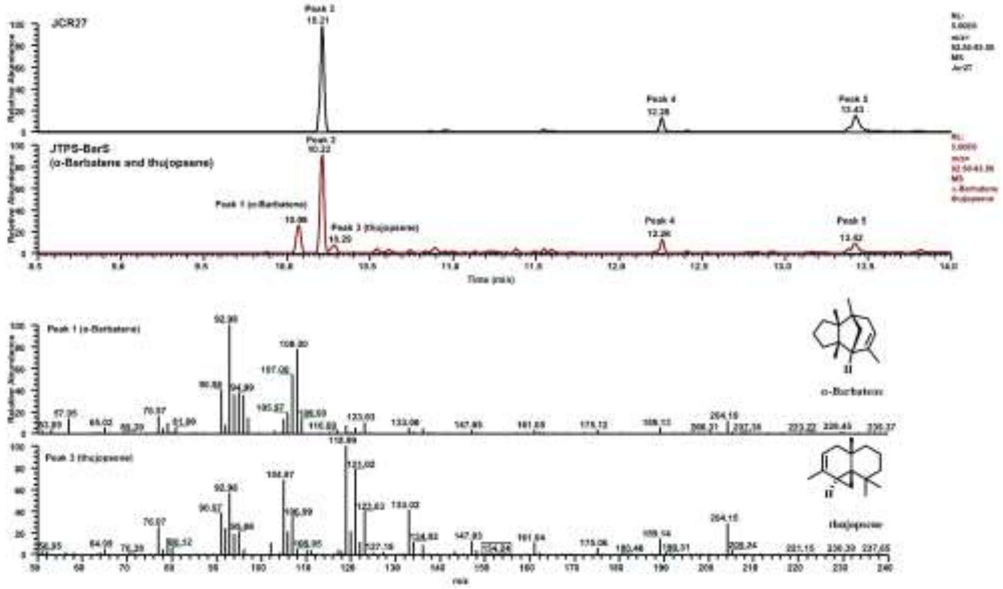

Figure S2 Retention time and fragmentation pattern of biosynthetic α-Barbatene (peak 1, RT: 10.08 min) and thujopsene (peak 3, RT: 10.29 min), the fermentation broth from strain JCR27 was used as control, except for the target products, there were other products could be synthesized, including the products synthesized by strain JCR27 (peak 2, peak 4 and peak 5).

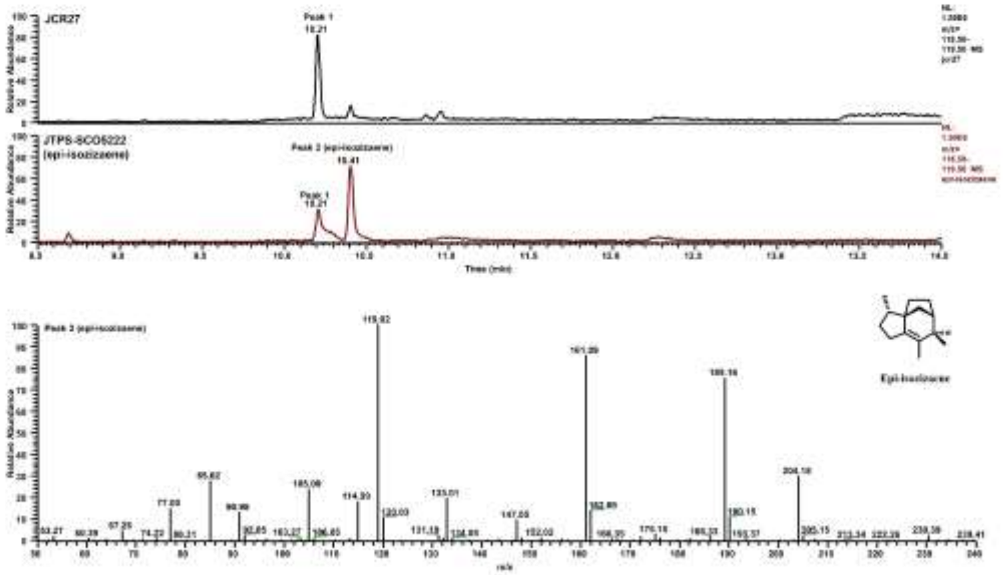

Figure S3 Retention time and fragmentation pattern of biosynthetic epi-isozizaene (peak 2, RT: 10.41 min), the fermentation broth from strain JCR27 was used as control, except for the target product, there were other products could be synthesized, including the product synthesized by strain JCR27 (peak 1).

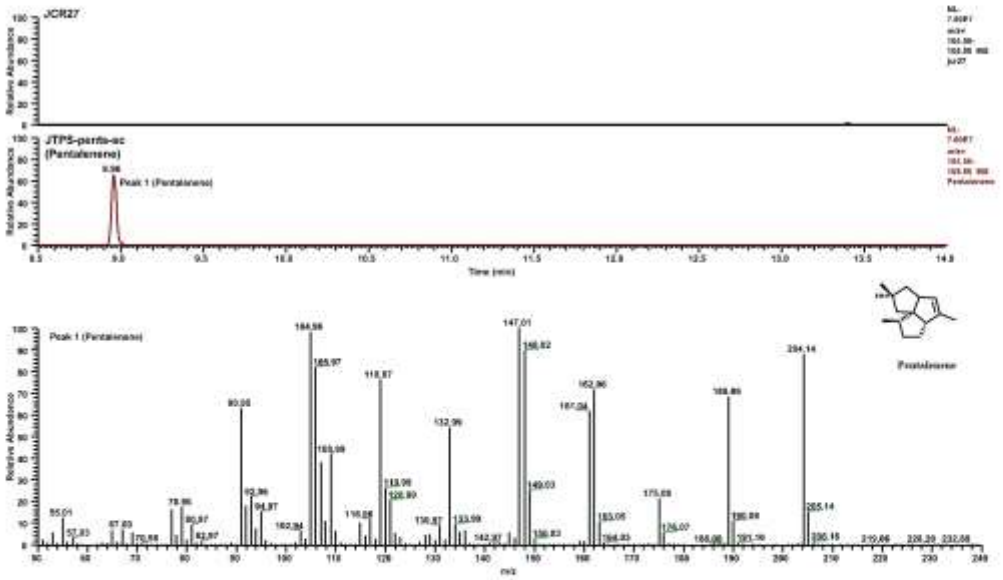

Figure S4 Retention time and fragmentation pattern of biosynthetic Pentalenene (peak 1, RT: 8.96 min), the fermentation broth from strain JCR27 was used as control.

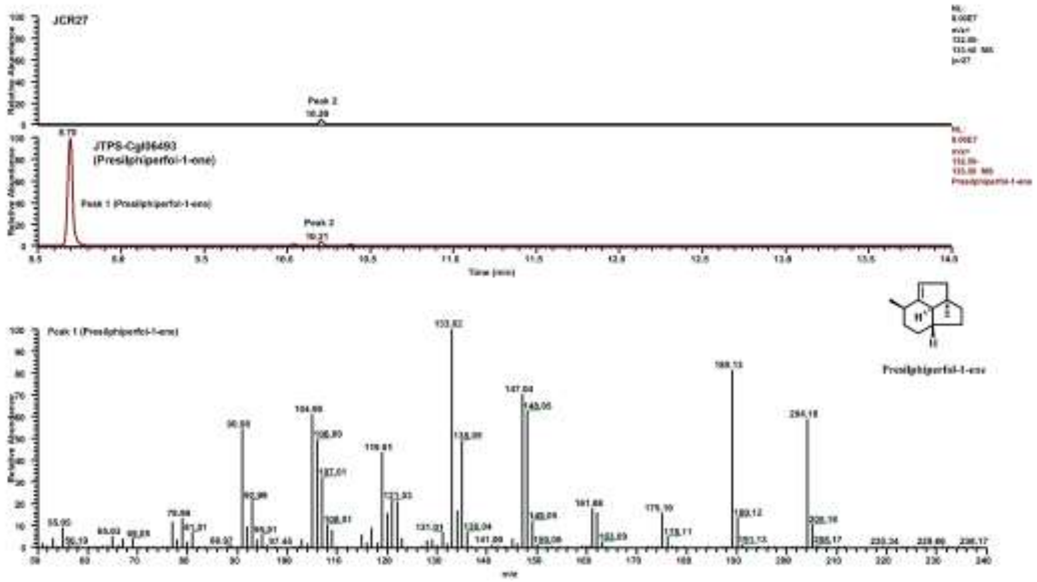

Figure S5 Retention time and fragmentation pattern of biosynthetic Presilphiperfol-1-ene (peak 1, RT: 8.70 min), the fermentation broth from strain JCR27 was used as control, except for the target product, there were other products could be synthesized, including the product synthesized by strain JCR27 (peak 2).

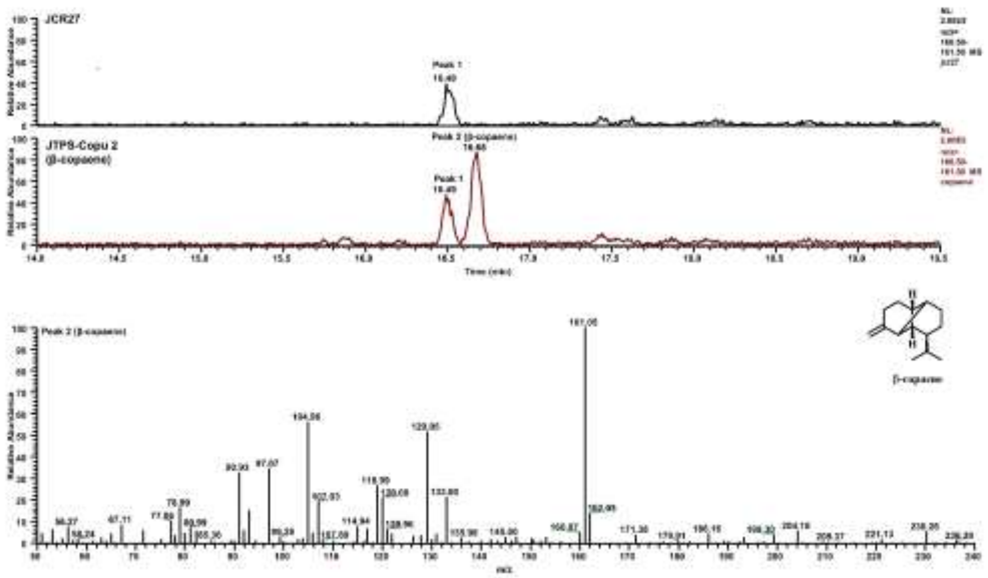

Figure S6 Retention time and fragmentation pattern of biosynthetic β-copaene (peak 2, RT: 16.68 min), the fermentation broth from strain JCR27 was used as control, except for the target product, there were other product could be synthesized, including the product synthesized by strain JCR27 (peak 1).

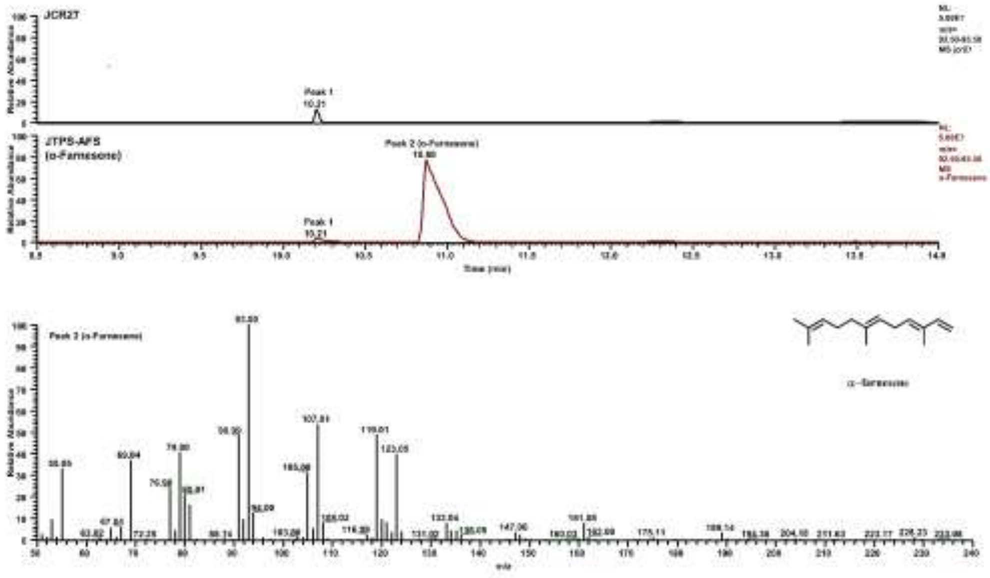

Figure S7 Retention time and fragmentation pattern of biosynthetic α-Farnesene (peak 2, RT: 10.88 min), the fermentation broth from strain JCR27 was used as control, except for the target product, there were other product could be synthesized, including the product synthesized by strain JCR27 (peak 1).

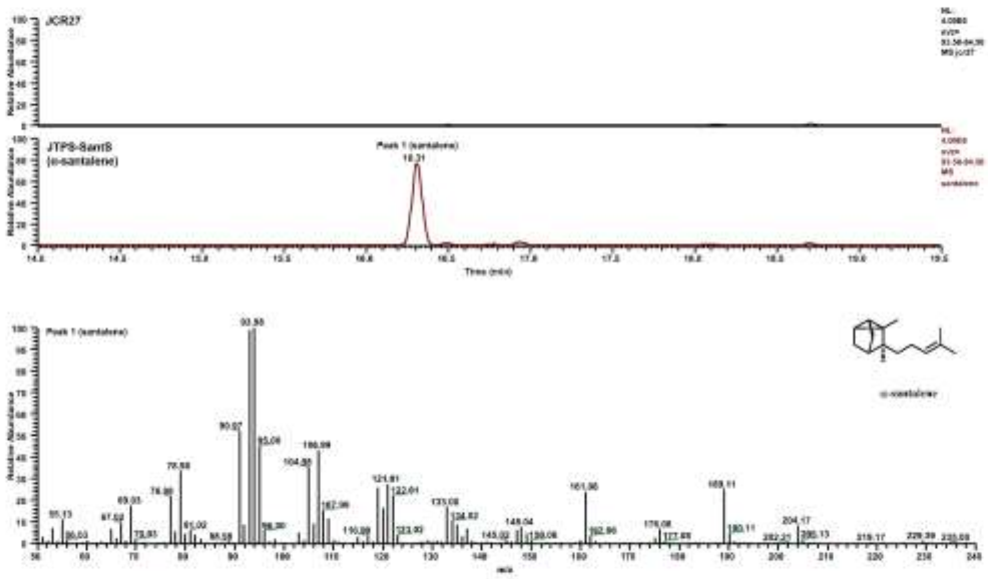

Figure S8 Retention time and fragmentation pattern of biosynthetic α-santalene (peak 1, RT: 16.31 min), the fermentation broth from strain JCR27 was used as control.

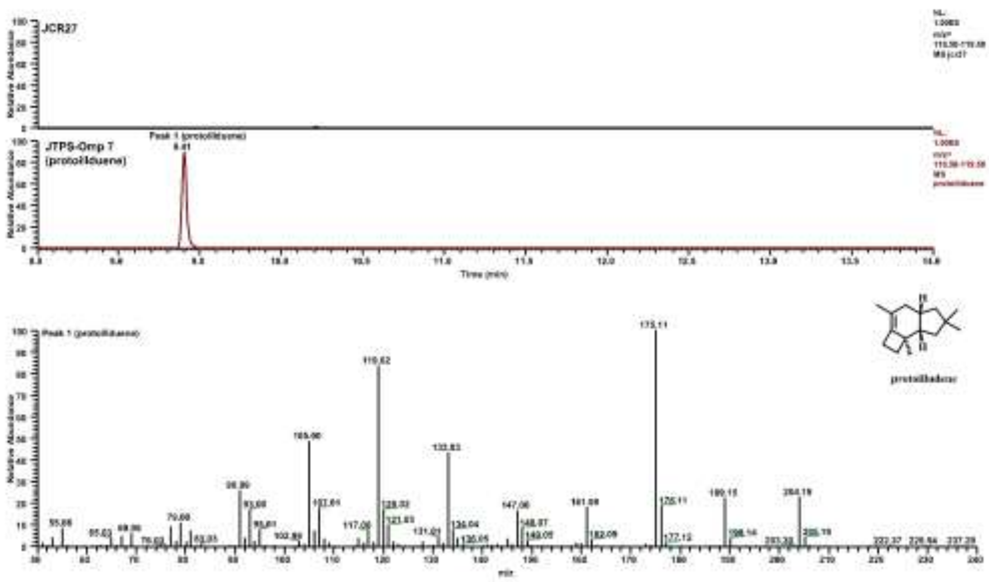

Figure S9 Retention time and fragmentation pattern of biosynthetic protoillduene (peak 1, RT: 9.41 min), the fermentation broth from strain JCR27 was used as control.

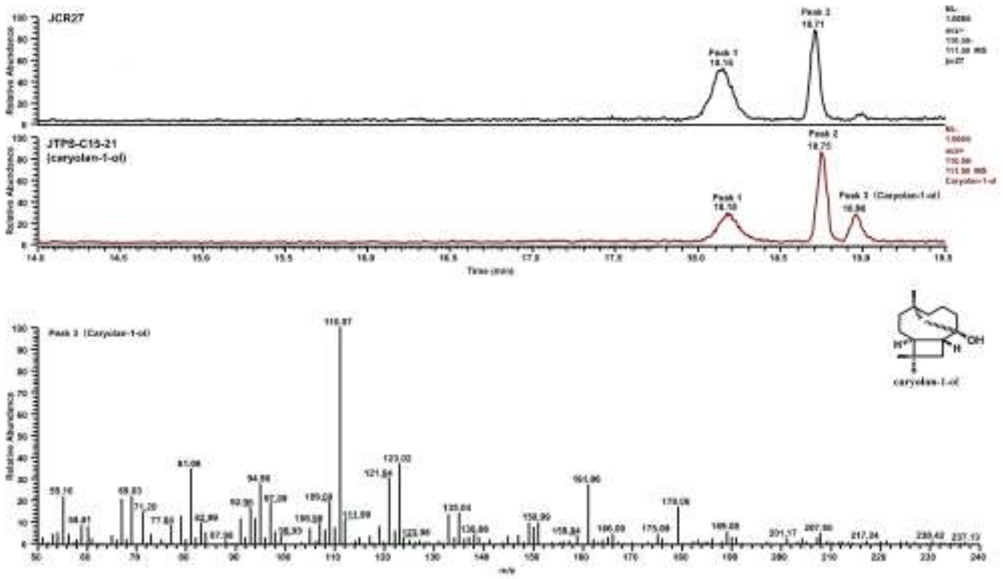

Figure S10 Retention time and fragmentation pattern of biosynthetic caryolan-1-ol (peak 3, RT: 18.96 min), the fermentation broth from strain JCR27 was used as control, except for the target product, there were other products could be synthesized, including the products synthesized by strain JCR27 (peak 1 and peak 2).

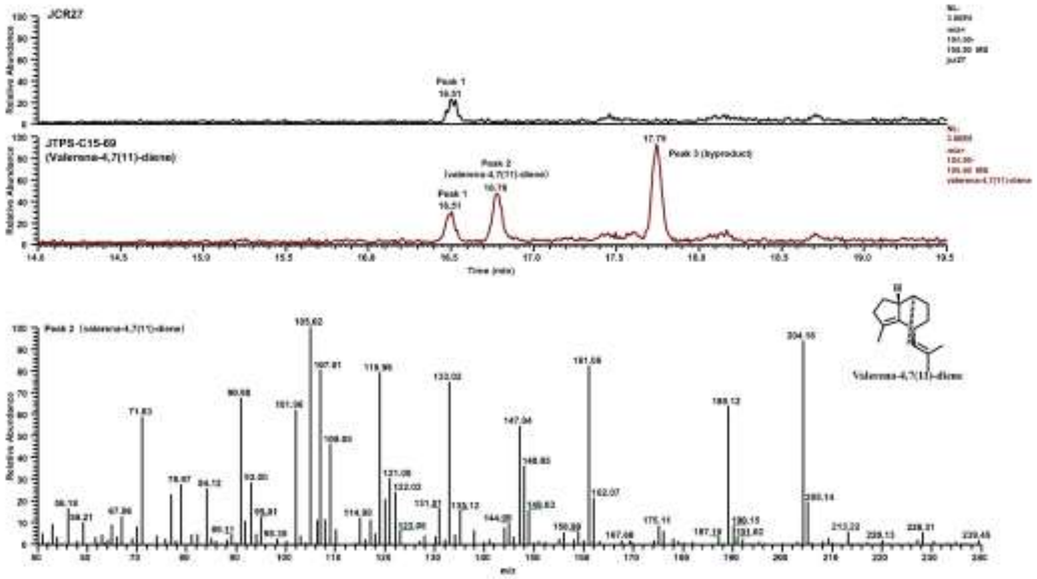

Figure S11 Retention time and fragmentation pattern of biosynthetic Valerena-4,7(11)-diene (peak 2, RT: 16.78 min), the fermentation broth from strain JCR27 was used as control, except for the target product, there were other products could be synthesized, including the product synthesized by strain JCR27 (peak 1) and the byproduct synthesized by strain JTPS-C15-69 (peak 3).

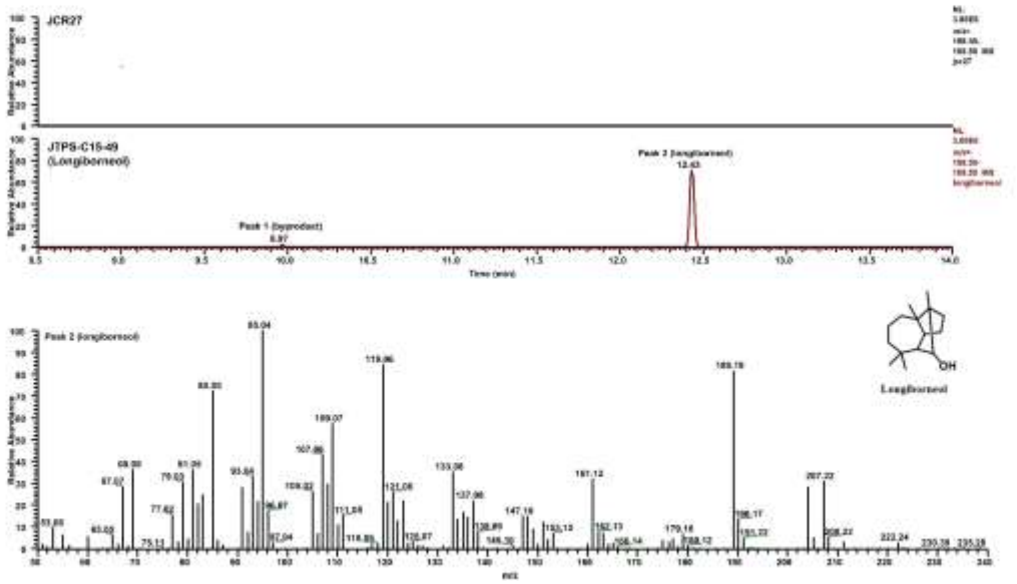

Figure S12 Retention time and fragmentation pattern of biosynthetic Longiborneol (peak 2, RT: 12.43 min), the fermentation broth from strain JCR27 was used as control, except for the target product, there were other product could be synthesized, including the byproduct synthesized by strain JTPS-C15-49 (peak 1).

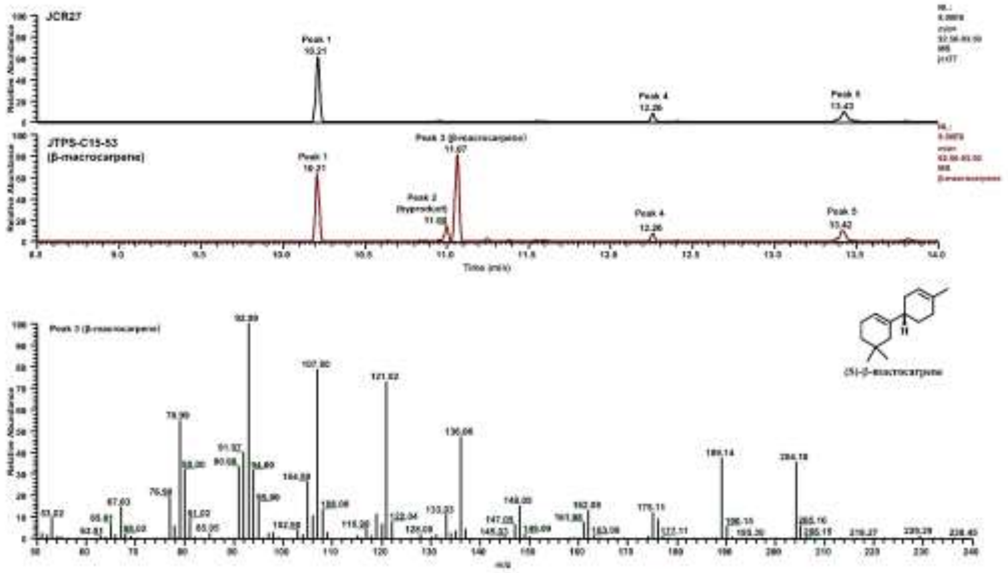

Figure S13 Retention time and fragmentation pattern of biosynthetic β-macrocarpene (peak 3, RT: 11.07 min), the fermentation broth from strain JCR27 was used as control, except for the target product, there were other products could be synthesized, including the products synthesized by strain JCR27 (peak 1, peak 4 and peak 5) and the byproduct synthesized by strain JTPS-C15-53 (peak 2).

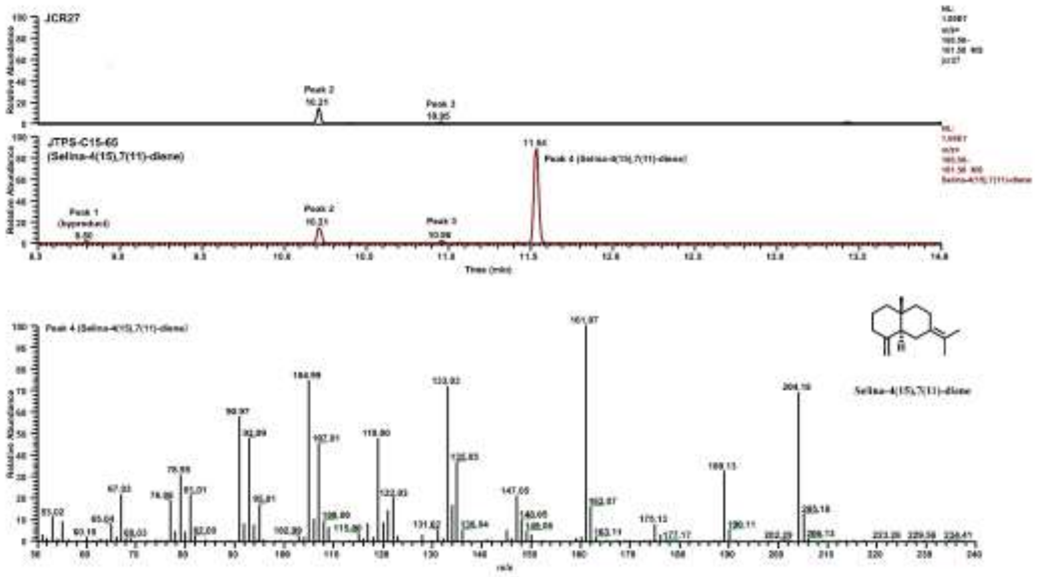

Figure S14 Retention time and fragmentation pattern of biosynthetic Selina-4(15),7(11)-diene (peak 4, RT: 11.54 min), the fermentation broth from strain JCR27 was used as control, except for the target product, there were other products could be synthesized, including the products synthesized by strain JCR27 (peak 2 and peak 3) and the byproduct synthesized by strain JTPS-C15-65 (peak 1).

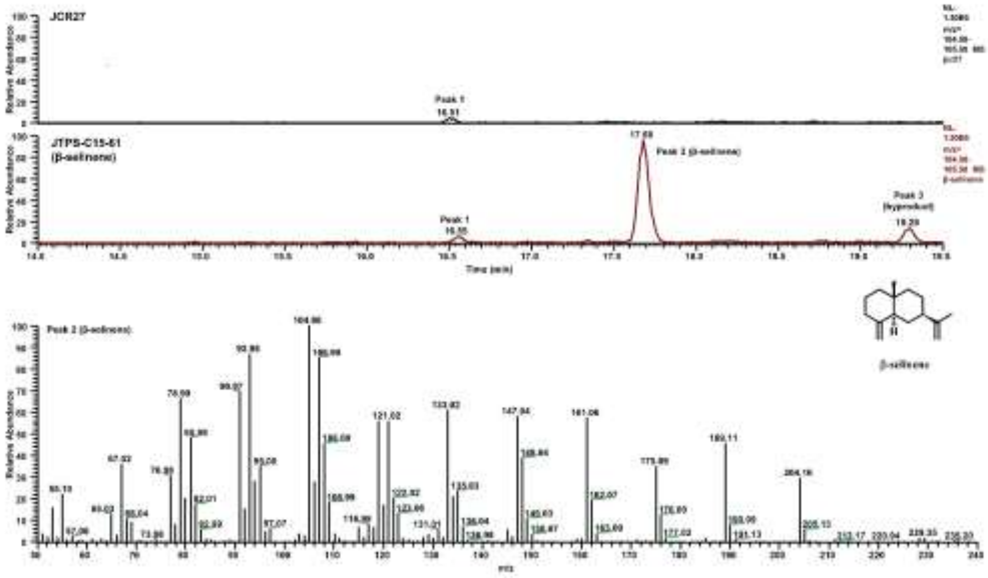

Figure S15 Retention time and fragmentation pattern of biosynthetic β-selinene (peak 2, RT: 17.68 min), the fermentation broth from strain JCR27 was used as control, except for the target product, there were other products could be synthesized, including the product synthesized by strain JCR27 (peak 1) and the byproduct synthesized by strain JTPS-C15-61 (peak 3).

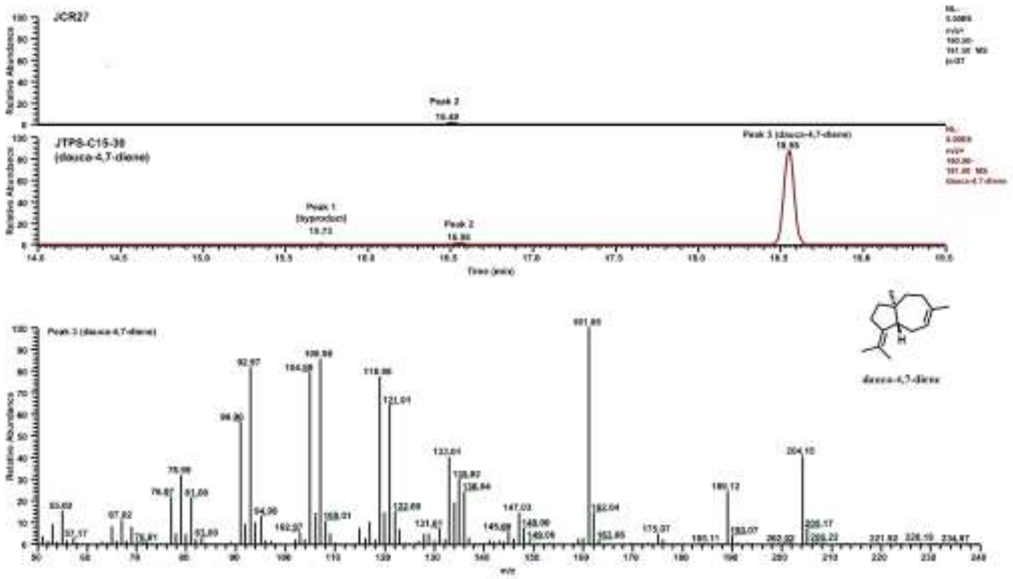

Figure S16 Retention time and fragmentation pattern of biosynthetic dauca-4,7-diene (peak 3, RT: 18.55 min), the fermentation broth from strain JCR27 was used as control, except for the target product, there were other products could be synthesized, including the product synthesized by strain JCR27 (peak 2) and the byproduct synthesized by strain JTPS-C15-30 (peak 1).

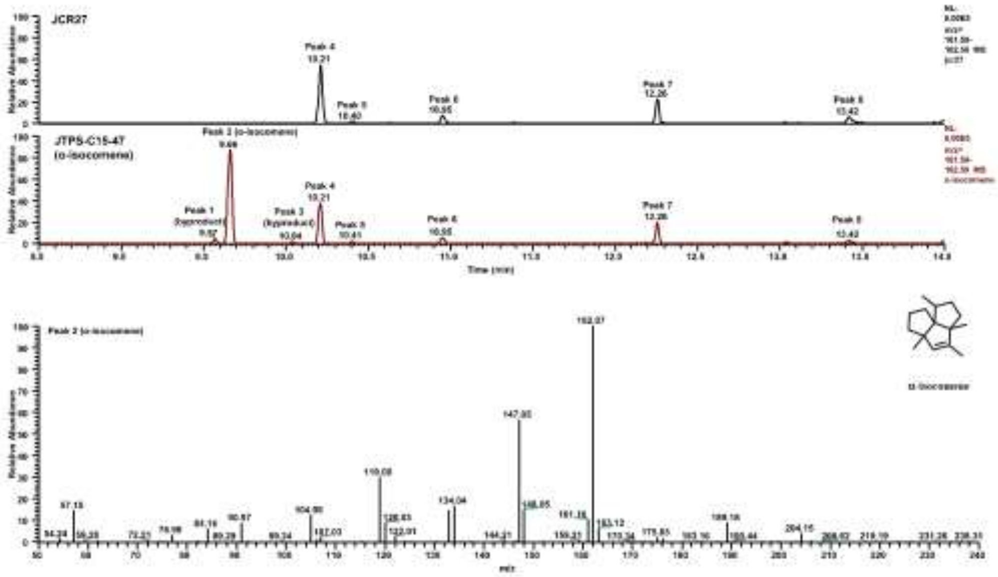

Figure S17 Retention time and fragmentation pattern of biosynthetic α-isocomene (peak 2, RT: 9.66 min), the fermentation broth from strain JCR27 was used as control, except for the target product, there were other products could be synthesized, including the products synthesized by strain JCR27 (peak 4, peak 5, peak 6, peak 7 and peak 8) and the byproducts synthesized by strain JTPS-C15-47 (peak 1 and peak 3).

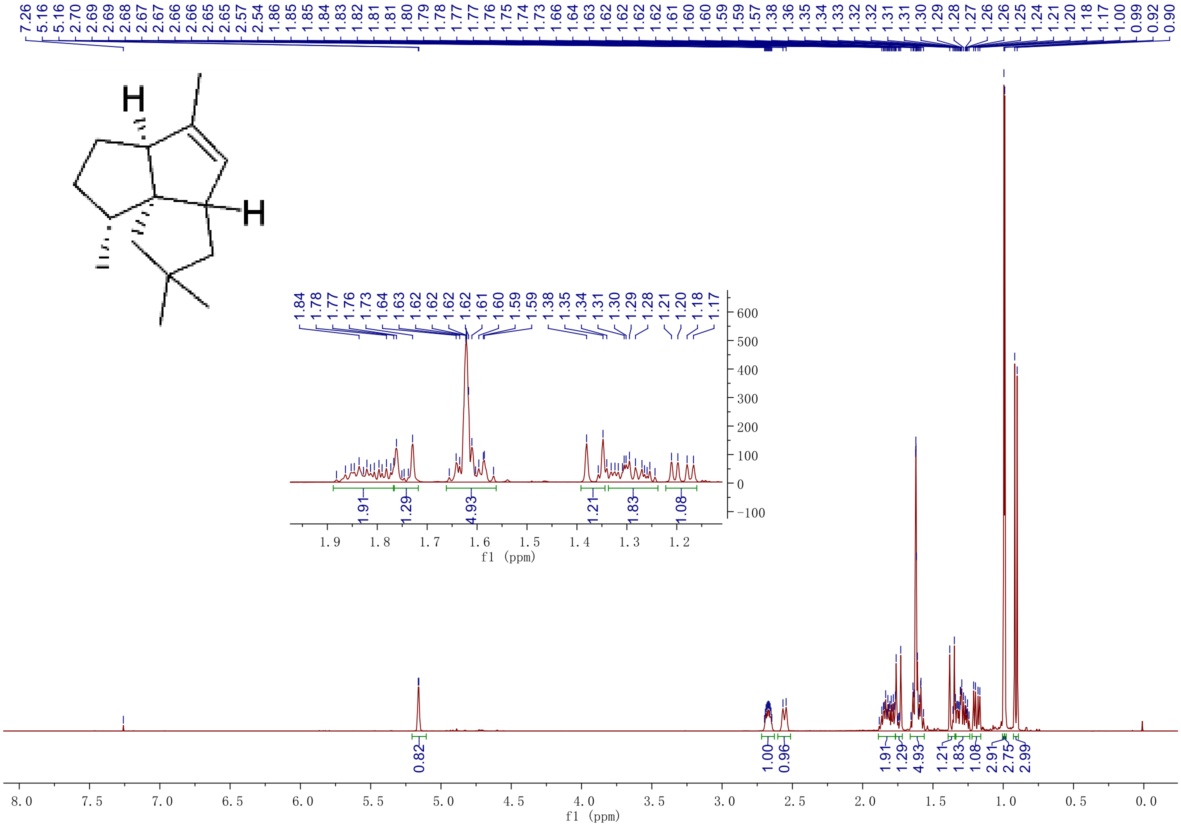

Figure S18. ^{1}H NMR spectrum of biosynthetic pentalanene.

^{1}H NMR (400 MHz, cdcl$_3$) δ 5.16 (d, *J* = 1.5 Hz, 1H), 2.67 (dtd, *J* = 9.2, 4.6, 2.2 Hz, 1H), 2.56 (d, *J* = 9.1 Hz, 1H), 1.95 – 1.77 (m, 2H), 1.77 – 1.72 (m, 1H), 1.67 – 1.56 (m, 5H), 1.39 – 1.35 (m, 1H), 1.34 – 1.24 (m, 2H), 1.19 (dd, *J* = 12.5, 5.1 Hz, 1H), 1.00 (s, 3H), 0.99 (s, 3H), 0.91 (d, *J* = 7.1 Hz, 3H).

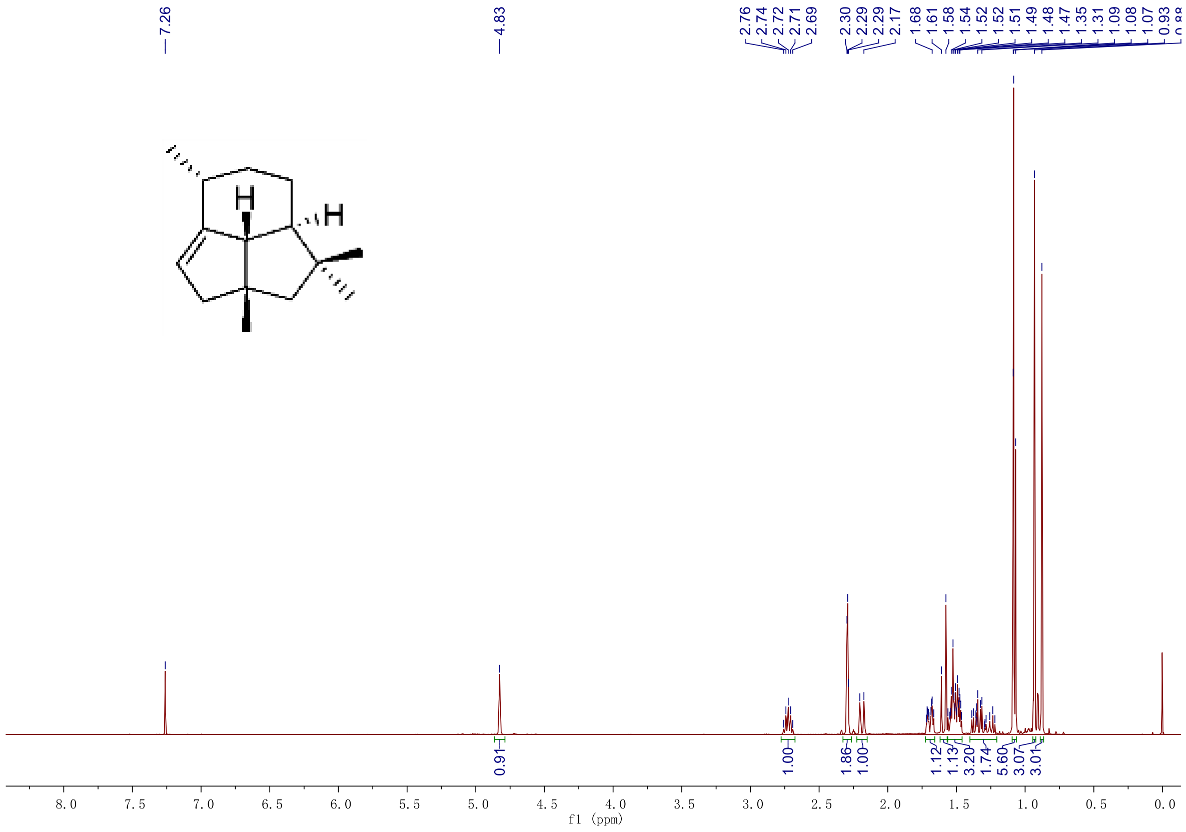

Figure S19. ^{1}H NMR spectrum of biosynthetic presilphiperfol-1-ene.
^{1}H NMR (400 MHz, cdcl$_3$) δ 4.83 (s, 1H), 2.80 – 2.67 (m, 1H), 2.29 (t, *J* = 2.1 Hz, 2H), 2.19 (d, *J* = 11.9 Hz, 1H), 1.73 – 1.65 (m, 1H), 1.59 (d, *J* = 13.1 Hz, 1H), 1.56 – 1.45 (m, 3H), 1.40 – 1.21 (m, 2H), 1.08 (t, *J* = 3.4 Hz, 6H), 0.93 (s, 3H), 0.88 (s, 3H).

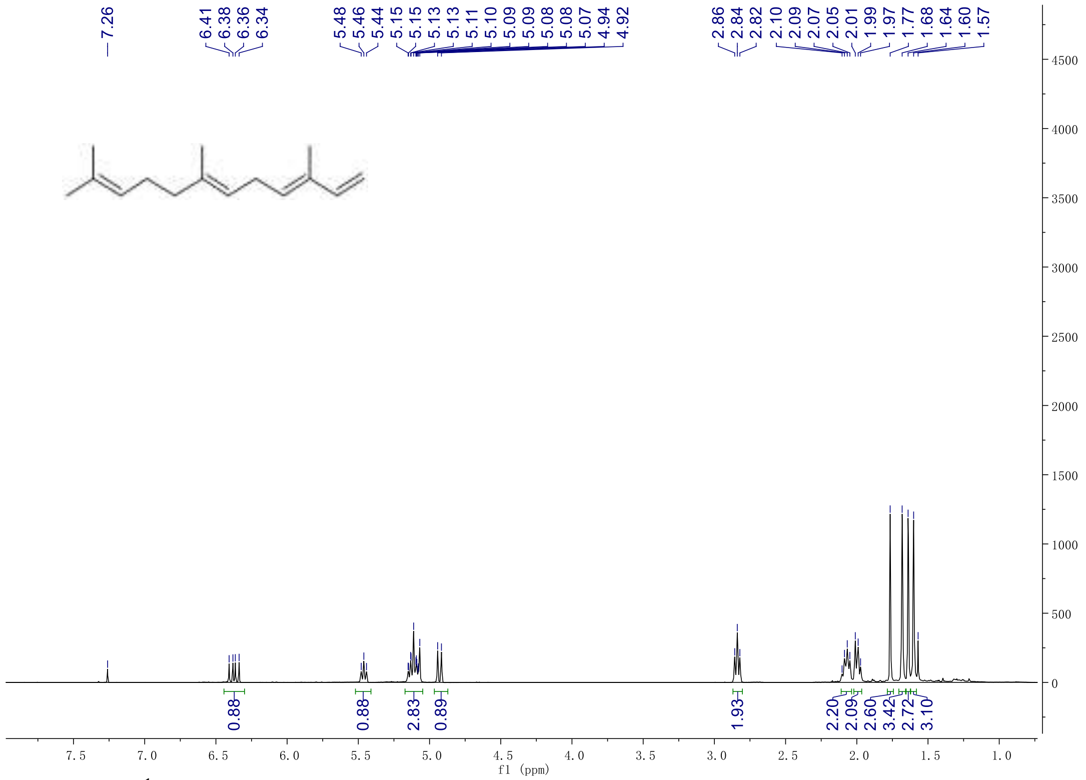

Figure S20. ^{1}H NMR spectrum of biosynthetic α-farnesene

^{1}H NMR (400 MHz, $CDCl_3$) δ 6.37 (dd, *J* = 17.4, 10.7 Hz, 1H), 5.46 (t, *J* = 7.3 Hz, 1H), 5.21 – 5.03 (m, 3H), 4.93 (d, *J* = 10.7 Hz, 1H), 2.84 (t, *J* = 7.2 Hz, 2H), 2.08 (dd, *J* = 14.8, 7.1 Hz, 2H), 2.02 – 1.96 (m, 2H), 1.77 (s, 3H), 1.68 (s, 3H), 1.64 (s, 3H), 1.60 (s, 3H).

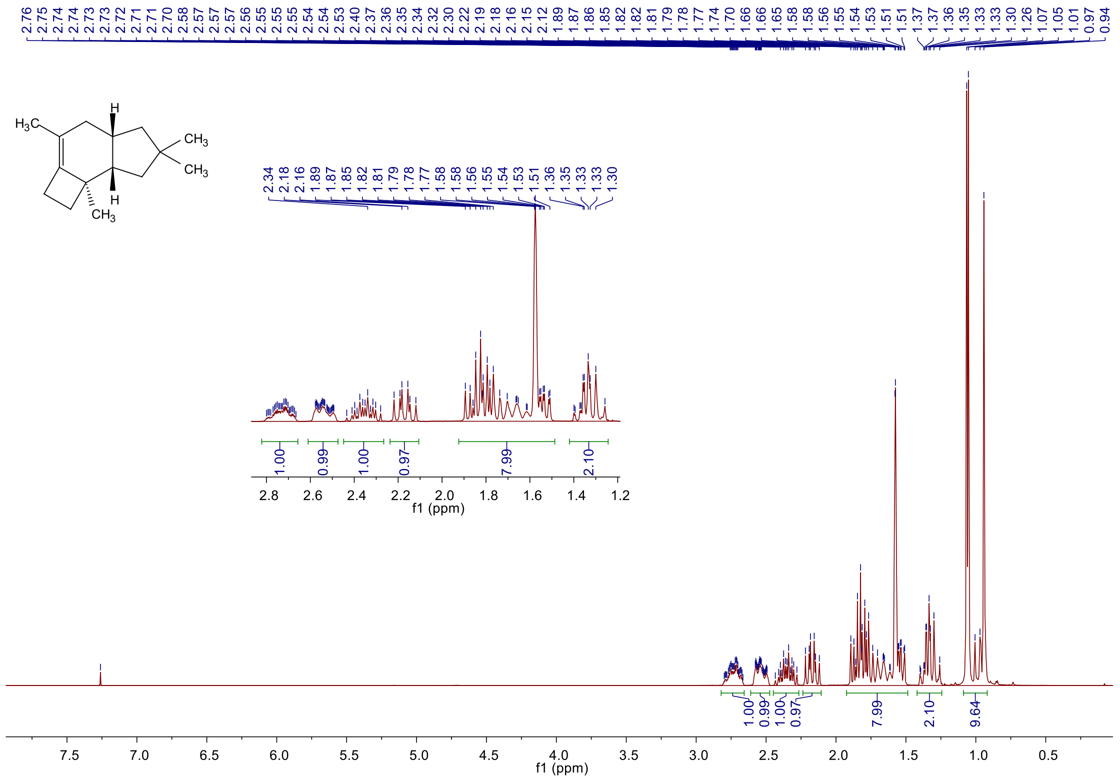

Figure S21. 1H NMR spectrum of biosynthetic protoilludene.

1H NMR (300 MHz, CDCl3) δ 2.84 - 2.66 (m, 1H), 2.61 - 2.48 (m, 1H), 2.45 - 2.27 (m, 1H), 2.23 – 2.10 (m, 1H), 1.91 - 1.48 (m, 8H), 1.42 - 1.24 (m, 2H), 1.09 - 0.92 (m, 10H).

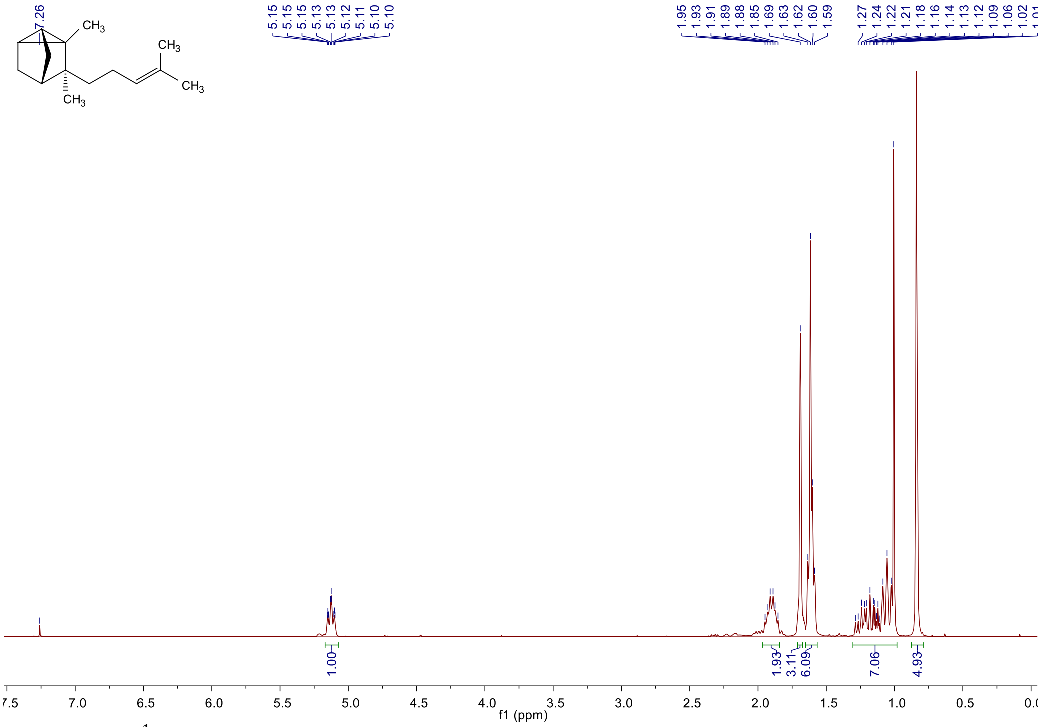

Figure S22. ^{1}H NMR spectrum of biosynthetic α-santalene.

^{1}H NMR (300 MHz, $CDCl_3$) δ 5.16 - 5.08 (m, 1H), 1.90 (dq, J = 10.9 Hz, J = 6.4 Hz, 2H), 1.69 (s, 3H), 1.65 - 1.57 (m, 6H), 1.31 - 0.97 (m, 7H), 0.86 - 0.80 (m, 5H).

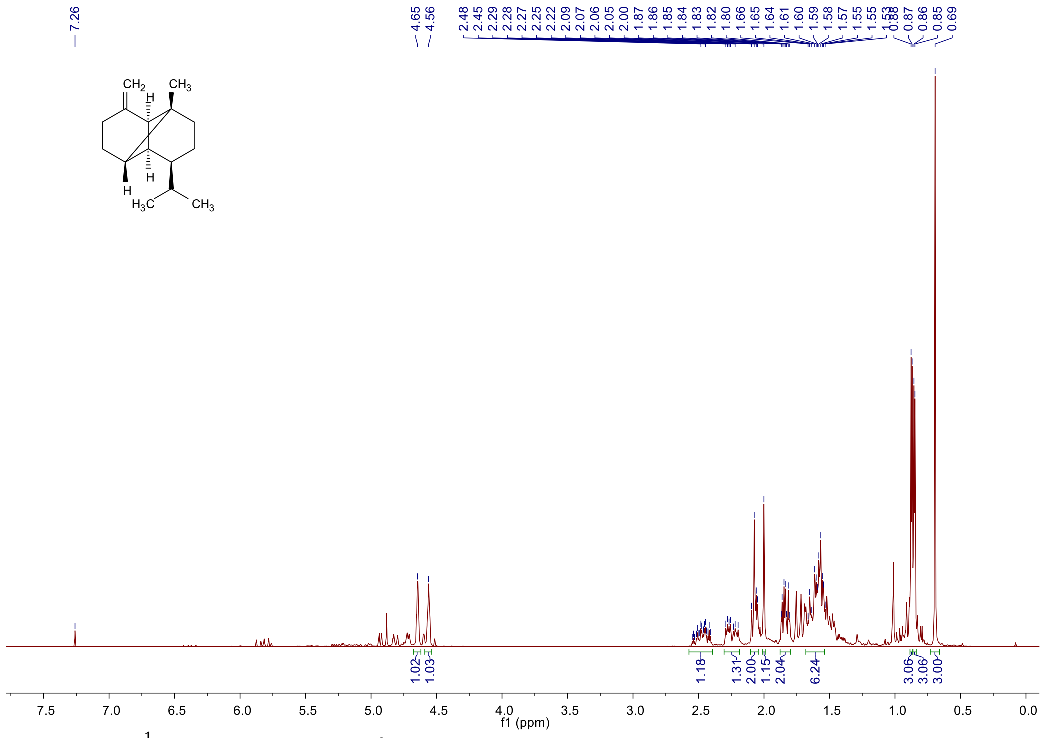

Figure S23. ^{1}H NMR spectrum of biosynthetic β-copaene.

^{1}H NMR (300 MHz, $CDCl_3$) δ 4.66 - 4.62 (m, 1H), 4.58 - 4.53 (m, 1H), 2.56 - 2.39 (m, 1H), 2.33 - 2.18 (m, 1H), 2.12 - 2.05 (m, 2H), 2.00 (s, 1H), 1.89 - 1.80 (m, 2H), 1.72 - 1.48 (m, 6H), 0.87 (d, *J* = 2.4 Hz, 3H), 0.85 (d, *J* = 2.3 Hz, 3H), 0.69 (s, 3H).

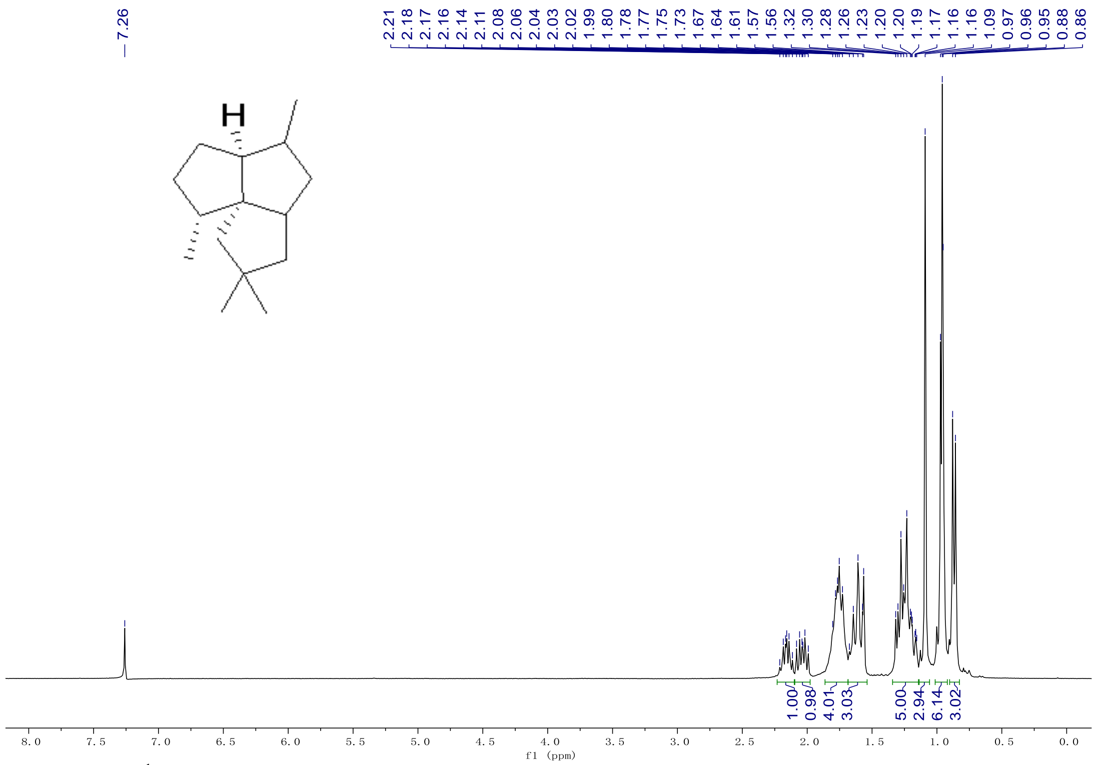

Figure S24. ^{1}H NMR spectrum of pentalenane

^{1}H NMR (300 MHz, $CDCl_3$) δ 2.16 (m, 1H), 2.10 – 1.97 (m, 1H), 1.86 – 1.68 (m, 4H), 1.68 – 1.53 (m, 3H), 1.35 – 1.14 (m, 5H), 1.09 (s, 3H), 0.96 (t, J = 3.1 Hz, 6H), 0.87 (d, J = 6.6 Hz, 3H). MS (m/z) M^+ 206.2.

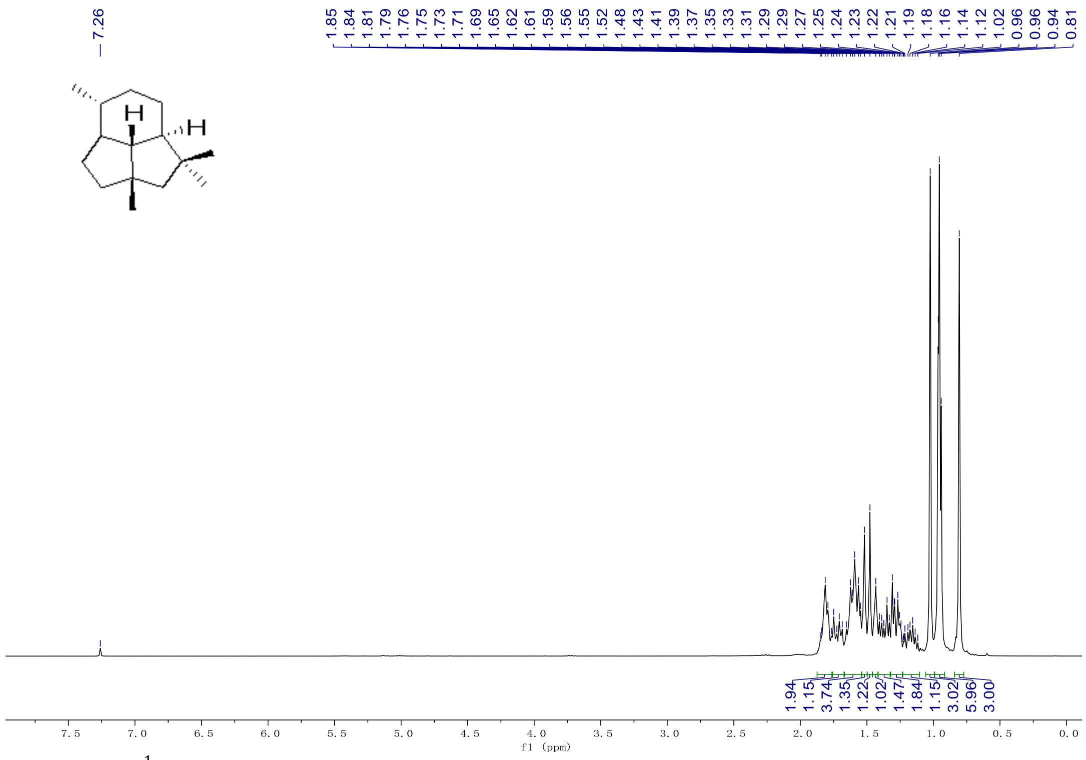

Figure S25. ^{1}H NMR spectrum of presilphiperfol-1-ane

^{1}H NMR (300 MHz, $CDCl_3$) δ 1.82 (m, 2H), 1.77 – 1.67 (m, 1H), 1.60 (m, 4H), 1.52 (s, 1H), 1.48 (s, 1H), 1.43 (s, 1H), 1.41 – 1.32 (m, 1H), 1.26 (m, 2H), 1.23 – 1.11 (m, 1H), 1.02 (s, 3H), 0.95 (t, *J* = 3.4 Hz, 6H), 0.81 (s, 3H). MS (m/z) M^+ 206.2.

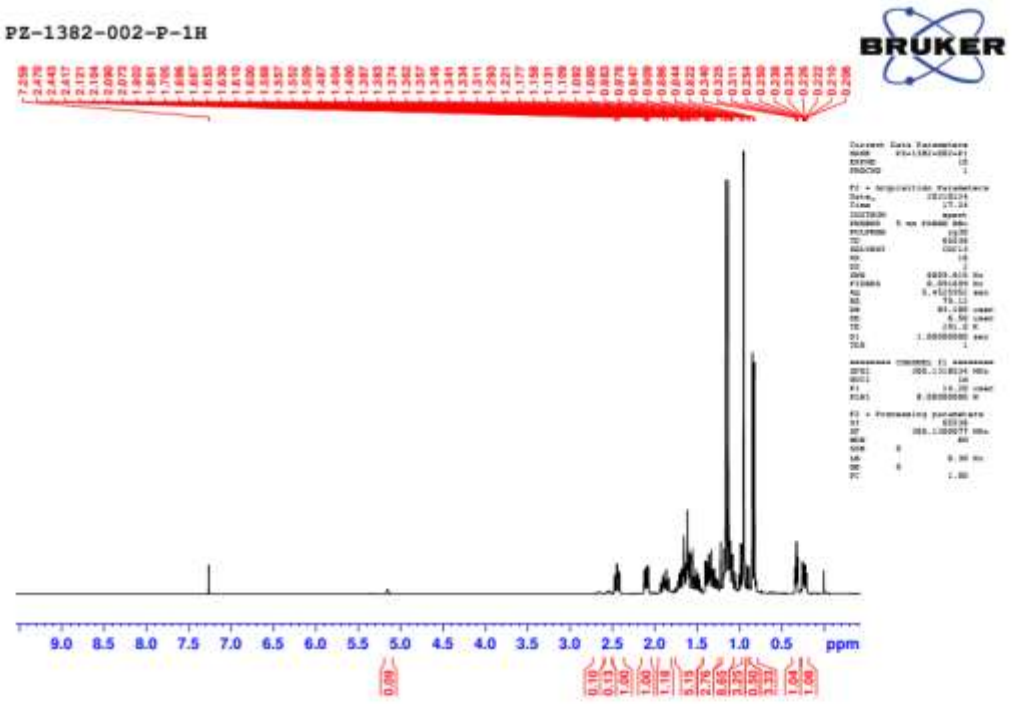

Figure S26. ^{1}H NMR spectrum of cyclopropanation of pentalenene. 1H NMR (300 MHz, CDCl3) δ 2.457 (d, J = 7.8 Hz, 1H), 2.12 (dd, J = 9.0, 5.1 Hz, 1H), 1.92-1.85 (m, 1H), 1.74 – 1.48 (m, 5H), 1.42 – 1.31 (m, 3H), 1.23-1.07 (m, 7H), 1.02-0.90 (m, 5H), 0.85 (d, J = 13.8 Hz, 3H), 0.34 (t, J = 4.5 Hz, 1H), 0.27 – 0.22 (m, 1H).

Table S1 Calculation of important performance parameters of sesquiterpene molecules with different structures

Saturated compound No.	compound	Structure	Volumetric energy density (MJ/L)	Liquid density (kg/L)	Freezing point （℃）	Flash point （℃）	Specific impulse ISP(s)	Combustion heat (MJ/kg)
1	Farnesene	acyclic	34.56	0.783	-60.306	101.608	338.262	44.14
2	Germacrene D	monocyclic	36.79	0.838	-9.066	106.567	338.995	43.9
3	Helminthogermacrene	monocyclic	36.82	0.838	-9.066	106.567	339.148	43.94
4	α-Humulene	monocyclic	36.30	0.826	27.043	104.14	339.188	43.95
5	β-Elemene	monocyclic	35.84	0.818	-64.78	96.555	338.648	43.81
6	(E)- β-Caryophyllene	bicyclic	36.85	0.848	31.902	103.913	338.942	43.46
7	3，7-di-*epi*-trifara-9,14-diene	bicyclic	37.58	0.851	-22.255	98.619	341.687	44.16
8	Acora-3,5-diene	bicyclic	38.10	0.883	-17.185	101.405	337.752	43.15
9	Acoradiene	bicyclic	38.02	0.883	-17.185	101.405	337.375	43.06
10	Aristolochene	bicyclic	37.96	0.883	6.107	103.856	337.107	42.99
11	Asteriscene	bicyclic	36.67	0.848	31.902	103.913	338.083	43.24
12	Bazzanene	bicyclic	37.65	0.868	19.126	98.614	338.587	43.37
13	Bicycloelemene	bicyclic	35.77	0.822	-17.185	95.607	339.136	43.51
14	Bicyclogermacrene	bicyclic	37.01	0.848	31.902	103.913	339.637	43.64
15	Brasila 1,10-diene	bicyclic	36.22	0.843	13.637	102.179	337.016	42.96
16	Brasila 5,6-diene	bicyclic	36.26	0.843	13.637	102.179	337.187	43.01
17	Cumacrene	bicyclic	38.64	0.889	-11.398	103.948	338.967	43.46
18	Dactylene	bicyclic	36.66	0.848	31.902	103.913	338.063	43.23
19	Daucene	bicyclic	38.15	0.883	6.107	103.856	337.93	43.2
20	Drimenene	bicyclic	35.74	0.840	29.073	97.685	337.105	42.55

Saturated compound No.	compound	Structure	Volumetric energy density (MJ/L)	Liquid density (kg/L)	Freezing point （℃）	Flash point （℃）	Specific impulse ISP(s)	Combustion heat (MJ/kg)
21	Epi-zonarene	bicyclic	37.63	0.875	-1.03	106.537	337.188	43.01
22	Eremophilene	bicyclic	38.24	0.883	6.107	103.856	338.377	43.31
23	Fusariumdiene	bicyclic	37.53	0.868	7.179	99.056	338.109	43.24
24	Gleenene	bicyclic	38.00	0.883	-17.185	101.405	337.275	43.03
25	Gorgonene	bicyclic	38.23	0.883	6.107	103.856	338.276	43.29
26	Guaiadiene	bicyclic	37.04	0.859	-2.886	106.282	337.614	43.12
27	Guaie-9,11-diene	bicyclic	37.02	0.859	-2.886	106.282	337.539	43.1
28	Herbertene	bicyclic	37.72	0.874	25.22	101.67	337.781	43.16
29	Himachala-9,11-diene	bicyclic	36.63	0.848	31.902	103.913	337.899	43.19
30	Illudalene	bicyclic	34.22	0.797	7.742	101.176	336.92	42.94
31	Isobazzanene	bicyclic	37.70	0.874	25.22	101.67	337.685	43.14
32	Isochamigrene	bicyclic	37.62	0.870	20.726	99.054	338.104	43.24
33	Isodaucene	bicyclic	38.21	0.883	6.107	103.856	338.195	43.27
34	Macrocarpene	bicyclic	35.44	0.826	27.366	111.558	336.784	42.91
35	Nardosina-7,9,11-triene	bicyclic	38.13	0.883	6.107	103.856	337.862	43.18
36	Neotrifaradiene	bicyclic	36.26	0.840	-25.865	97.252	337.829	43.17
37	Pinguisene	bicyclic	35.66	0.826	-3.626	96.59	337.834	43.17
38	sandvicene	bicyclic	37.10	0.860	9.181	103.167	337.716	43.14
39	Selina-4,11-diene	bicyclic	38.22	0.883	6.107	103.856	338.239	43.28
40	Trichodiene	bicyclic	33.96	0.800	29.819	101.68	336.696	42.45
41	Valencene	bicyclic	37.93	0.883	6.107	103.856	337.005	42.96

Saturated compound No.	compound	Structure	Volumetric energy density (MJ/L)	Liquid density (kg/L)	Freezing point （℃）	Flash point （℃）	Specific impulse ISP(s)	Combustion heat (MJ/kg)
42	Valerene	bicyclic	39.33	0.910	14.583	101.398	337.997	43.22
43	Vetispiradiene	bicyclic	38.14	0.883	-17.185	101.405	337.896	43.19
44	Widdrene	bicyclic	37.85	0.872	39.05	101.445	338.752	43.41
45	α-Bulnesene	bicyclic	37.08	0.859	-2.886	106.282	337.829	43.17
46	α-Chamigrene	bicyclic	37.58	0.870	20.726	99.054	337.916	43.19
47	α-Cuprenene	bicyclic	37.84	0.874	25.22	101.67	338.3	43.29
48	α-Himachalene	bicyclic	36.64	0.848	31.902	103.913	337.975	43.21
49	α-Muurolene	bicyclic	37.01	0.859	-2.886	106.282	337.467	43.08
50	α-Neocallitropsene	bicyclic	38.14	0.883	-17.185	101.405	337.91	43.19
51	α-Selinene	bicyclic	38.01	0.883	6.107	103.856	337.35	43.05
52	β-acoradiene	bicyclic	38.09	0.883	-17.185	101.405	337.711	43.14
53	β-selinene	bicyclic	38.01	0.883	6.107	103.856	337.35	43.05
54	δ-Cadinene	bicyclic	37.05	0.859	-2.886	106.282	337.665	43.13
55	γ-Muurolene	bicyclic	37.05	0.860	-2.89	106.28	337.66	43.13
56	Amorphadiene	bicyclic	36.98	0.860	-2.89	106.28	337.36	43.05
57	4-epi-β-patchoulene	tricyclic	40.09	0.910	43.535	101.151	342.995	44.05
58	African-1-ene	tricyclic	39.01	0.910	43.535	101.151	338.364	42.87
59	Aristolene	tricyclic	39.39	0.910	43.535	101.151	340.032	43.29
60	Aromadendrene	tricyclic	37.87	0.881	36.606	103.592	338.831	42.99
61	Bicycloopposítene	tricyclic	39.61	0.922	11.693	103.505	338.737	42.96
62	Capnellene	tricyclic	38.71	0.910	43.535	101.151	337.06	42.54

Saturated compound No.	compound	Structure	Volumetric energy density (MJ/L)	Liquid density (kg/L)	Freezing point （°C）	Flash point （°C）	Specific impulse ISP(s)	Combustion heat (MJ/kg)
63	Caryolene	tricyclic	41.00	0.955	52.607	104.872	338.605	42.93
64	Clovene	tricyclic	41.69	0.978	42.945	100.049	337.427	42.63
65	Cyclobazzanene	tricyclic	47.00	1.105	21.401	85.672	337.041	42.53
66	Cyclocaryophyllene	tricyclic	39.38	0.910	43.535	101.151	339.966	43.28
67	Duprezianene	tricyclic	38.50	0.903	25.794	98.627	337.44	42.64
68	epi-fusagramineol	tricyclic	40.61	0.929	-0.071	93.474	341.66	43.71
69	epi-isozizaene	tricyclic	38.40	0.903	25.794	98.627	337.018	42.53
70	Helifolene	tricyclic	39.75	0.933	33.237	96.168	337.288	42.6
71	Hirsutene	tricyclic	38.66	0.910	43.535	101.151	336.835	42.48
72	Illudene	tricyclic	38.70	0.903	25.794	98.627	338.341	42.86
73	Isocedrene	tricyclic	38.58	0.903	25.794	98.627	337.789	42.72
74	Isocyclobazzanene	tricyclic	40.16	0.944	50.153	98.677	337.082	42.54
75	Isolactarene	tricyclic	38.57	0.900	40.923	94.817	338.284	42.85
76	Isolongifolene	tricyclic	39.17	0.917	48.843	98.311	337.792	42.72
77	Italicene	tricyclic	38.68	0.903	25.794	98.627	338.198	42.83
78	Kelsoene	tricyclic	39.49	0.922	11.693	103.505	338.216	42.83
79	Khusiene	tricyclic	39.75	0.933	33.237	96.168	337.286	42.6
80	Longibornene	tricyclic	41.80	0.944	50.153	98.677	343.876	44.28
81	Longifolene	tricyclic	38.98	0.910	43.535	101.151	338.264	42.84
82	Maaliene	tricyclic	40.37	0.910	43.535	101.151	344.216	44.36
83	Marasmene	tricyclic	38.49	0.900	40.923	94.817	337.992	42.77

Saturated compound No.	compound	Structure	Volumetric energy density (MJ/L)	Liquid density (kg/L)	Freezing point （℃）	Flash point （℃）	Specific impulse ISP(s)	Combustion heat (MJ/kg)
84	Microbiotene	tricyclic	39.36	0.913	31.767	95.642	339.315	43.11
85	Modephene	tricyclic	39.44	0.926	14.126	93.448	337.264	42.59
86	Neoclovene	tricyclic	39.87	0.933	33.237	96.168	337.818	42.73
87	Panaginsene	tricyclic	38.37	0.903	25.794	98.627	336.863	42.49
88	Panasinsene	tricyclic	41.27	0.933	33.237	96.168	343.712	44.23
89	Pentalenene	tricyclic	38.35	0.903	25.794	98.627	336.788	42.47
90	Presilphiperfol-1-ene	tricyclic	38.98	0.910	43.535	101.151	338.203	42.83
91	Prezizaene	tricyclic	38.53	0.903	25.794	98.627	337.564	42.67
92	Rotundene	tricyclic	40.09	0.940	30.185	105.258	337.491	42.65
93	Sativene	tricyclic	39.32	0.922	11.693	103.505	337.513	42.65
94	Seychellene	tricyclic	39.62	0.929	27.323	99.047	337.49	42.65
95	Silphinene	tricyclic	40.17	0.933	33.237	96.168	339.083	43.05
96	Silphiperfolene	tricyclic	39.09	0.919	7.375	96.476	337.069	42.54
97	Sterpurene	tricyclic	38.88	0.910	43.535	101.151	337.819	42.73
98	Thujopsene	tricyclic	40.09	0.933	33.237	96.168	338.781	42.97
99	Zizaene	tricyclic	40.28	0.912	25.794	98.627	343.471	44.17
100	α-Barbatene	tricyclic	42.04	0.965	34.692	96.559	341.069	43.56
101	α-Cedrene	tricyclic	38.50	0.903	25.794	98.627	337.462	42.64
102	α-Copaene	tricyclic	39.49	0.922	11.693	103.505	338.195	42.83
103	α-isocomene	tricyclic	40.91	0.952	15.781	94.002	338.745	42.97
104	α-santalene	tricyclic	39.02	0.907	-23	98.67	338.87	43

Saturated compound No.	compound	Structure	Volumetric energy density (MJ/L)	Liquid density (kg/L)	Freezing point (℃)	Flash point (℃)	Specific impulse ISP(s)	Combustion heat (MJ/kg)
105	β-Bourbonene	tricyclic	39.43	0.922	11.693	103.505	337.973	42.77
106	β-Cubebene	tricyclic	39.16	0.915	-10.663	100.924	338.099	42.8
107	β-Longipinene	tricyclic	39.05	0.910	43.535	101.151	338.522	42.91
108	β-Terrecyclene	tricyclic	38.60	0.903	25.794	98.627	337.88	42.75
109	Δ^6-Protoilludene	tricyclic	38.96	0.910	43.535	101.151	338.127	42.81
110	Isoishwarene	tricyclic	40.20	0.960	43.7	107	335	41.79
111	myltaylene	tricyclic	39.60	0.930	34.6	103	336	42.44
112	Modhephene	tricyclic	39.30	0.930	15.7	101	336	42.43
113	Prenopsene	tricyclic	38.60	0.910	55.8	108	336	42.39
114	Longiborneol	tricyclic	38.40	0.900	61.1	114	336	42.44
115	Koraiol	tricyclic	37.80	0.890	65.7	116	336	42.51
116	α-gurjunene	tricyclic	37.40	0.880	48.1	111	336	42.46
117	Cycloaromadendrene	tetracyclic	42.18	0.985	37.899	95.667	339.943	42.82
118	Cycloseychellene	tetracyclic	41.53	0.985	37.899	95.667	337.311	42.16
119	Ishwarene	tetracyclic	45.50	1.071	31.536	97.502	338.593	42.48
120	Longicyclene	tetracyclic	42.66	1.003	54.317	98.313	338.813	42.53
121	cyclomytaylene	tetracyclic	42.90	1.030	46.1	100	335	41.77
122	Anastreptene	tetracyclic	41.10	0.990	37.8	103	335	41.71
※	JP-10	tricyclic	39.60	0.940	<-79	54.4	300.3	42.127

Table S2 Calculation of important performance parameters of bicyclic sesquiterpene molecules (ranking of volumetric energy density)

Saturated compound No.	compound	Structure	Volumetric energy density (MJ/L)	Liquid density (kg/L)	Freezing point	Flash point （℃）	Specific impulse ISP(s)	Combustion heat (MJ/kg)
1	Valerene	bicyclic	39.33	0.91	14.583	101.398	337.997	43.22
2	Cumacrene	bicyclic	38.64	0.889	-11.398	103.948	338.967	43.46
3	Eremophilene	bicyclic	38.24	0.883	6.107	103.856	338.377	43.31
4	Gorgonene	bicyclic	38.23	0.883	6.107	103.856	338.276	43.29
5	Selina-4,11-diene	bicyclic	38.22	0.883	6.107	103.856	338.239	43.28
6	Isodaucene	bicyclic	38.21	0.883	6.107	103.856	338.195	43.27
7	Daucene	bicyclic	38.15	0.883	6.107	103.856	337.93	43.2
8	Vetispiradiene	bicyclic	38.14	0.883	-17.185	101.405	337.896	43.19
9	α-Neocallitropsene	bicyclic	38.14	0.883	-17.185	101.405	337.91	43.19
10	Nardosina-7,9,11-triene	bicyclic	38.13	0.883	6.107	103.856	337.862	43.18
11	Acora-3,5-diene	bicyclic	38.1	0.883	-17.185	101.405	337.752	43.15
12	β-acoradiene	bicyclic	38.09	0.883	-17.185	101.405	337.711	43.14
13	Acoradiene	bicyclic	38.02	0.883	-17.185	101.405	337.375	43.06
14	α-Selinene	bicyclic	38.01	0.883	6.107	103.856	337.35	43.05
15	β-selinene	bicyclic	38.01	0.883	6.107	103.856	337.35	43.05
16	Gleenene	bicyclic	38	0.883	-17.185	101.405	337.275	43.03
17	Aristolochene	bicyclic	37.96	0.883	6.107	103.856	337.107	42.99
18	Valencene	bicyclic	37.93	0.883	6.107	103.856	337.005	42.96
19	Widdrene	bicyclic	37.85	0.872	39.05	101.445	338.752	43.41
20	α-Cuprenene	bicyclic	37.84	0.874	25.22	101.67	338.3	43.29

Saturated compound No.	compound	Structure	Volumetric energy density (MJ/L)	Liquid density (kg/L)	Freezing point	Flash point （℃）	Specific impulse ISP(s)	Combustion heat (MJ/kg)
21	Herbertene	bicyclic	37.72	0.874	25.22	101.67	337.781	43.16
22	Isobazzanene	bicyclic	37.7	0.874	25.22	101.67	337.685	43.14
23	Bazzanene	bicyclic	37.65	0.868	19.126	98.614	338.587	43.37
24	Epi-zonarene	bicyclic	37.63	0.875	-1.03	106.537	337.188	43.01
25	Isochamigrene	bicyclic	37.62	0.87	20.726	99.054	338.104	43.24
26	3，7-di-*epi*-trifara-9,14-diene	bicyclic	37.58	0.851	-22.255	98.619	341.687	44.16
27	α-Chamigrene	bicyclic	37.58	0.87	20.726	99.054	337.916	43.19
28	Fusariumdiene	bicyclic	37.53	0.868	7.179	99.056	338.109	43.24
29	sandvicene	bicyclic	37.1	0.86	9.181	103.167	337.716	43.14
30	α-Bulnesene	bicyclic	37.08	0.859	-2.886	106.282	337.829	43.17
31	δ-Cadinene	bicyclic	37.05	0.859	-2.886	106.282	337.665	43.13
32	γ-Muurolene	bicyclic	37.05	0.86	-2.89	106.28	337.66	43.13
33	Guaiadiene	bicyclic	37.04	0.859	-2.886	106.282	337.614	43.12
34	Guaie-9,11-diene	bicyclic	37.02	0.859	-2.886	106.282	337.539	43.1
35	Bicyclogermacrene	bicyclic	37.01	0.848	31.902	103.913	339.637	43.64
36	α-Muurolene	bicyclic	37.01	0.859	-2.886	106.282	337.467	43.08
37	Amorphadiene	bicyclic	36.98	0.86	-2.89	106.28	337.36	43.05
38	(E)- β-Caryophyllene	bicyclic	36.85	0.848	31.902	103.913	338.942	43.46
39	Asteriscene	bicyclic	36.67	0.848	31.902	103.913	338.083	43.24

Saturated compound No.	compound	Structure	Volumetric energy density (MJ/L)	Liquid density (kg/L)	Freezing point	Flash point（℃）	Specific impulse ISP(s)	Combustion heat (MJ/kg)
40	Dactylene	bicyclic	36.66	0.848	31.902	103.913	338.063	43.23
41	α-Himachalene	bicyclic	36.64	0.848	31.902	103.913	337.975	43.21
42	Himachala-9,11-diene	bicyclic	36.63	0.848	31.902	103.913	337.899	43.19
43	Brasila 5,6-diene	bicyclic	36.26	0.843	13.637	102.179	337.187	43.01
44	Neotrifaradiene	bicyclic	36.26	0.84	-25.865	97.252	337.829	43.17
45	Brasila 1,10-diene	bicyclic	36.22	0.843	13.637	102.179	337.016	42.96
46	Bicycloelemene	bicyclic	35.77	0.822	-17.185	95.607	339.136	43.51
47	Drimenene	bicyclic	35.74	0.84	29.073	97.685	337.105	42.55
48	Pinguisene	bicyclic	35.66	0.826	-3.626	96.59	337.834	43.17
49	Macrocarpene	bicyclic	35.44	0.826	27.366	111.558	336.784	42.91
50	Illudalene	bicyclic	34.22	0.797	7.742	101.176	336.92	42.94
51	Trichodiene	bicyclic	33.96	0.8	29.819	101.68	336.696	42.45

Table S3 Calculation of important performance parameters of bicyclic sesquiterpene molecules (ranking of flash point)

Saturated compound No.	compound	Structure	Volumetric energy density (MJ/L)	Liquid density (kg/L)	Freezing point	Flash point （℃）	Specific impulse ISP(s)	Combustion heat (MJ/kg)
1	Macrocarpene	bicyclic	35.44	0.826	27.366	111.558	336.784	42.91
2	Epi-zonarene	bicyclic	37.63	0.875	-1.03	106.537	337.188	43.01
3	α-Bulnesene	bicyclic	37.08	0.859	-2.886	106.282	337.829	43.17
4	δ-Cadinene	bicyclic	37.05	0.859	-2.886	106.282	337.665	43.13
5	Guaiadiene	bicyclic	37.04	0.859	-2.886	106.282	337.614	43.12
6	Guaie-9,11-diene	bicyclic	37.02	0.859	-2.886	106.282	337.539	43.1
7	α-Muurolene	bicyclic	37.01	0.859	-2.886	106.282	337.467	43.08
8	γ-Muurolene	bicyclic	37.05	0.86	-2.89	106.28	337.66	43.13
9	Amorphadiene	bicyclic	36.98	0.86	-2.89	106.28	337.36	43.05
10	Cumacrene	bicyclic	38.64	0.889	-11.398	103.948	338.967	43.46
11	Bicyclogermacrene	bicyclic	37.01	0.848	31.902	103.913	339.637	43.64
12	(E)- β-Caryophyllene	bicyclic	36.85	0.848	31.902	103.913	338.942	43.46
13	Asteriscene	bicyclic	36.67	0.848	31.902	103.913	338.083	43.24
14	Dactylene	bicyclic	36.66	0.848	31.902	103.913	338.063	43.23
15	α-Himachalene	bicyclic	36.64	0.848	31.902	103.913	337.975	43.21
16	Himachala-9,11-diene	bicyclic	36.63	0.848	31.902	103.913	337.899	43.19
17	Eremophilene	bicyclic	38.24	0.883	6.107	103.856	338.377	43.31
18	Gorgonene	bicyclic	38.23	0.883	6.107	103.856	338.276	43.29

Saturated compound No.	compound	Structure	Volumetric energy density (MJ/L)	Liquid density (kg/L)	Freezing point	Flash point （℃）	Specific impulse ISP(s)	Combustion heat (MJ/kg)
19	Selina-4,11-diene	bicyclic	38.22	0.883	6.107	103.856	338.239	43.28
20	Isodaucene	bicyclic	38.21	0.883	6.107	103.856	338.195	43.27
21	Daucene	bicyclic	38.15	0.883	6.107	103.856	337.93	43.2
22	Nardosina-7,9,11-triene	bicyclic	38.13	0.883	6.107	103.856	337.862	43.18
23	α-Selinene	bicyclic	38.01	0.883	6.107	103.856	337.35	43.05
24	β-selinene	bicyclic	38.01	0.883	6.107	103.856	337.35	43.05
25	Aristolochene	bicyclic	37.96	0.883	6.107	103.856	337.107	42.99
26	Valencene	bicyclic	37.93	0.883	6.107	103.856	337.005	42.96
27	sandvicene	bicyclic	37.1	0.86	9.181	103.167	337.716	43.14
28	Brasila 5,6-diene	bicyclic	36.26	0.843	13.637	102.179	337.187	43.01
29	Brasila 1,10-diene	bicyclic	36.22	0.843	13.637	102.179	337.016	42.96
30	Trichodiene	bicyclic	33.96	0.8	29.819	101.68	336.696	42.45
31	α-Cuprenene	bicyclic	37.84	0.874	25.22	101.67	338.3	43.29
32	Herbertene	bicyclic	37.72	0.874	25.22	101.67	337.781	43.16
33	Isobazzanene	bicyclic	37.7	0.874	25.22	101.67	337.685	43.14
34	Widdrene	bicyclic	37.85	0.872	39.05	101.445	338.752	43.41
35	Vetispiradiene	bicyclic	38.14	0.883	-17.185	101.405	337.896	43.19
36	α-Neocallitropsene	bicyclic	38.14	0.883	-17.185	101.405	337.91	43.19
37	Acora-3,5-diene	bicyclic	38.1	0.883	-17.185	101.405	337.752	43.15
38	β-acoradiene	bicyclic	38.09	0.883	-17.185	101.405	337.711	43.14

Saturated compound No.	compound	Structure	Volumetric energy density (MJ/L)	Liquid density (kg/L)	Freezing point	Flash point （℃）	Specific impulse ISP(s)	Combustion heat (MJ/kg)
39	Acoradiene	bicyclic	38.02	0.883	-17.185	101.405	337.375	43.06
40	Gleenene	bicyclic	38	0.883	-17.185	101.405	337.275	43.03
41	Valerene	bicyclic	39.33	0.91	14.583	101.398	337.997	43.22
42	Illudalene	bicyclic	34.22	0.797	7.742	101.176	336.92	42.94
43	Fusariumdiene	bicyclic	37.53	0.868	7.179	99.056	338.109	43.24
44	Isochamigrene	bicyclic	37.62	0.87	20.726	99.054	338.104	43.24
45	α-Chamigrene	bicyclic	37.58	0.87	20.726	99.054	337.916	43.19
46	3，7-di-*epi*-trifara-9,14-diene	bicyclic	37.58	0.851	-22.255	98.619	341.687	44.16
47	Bazzanene	bicyclic	37.65	0.868	19.126	98.614	338.587	43.37
48	Drimenene	bicyclic	35.74	0.84	29.073	97.685	337.105	42.55
49	Neotrifaradiene	bicyclic	36.26	0.84	-25.865	97.252	337.829	43.17
50	Pinguisene	bicyclic	35.66	0.826	-3.626	96.59	337.834	43.17
51	Bicycloelemene	bicyclic	35.77	0.822	-17.185	95.607	339.136	43.51

Table S4 Calculation of important performance parameters of tricyclic sesquiterpene molecules (ranking of volumetric energy density)

Saturated compound No.	compound	Structure	Volumetric energy density (MJ/L)	Liquid density (kg/L)	Freezing point	Flash point （℃）	Specific impulse ISP(s)	Combustion heat (MJ/kg)
1	Cyclobazzanene	tricyclic	47	1.105	21.401	85.672	337.041	42.53
2	α-Barbatene	tricyclic	42.04	0.965	34.692	96.559	341.069	43.56
3	Longibornene	tricyclic	41.8	0.944	50.153	98.677	343.876	44.28
4	Clovene	tricyclic	41.69	0.978	42.945	100.049	337.427	42.63
5	Panasinsene	tricyclic	41.27	0.933	33.237	96.168	343.712	44.23
6	Caryolene	tricyclic	41	0.955	52.607	104.872	338.605	42.93
7	α-isocomene	tricyclic	40.91	0.952	15.781	94.002	338.745	42.97
8	epi-fusagramineol	tricyclic	40.61	0.929	-0.071	93.474	341.66	43.71
9	Maaliene	tricyclic	40.37	0.91	43.535	101.151	344.216	44.36
10	Zizaene	tricyclic	40.28	0.912	25.794	98.627	343.471	44.17
11	Isoishwarene	tricyclic	40.2	0.96	43.7	107	335	41.79
12	Silphinene	tricyclic	40.17	0.933	33.237	96.168	339.083	43.05
13	Isocyclobazzanene	tricyclic	40.16	0.944	50.153	98.677	337.082	42.54
14	4-epi-β-patchoulene	tricyclic	40.09	0.91	43.535	101.151	342.995	44.05
15	Rotundene	tricyclic	40.09	0.94	30.185	105.258	337.491	42.65
16	Thujopsene	tricyclic	40.09	0.933	33.237	96.168	338.781	42.97
17	Neoclovene	tricyclic	39.87	0.933	33.237	96.168	337.818	42.73
18	Helifolene	tricyclic	39.75	0.933	33.237	96.168	337.288	42.6
19	Khusiene	tricyclic	39.75	0.933	33.237	96.168	337.286	42.6
20	Seychellene	tricyclic	39.62	0.929	27.323	99.047	337.49	42.65

Saturated compound No.	compound	Structure	Volumetric energy density (MJ/L)	Liquid density (kg/L)	Freezing point	Flash point （℃）	Specific impulse ISP(s)	Combustion heat (MJ/kg)
21	BicycloopposPositene	tricyclic	39.61	0.922	11.693	103.505	338.737	42.96
22	myltaylene	tricyclic	39.6	0.93	34.6	103	336	42.44
23	Kelsoene	tricyclic	39.49	0.922	11.693	103.505	338.216	42.83
24	α-Copaene	tricyclic	39.49	0.922	11.693	103.505	338.195	42.83
25	Modephene	tricyclic	39.44	0.926	14.126	93.448	337.264	42.59
26	β-Bourbonene	tricyclic	39.43	0.922	11.693	103.505	337.973	42.77
27	Aristolene	tricyclic	39.39	0.91	43.535	101.151	340.032	43.29
28	Cyclocaryophyllene	tricyclic	39.38	0.91	43.535	101.151	339.966	43.28
29	Microbiotene	tricyclic	39.36	0.913	31.767	95.642	339.315	43.11
30	Sativene	tricyclic	39.32	0.922	11.693	103.505	337.513	42.65
31	Modhephene	tricyclic	39.3	0.93	15.7	101	336	42.43
32	Isolongifolene	tricyclic	39.17	0.917	48.843	98.311	337.792	42.72
33	β-Cubebene	tricyclic	39.16	0.915	-10.663	100.924	338.099	42.8
34	Silphiperfolene	tricyclic	39.09	0.919	7.375	96.476	337.069	42.54
35	β-Longipinene	tricyclic	39.05	0.91	43.535	101.151	338.522	42.91
36	α-santalene	tricyclic	39.02	0.907	-23	98.67	338.87	43
37	African-1-ene	tricyclic	39.01	0.91	43.535	101.151	338.364	42.87
38	Longifolene	tricyclic	38.98	0.91	43.535	101.151	338.264	42.84
39	Presilphiperfol-1-ene	tricyclic	38.98	0.91	43.535	101.151	338.203	42.83
40	Δ^6-Protoilludene	tricyclic	38.96	0.91	43.535	101.151	338.127	42.81

Saturated compound No.	compound	Structure	Volumetric energy density (MJ/L)	Liquid density (kg/L)	Freezing point	Flash point （℃）	Specific impulse ISP(s)	Combustion heat (MJ/kg)
41	Sterpurene	tricyclic	38.88	0.91	43.535	101.151	337.819	42.73
42	Capnellene	tricyclic	38.71	0.91	43.535	101.151	337.06	42.54
43	Illudene	tricyclic	38.7	0.903	25.794	98.627	338.341	42.86
44	Italicene	tricyclic	38.68	0.903	25.794	98.627	338.198	42.83
45	Hirsutene	tricyclic	38.66	0.91	43.535	101.151	336.835	42.48
46	β-Terrecyclene	tricyclic	38.6	0.903	25.794	98.627	337.88	42.75
47	Prenopsene	tricyclic	38.6	0.91	55.8	108	336	42.39
48	Isocedrene	tricyclic	38.58	0.903	25.794	98.627	337.789	42.72
49	Isolactarene	tricyclic	38.57	0.9	40.923	94.817	338.284	42.85
50	Prezizaene	tricyclic	38.53	0.903	25.794	98.627	337.564	42.67
51	Duprezianene	tricyclic	38.5	0.903	25.794	98.627	337.44	42.64
52	α-Cedrene	tricyclic	38.5	0.903	25.794	98.627	337.462	42.64
53	Marasmene	tricyclic	38.49	0.9	40.923	94.817	337.992	42.77
54	epi-isozizaene	tricyclic	38.4	0.903	25.794	98.627	337.018	42.53
55	Longiborneol	tricyclic	38.4	0.9	61.1	114	336	42.44
56	Panaginsene	tricyclic	38.37	0.903	25.794	98.627	336.863	42.49
57	Pentalenene	tricyclic	38.35	0.903	25.794	98.627	336.788	42.47
58	Aromadendrene	tricyclic	37.87	0.881	36.606	103.592	338.831	42.99
59	Koraiol	tricyclic	37.8	0.89	65.7	116	336	42.51
60	α-gurjunene	tricyclic	37.4	0.88	48.1	111	336	42.46

Table S5 Calculation of important performance parameters of tricyclic sesquiterpene molecules (ranking of freezing point)

Saturated compound No.	compound	Structure	Volumetric energy density (MJ/L)	Liquid density (kg/L)	Freezing point	Flash point （℃）	Specific impulse ISP(s)	Combustion heat (MJ/kg)
1	α-santalene	tricyclic	39.02	0.907	-23	98.67	338.87	43
2	β-Cubebene	tricyclic	39.16	0.915	-10.663	100.924	338.099	42.8
3	epi-fusagramineol	tricyclic	40.61	0.929	-0.071	93.474	341.66	43.71
4	Silphiperfolene	tricyclic	39.09	0.919	7.375	96.476	337.069	42.54
5	Bicycloopposítene	tricyclic	39.61	0.922	11.693	103.505	338.737	42.96
6	Kelsoene	tricyclic	39.49	0.922	11.693	103.505	338.216	42.83
7	α-Copaene	tricyclic	39.49	0.922	11.693	103.505	338.195	42.83
8	β-Bourbonene	tricyclic	39.43	0.922	11.693	103.505	337.973	42.77
9	Sativene	tricyclic	39.32	0.922	11.693	103.505	337.513	42.65
10	Modephene	tricyclic	39.44	0.926	14.126	93.448	337.264	42.59
11	Modhephene	tricyclic	39.3	0.93	15.7	101	336	42.43
12	α-isocomene	tricyclic	40.91	0.952	15.781	94.002	338.745	42.97
13	Cyclobazzanene	tricyclic	47	1.105	21.401	85.672	337.041	42.53
14	Zizaene	tricyclic	40.28	0.912	25.794	98.627	343.471	44.17
15	Illudene	tricyclic	38.7	0.903	25.794	98.627	338.341	42.86
16	Italicene	tricyclic	38.68	0.903	25.794	98.627	338.198	42.83
17	β-Terrecyclene	tricyclic	38.6	0.903	25.794	98.627	337.88	42.75
18	Isocedrene	tricyclic	38.58	0.903	25.794	98.627	337.789	42.72
19	Prezizaene	tricyclic	38.53	0.903	25.794	98.627	337.564	42.67
20	Duprezianene	tricyclic	38.5	0.903	25.794	98.627	337.44	42.64
21	α-Cedrene	tricyclic	38.5	0.903	25.794	98.627	337.462	42.64
22	epi-isozizaene	tricyclic	38.4	0.903	25.794	98.627	337.018	42.53
23	Panaginsene	tricyclic	38.37	0.903	25.794	98.627	336.863	42.49

24	Pentalenene	tricyclic	38.35	0.903	25.794	98.627	336.788	42.47
25	Seychellene	tricyclic	39.62	0.929	27.323	99.047	337.49	42.65
26	Rotundene	tricyclic	40.09	0.94	30.185	105.258	337.491	42.65
27	Microbiotene	tricyclic	39.36	0.913	31.767	95.642	339.315	43.11
28	Panasinsene	tricyclic	41.27	0.933	33.237	96.168	343.712	44.23
29	Silphinene	tricyclic	40.17	0.933	33.237	96.168	339.083	43.05
30	Thujopsene	tricyclic	40.09	0.933	33.237	96.168	338.781	42.97
31	Neoclovene	tricyclic	39.87	0.933	33.237	96.168	337.818	42.73
32	Helifolene	tricyclic	39.75	0.933	33.237	96.168	337.288	42.6
33	Khusiene	tricyclic	39.75	0.933	33.237	96.168	337.286	42.6
34	myltaylene	tricyclic	39.6	0.93	34.6	103	336	42.44
35	α-Barbatene	tricyclic	42.04	0.965	34.692	96.559	341.069	43.56
36	Aromadendrene	tricyclic	37.87	0.881	36.606	103.592	338.831	42.99
37	Isolactarene	tricyclic	38.57	0.9	40.923	94.817	338.284	42.85
38	Marasmene	tricyclic	38.49	0.9	40.923	94.817	337.992	42.77
39	Clovene	tricyclic	41.69	0.978	42.945	100.049	337.427	42.63
40	Maaliene	tricyclic	40.37	0.91	43.535	101.151	344.216	44.36
41	4-epi-*b*-patchoulene	tricyclic	40.09	0.91	43.535	101.151	342.995	44.05
42	Aristolene	tricyclic	39.39	0.91	43.535	101.151	340.032	43.29
43	Cyclocaryophyllene	tricyclic	39.38	0.91	43.535	101.151	339.966	43.28
44	β-Longipinene	tricyclic	39.05	0.91	43.535	101.151	338.522	42.91
45	African-1-ene	tricyclic	39.01	0.91	43.535	101.151	338.364	42.87
46	Longifolene	tricyclic	38.98	0.91	43.535	101.151	338.264	42.84
47	Presilphiperfol-1-ene	tricyclic	38.98	0.91	43.535	101.151	338.203	42.83

48	Δ*6*-Protoilludene	tricyclic	38.96	0.91	43.535	101.151	338.127	42.81
49	Sterpurene	tricyclic	38.88	0.91	43.535	101.151	337.819	42.73
50	Capnellene	tricyclic	38.71	0.91	43.535	101.151	337.06	42.54
51	Hirsutene	tricyclic	38.66	0.91	43.535	101.151	336.835	42.48
52	Isoishwarene	tricyclic	40.2	0.96	43.7	107	335	41.79
53	α-gurjunene	tricyclic	37.4	0.88	48.1	111	336	42.46
54	Isolongifolene	tricyclic	39.17	0.917	48.843	98.311	337.792	42.72
55	Longibornene	tricyclic	41.8	0.944	50.153	98.677	343.876	44.28
56	Isocyclobazzanene	tricyclic	40.16	0.944	50.153	98.677	337.082	42.54
57	Caryolene	tricyclic	41	0.955	52.607	104.872	338.605	42.93
58	Prenopsene	tricyclic	38.6	0.91	55.8	108	336	42.39
59	Longiborneol	tricyclic	38.4	0.9	61.1	114	336	42.44
60	Koraiol	tricyclic	37.8	0.89	65.7	116	336	42.51

Table S6 Calculation of important performance parameters of tricyclic sesquiterpene molecules (ranking of specific impulse)

Saturated compound No.	compound	Structure	Volumetric energy density (MJ/L)	Liquid density (kg/L)	Freezing point	Flash point （℃）	Specific impulse ISP(s)	Combustion heat (MJ/kg)
1	Maaliene	tricyclic	40.37	0.91	43.535	101.151	344.216	44.36
2	Longibornene	tricyclic	41.8	0.944	50.153	98.677	343.876	44.28
3	Panasinsene	tricyclic	41.27	0.933	33.237	96.168	343.712	44.23
4	Zizaene	tricyclic	40.28	0.912	25.794	98.627	343.471	44.17
5	4-epi-β-patchoulene	tricyclic	40.09	0.91	43.535	101.151	342.995	44.05
6	epi-fusagramineol	tricyclic	40.61	0.929	-0.071	93.474	341.66	43.71

Saturated compound No.	compound	Structure	Volumetric energy density (MJ/L)	Liquid density (kg/L)	Freezing point	Flash point （℃）	Specific impulse ISP(s)	Combustion heat (MJ/kg)
7	α-Barbatene	tricyclic	42.04	0.965	34.692	96.559	341.069	43.56
8	Aristolene	tricyclic	39.39	0.91	43.535	101.151	340.032	43.29
9	Cyclocaryophyllene	tricyclic	39.38	0.91	43.535	101.151	339.966	43.28
10	Microbiotene	tricyclic	39.36	0.913	31.767	95.642	339.315	43.11
11	Silphinene	tricyclic	40.17	0.933	33.237	96.168	339.083	43.05
12	α-santalene	tricyclic	39.02	0.907	-23	98.67	338.87	43
13	Aromadendrene	tricyclic	37.87	0.881	36.606	103.592	338.831	42.99
14	Thujopsene	tricyclic	40.09	0.933	33.237	96.168	338.781	42.97
15	α-isocomene	tricyclic	40.91	0.952	15.781	94.002	338.745	42.97
16	Bicycloopposítene	tricyclic	39.61	0.922	11.693	103.505	338.737	42.96
17	Caryolene	tricyclic	41	0.955	52.607	104.872	338.605	42.93
18	β-Longipinene	tricyclic	39.05	0.91	43.535	101.151	338.522	42.91
19	African-1-ene	tricyclic	39.01	0.91	43.535	101.151	338.364	42.87
20	Illudene	tricyclic	38.7	0.903	25.794	98.627	338.341	42.86
21	Isolactarene	tricyclic	38.57	0.9	40.923	94.817	338.284	42.85
22	Longifolene	tricyclic	38.98	0.91	43.535	101.151	338.264	42.84
23	Kelsoene	tricyclic	39.49	0.922	11.693	103.505	338.216	42.83
24	Presilphiperfol-1-ene	tricyclic	38.98	0.91	43.535	101.151	338.203	42.83
25	Italicene	tricyclic	38.68	0.903	25.794	98.627	338.198	42.83
26	α-Copaene	tricyclic	39.49	0.922	11.693	103.505	338.195	42.83

Saturated compound No.	compound	Structure	Volumetric energy density (MJ/L)	Liquid density (kg/L)	Freezing point	Flash point （℃）	Specific impulse ISP(s)	Combustion heat (MJ/kg)
27	Δ^6-Protoilludene	tricyclic	38.96	0.91	43.535	101.151	338.127	42.81
28	β-Cubebene	tricyclic	39.16	0.915	-10.663	100.924	338.099	42.8
29	Marasmene	tricyclic	38.49	0.9	40.923	94.817	337.992	42.77
30	β-Bourbonene	tricyclic	39.43	0.922	11.693	103.505	337.973	42.77
31	β-Terrecyclene	tricyclic	38.6	0.903	25.794	98.627	337.88	42.75
32	Sterpurene	tricyclic	38.88	0.91	43.535	101.151	337.819	42.73
33	Neoclovene	tricyclic	39.87	0.933	33.237	96.168	337.818	42.73
34	Isolongifolene	tricyclic	39.17	0.917	48.843	98.311	337.792	42.72
35	Isocedrene	tricyclic	38.58	0.903	25.794	98.627	337.789	42.72
36	Prezizaene	tricyclic	38.53	0.903	25.794	98.627	337.564	42.67
37	Sativene	tricyclic	39.32	0.922	11.693	103.505	337.513	42.65
38	Rotundene	tricyclic	40.09	0.94	30.185	105.258	337.491	42.65
39	Seychellene	tricyclic	39.62	0.929	27.323	99.047	337.49	42.65
40	α-Cedrene	tricyclic	38.5	0.903	25.794	98.627	337.462	42.64
41	Duprezianene	tricyclic	38.5	0.903	25.794	98.627	337.44	42.64
42	Clovene	tricyclic	41.69	0.978	42.945	100.049	337.427	42.63
43	Helifolene	tricyclic	39.75	0.933	33.237	96.168	337.288	42.6
44	Khusiene	tricyclic	39.75	0.933	33.237	96.168	337.286	42.6
45	Modephene	tricyclic	39.44	0.926	14.126	93.448	337.264	42.59
46	Isocyclobazzanene	tricyclic	40.16	0.944	50.153	98.677	337.082	42.54
47	Silphiperfolene	tricyclic	39.09	0.919	7.375	96.476	337.069	42.54

Saturated compound No.	compound	Structure	Volumetric energy density (MJ/L)	Liquid density (kg/L)	Freezing point	Flash point （℃）	Specific impulse ISP(s)	Combustion heat (MJ/kg)
48	Capnellene	tricyclic	38.71	0.91	43.535	101.151	337.06	42.54
49	Cyclobazzanene	tricyclic	47	1.105	21.401	85.672	337.041	42.53
50	epi-isozizaene	tricyclic	38.4	0.903	25.794	98.627	337.018	42.53
51	Panaginsene	tricyclic	38.37	0.903	25.794	98.627	336.863	42.49
52	Hirsutene	tricyclic	38.66	0.91	43.535	101.151	336.835	42.48
53	Pentalenene	tricyclic	38.35	0.903	25.794	98.627	336.788	42.47
54	Modhephene	tricyclic	39.3	0.93	15.7	101	336	42.43
55	myltaylene	tricyclic	39.6	0.93	34.6	103	336	42.44
56	α−gurjunene	tricyclic	37.4	0.88	48.1	111	336	42.46
57	Prenopsene	tricyclic	38.6	0.91	55.8	108	336	42.39
58	Longiborneol	tricyclic	38.4	0.9	61.1	114	336	42.44
59	Koraiol	tricyclic	37.8	0.89	65.7	116	336	42.51
60	Isoishwarene	tricyclic	40.2	0.96	43.7	107	335	41.79

Table S7. Plasmids used in this study.

Plasmids	Description	Source
pRS426		Purchased from Biovector
pCas	pTEF1_iCAS9, pLac_Lacz, amp	Ref.[1]
pSH 47	*pRS426*, P_{GAL1}_*Cre*, *URA3*	Purchased from Biovector
pYeast3939	pRS426, GAL derived, URA, TCYC1-*ERG20*- P_{GAL1}-P_{GAL10} -FgJ03939-TADH1	This work
P1-vector	*pRS426, Leu2△:: Leu2_* P_{GAL1}_*ERG20*, P_{GAL10}_*LacZ, URA3(MssI)*	This work

P2-vector	pRS426,URA3△:: P_{GAL10}_ tHMG1, P_{GAL1}_LacZ, His 3 (MSSI)	This work
P3-vector	pRS426,GAL80△ : : P_{GAL10} P_{GAL1}_LacZ_HygMX (MSSI)	This work
pTPS-C15-21	pRS426, Leu2△:: Leu2_ P_{GAL1} _ERG20, P_{GAL10} _SGR2079, URA3(MssI)	This work
pTPS-C15-30	pRS426, Leu2△:: Leu2_ P_{GAL1} _ERG20, P_{GAL10}_Anec, URA3(MssI)	This work
pTPS-C15-61	pRS426, Leu2△:: Leu2_ P_{GAL1} _ERG20, P_{GAL10} _ZmTps21, URA3(MssI)	This work
pTPS-C15-69	pRS426, Leu2△:: Leu2_ P_{GAL1} _ERG20, P_{GAL10}_TPS2, URA3(MssI)	This work
pTPS-C15-47	pRS426, Leu2△:: Leu2_ P_{GAL1} _ERG20, P_{GAL10}_MrTPS2, URA3(MssI)	This work
pTPS-C15-53	pRS426, Leu2△:: Leu2_ P_{GAL1} _ERG20, P_{GAL10} _TPS6-B73, URA3(MssI)	This work
pTPS-C15-65	pRS426, Leu2△:: Leu2_ P_{GAL1} _ERG20, P_{GAL10}_SSDG_02809, URA3(MssI)	This work
pTPS-C15-49	pRS426, Leu2△:: Leu2_ P_{GAL1} _ERG20, P_{GAL10}_CLM1, URA3(MssI)	This work
pYH389	YPRCdelta15△:: URA3 TRP	This work
pZY528	GAL80Δ：：URA3 TRP	This work
pZY521	GAL80Δ：：URA3	This work
PZY566	GAL80Δ：：HIS 3 TRP	This work
pKZ761	pRS426, Leu2△:: Leu2_ P_{GAL1} _ERG20, P_{GAL10}_pents-sc, URA3(MssI)	This work
pKZ764	pRS426,URA3△:: P_{GAL10}_ tHMG1, P_{GAL1}_pents_sc, His 3 (MSSI)	This work
pKZ762	pRS426, Leu2△:: Leu2_ P_{GAL1} _ERG20, P_{GAL10}_SCO5222 sc, URA3(MssI)	This work
pKZ765	pRS426,URA3△:: P_{GAL10}_ tHMG1, P_{GAL1}_SCO5222 sc,His 3 (MSSI)	This work
pKZ771	pRS426,GAL80△ : : P_{GAL10} P_{GAL1}_SCO5222_sc_HygMX (MSSI)	This work
pXZ136	pRS426, Leu2△:: Leu2_ P_{GAL1}_ERG20, P_{GAL10}_Cgl06493-COP, URA3(MssI)	This work
pXZ137	pRS426,URA3△:: P_{GAL10}_ tHMG1, P_{GAL1}_Cgl06493-COP, His 3 (MSSI)	This work
pXZ139	pRS426,GAL80△ : : P_{GAL10} P_{GAL1}_Cgl06493-COP_HygMX (MSSI)	This work
pZY901	pRS426, Leu2△:: Leu2_ P_{GAL1} _ERG20, P_{GAL10}_AFS sc, URA3(MssI)	This work
pZY902	pRS426,URA3△:: P_{GAL10}_ tHMG1, P_{GAL1}_AFS sc,His 3 (MSSI)	This work
pZY903	pRS426,HIS3△:: P_{GAL10}_ P_{GAL1}_AFS sc,TRP 1 (MSSI)	This work
pTPS-Omp 7	pRS426, Leu2△:: Leu2_ P_{GAL1}_ERG20, P_{GAL10}_Omp 7, URA3(MssI)	This work
pPS01	pRS426,HIS3△:: P_{GAL10}_ERG20, P_{GAL1}_Omp 7, URA3 (XhoI)	This work
pTPS-SantS	pRS426, Leu2△:: Leu2_ P_{GAL1}_ERG20, P_{GAL10}_SantS, URA3(MssI)	This work
pSS01	pRS426,HIS3△:: P_{GAL10}_ERG20, P_{GAL1}_SantS, URA3 (XhoI)	This work

pTPS-Copu 2	*pRS426, Leu2△:: Leu2_* P_{GAL1} *_ERG20*, P_{GAL10}*_Copu 2, URA3(MssI)*	This work
pCS01	*pRS426,HIS3△::* P_{GAL10}*_ERG20*, P_{GAL1}*_Copu 2, URA3 (XhoI)*	This work
pTPS-BarS	*pRS426, Leu2△:: Leu2_* P_{GAL1}*_ERG20*, P_{GAL10}*_BarS, URA3(MssI)*	This work
pBS01	*pRS426,HIS3△::* P_{GAL10}*_ERG20*, P_{GAL1}*_BarS, URA3 (XhoI)*	This work

Table S8. Strains used in this study

Strains	Genome type	Source
JCR27	CEN.PK2-1D ChrXII-2△:: HygR_pTEF1_Cas9,<br>ChrXI-3△:: P_{GAL1}_ERG8, P_{GAL10}_tHMG1, P_{GAL7}_ERG12,<br>ChrX-3△:: P_{GAL1}_ERG13, P_{GAL10}_tHMG1,<br>ChrXII-4△:: P_{GAL1}_IDI1, P_{GAL10}_ERG10, P_{GAL7}_MVD1	Ref.[2]
JVA63	JCR27 Leu2△:: P_{GAL1}_ERG20, P_{GAL10}_pents-sc, LEU2	This work
JVA69	JVA63 URA3△:: P_{GAL10}_ tHMG1, P_{GAL1}_pents_sc, HIS 3	This work
JVA91	JVA69 GAL80Δ：：URA3 TRP	This work
JVA67	JCR27 Leu2△:: P_{GAL1}_ERG20, P_{GAL10}_SCO5222 sc, LEU2	This work
JVA71	JVA67 URA3△:: P_{GAL10}_ tHMG1, P_{GAL1}_SCO5222 sc,HIS 3	This work
JVA78	JVA71 GAL80△：：P_{GAL10} P_{GAL1}_SCO5222_sc_HygMX	This work
JVA85	JVA78 YPRCdelta15△:: URA3 TRP	This work
JZL10	JCR27 PGAL1_Cre, URA3	This work
JZL17	JZL10 Leu2△:: P_{GAL1}_ERG20, P_{GAL10}_Cgl06493-COP, LEU2	This work
JZL18	JZL17 URA3△:: P_{GAL10}_ tHMG1, P_{GAL1}_Cgl06493-COP, HIS 3	This work
JZL19	JZL18 GAL80△：：P_{GAL10} P_{GAL1}_Cgl06493-COP, HygMX	This work
JZL20	JZL19 YPRCdelta15△:: URA3 TRP	This work
JVA31	JCR27 Leu2△:: P_{GAL1}_ERG20, P_{GAL10}_AFS sc, LEU 2	This work
JSA8	JVA31 URA3△:: P_{GAL10}_ tHMG1, P_{GAL1}_AFS sc,HIS 3	This work
JSA14	JSA8 HIS3△:: P_{GAL10}_ P_{GAL1} _AFS sc,TRP 1	This work
JSA17	JSA14 GAL80Δ：：URA3	This work
JPS01	JCR27 HIS3△:: P_{GAL10}_ERG20, P_{GAL1}_Omp 7, URA 3	This work
JPS02	JPS01 Leu2△:: P_{GAL1}_ERG20, P_{GAL10}_Omp 7, LEU2	This work
JPS03	JPS01 GAL80Δ：：TRP HIS3	This work
JSS01	JCR27 HIS3△:: P_{GAL10}_ERG20, P_{GAL1}_SantS, URA 3	This work
JSS02	JSS01 Leu2△:: P_{GAL1}_ERG20, P_{GAL10}_SantS, LEU2	This work
JSS03	JSS02 GAL80Δ：：TRP HIS3	This work
JCS01	JCR27 HIS3△:: P_{GAL10}_ERG20, P_{GAL1}_Copu 2, URA 3	This work
JCS02	JCS01 Leu2△:: P_{GAL1}_ERG20, P_{GAL10}_Copu 2, LEU2	This work

JCS03	JCS02 GAL80Δ：：TRP HIS3	This work
JBS01	JCR27 *HIS3*△:: P_{GAL10}_*ERG20*, P_{GAL1}_*BarS, URA 3*	This work
JBS02	JBS01 Leu2△:: P_{GAL1}_*ERG20*, P_{GAL10}_BarS, LEU2	This work
JBS03	JBS02 GAL80Δ：：TRP HIS3	This work
JTPS-C15-21	JCR27 pTPS-C15-21	This work
JTPS-C15-30	JCR27 pTPS-C15-30	This work
JTPS-C15-61	JCR27 pTPS-C15-61	This work
JTPS-C15-69	JCR27 pTPS-C15-69	This work
JTPS-C15-47	JCR27 pTPS-C15-47	This work
JTPS-C15-53	JCR27 pTPS-C15-53	This work
JTPS-C15-65	JCR27 pTPS-C15-65	This work
JTPS-C15-49	JCR27 pTPS-C15-49	This work
JTPS-Omp 7	JCR27 pTPS-Omp 7	This work
JTPS-SantS	JCR27 pTPS-SantS	This work
JTPS-Copu 2	JCR27 pTPS-Copu 2	This work
JTPS-BarS	JCR27 pTPS-BarS	This work
JTPS-pents-sc	JCR27 pKZ761	This work
JTPS-SCO5222	JCR27 pKZ762	This work
JTPS-AFS	JCR27 pZY901	This work
JTPS-Cgl06493	JCR27 pXZ136	This work

Table S9. primers and fragments used in this study.

Plasmids	Fragments	Template	Primers	Sequence (5’-3’)
P1-vector Yeast assembly	P1	pRS426	P1-1-F	GGATAGTCTTTTGATGTGAGCTTGGTCGTTGTCGTTTAAACAACAGTTGCGCAGCCTGA
			3002-R	TCAACAGTATAGAACCGTGGATGATGTGGTtTCTACAGGATCTGACATTATTATTGTTG
	P2	pRS426	3003-F	ATAGTCCTCTTCCAACAATAATAATGTCAGATCCTGTAGAaACCACATCATCCACGGTT
			3003-R	AGGGCTTACCATCTGGCCCCAGTGCTGCAATGATACCGCGcGACCCACGCTCACCGGCT
	P3	pRS426	3004-F	TGATAAATCTGGAGCCGGTGAGCGTGGGTCgCGCGGTATCATTGCAGCACTGGGGCCAG
			P1-2-R	CGATAGCGCCCCTGTGTGTTCTCGTTATGTTTAAACTACTAGAGCTCCAGCTTTTGTTC
	P4	S288C	P1-3-F	ACTAAAGGGAACAAAAGCTGGAGCTCTAGTAGTTTAAACATAACGAGAACACACAGGGG
			P1-3-R	TAAATCATTAAAGTAACTTAAGGAGTTAAATTTAAGCAAGGATTTTCTTAACTTCTTCG
	P5	S288C	P1-4-F	AGAAGTTAAGAAAATCCTTGCTTAAATTTAACTCCTTAAGTTACTTTAATGATTTAGTT
			P1-4-R	GAGAAGGTTTTGGGACGCTCGAAGGCTTTAATTTGCGCGAAAAGCCAATTAGTGTGATA
	P6	CENPK2-1D	P1-5-F	CGATAAAGCACTTAGTATCACACTAATTGGCTTTTCGCGCAAATTAAAGCCTTCGAGC
			P1-5-R	GAATGGCGAATGGGACGCGCCCTGTAGCGGCTGAGGTCTCAACAGGCCCCTTTTCCTTT
	P7	pCas	P1-6-F	ATTACATGATATCGACAAAGGAAAAGGGGCCTGTTGAGACCTCAGCCGCTACAGGGCGC
			P1-6-R	aaaagtaagaatttttgaaaattcaatataaATGTGAGACCACCATGATTACGCCAAGC
	P8	CENPK2-1D	P1-7-F	TGGCGTAATCATGGTGGTCTCACATttatattgaattttcaaaaattcttactttttt
			P1-7-R	CTCTCTCCTAATTTCTTTTTCTGAAGCcattatagtttttctccttgacgttaaagta
	P9	S288C	P1-8-F	ctctatactttaacgtcaaggagaaaaaactataatgGCTTCAGAAAAAGAAATTAGGA
			P1-8-R	GAAATCTAATGTTTTATCGATTAGCGTTAGTTCTATTTGCTTCTCTTGTAAACTTTGTT
	P10	S288C	P1-9-F	GTTCTTGAACAAAGTTTACAAGAGAAGCAAATAGAACTAACGCTAATCGATAAAACATT
			P1-9-R	ATGTACAAATATCATAAAAAAAGAGAATCTTTTTAAAAAAAATCCTTGGACTAGTCACG
	P11	S288C	P1-10-F	CTAGTCCAAGGATTTTTTTTAAAAAGATTCTCTTTTTTTATGATATTTGTACATAAACT
			P1-10-R	GCGCCATTCGCCATTCAGGCTGCGCAACTGTTGTTTAAACGACAACGACCAAGCTCACA
P2-vector Yeast	P1	P1-vector	P2-1-F	ttccttctgcgagagtacatttgcccttaaacgGTTTAAACACAGACAAGCTGTGACCG
			P2-1-R	tgcttcaaaatacctggaattatctgcgtGTTTAAACGCTTTTGTTCCCTTTAGTGAGG
	P2	S288C	P2-2-F	ATTAACCCTCACTAAAGGGAACAAAAGCGTTTAAACacgcagataattccaggtatttt

assembly			P2-2-R	CGCGCGTAATACGACTCACTATAGGGCGAATTGGGTACcttcgtttcctgcaggttttt
	P3	S288C	P2-3-F	cccaactgcacagaacaaaaacctgcaggaaacgaagGTACCCAATTCGCCCTATAGTG
			P2-3-R	CTTGAGAAGGTTTTGGGACGCTCGAAGGCTTTAATTTGCTCACAGCTTGTCTGTAAGCG
	P4	CENPK2-1D	P2-4-F	CTTGTCTGCTCCCGGCATCCGCTTACAGACAAGCTGTGAGCAAATTAAAGCCTTCGAGC
			P2-4-R	TCGTTTGAAAGATGGGTCCGTCACCTGCATTAAATCCTAAACAGGCCCCTTTTCCTTTG
	P5	S288C	P2-5-F	TAATTACATGATATCGACAAAGGAAAAGGGGCCTGTTTAGGATTTAATGCAGGTGACGG
			P2-5-R	aaaaaagtaagaatttttgaaaattcaatataaATGGTTTTAACCAATAAAACAGTCAT
	P6	CENPK2-1D	P2-6-F	TGACTGTTTTATTGGTTAAAACCATttatattgaattttcaaaaattcttactttttt
			P2-6-R	ATCATGGTGGTCTCACATggtctcatatagttttttctccttgacgttaaagtatagag
	P7	pCas	P2-7-F	tttaacgtcaaggagaaaaaactatatgagaccATGTGAGACCACCATGATTACGCCAA
			P2-7-R	AAAAATTGATCTATCGATTTCAATTCAATTCAATtGAGACCTCAGCCGCTACAGGGCGC
	P8	CENPK2-1D	P2-8-F	GACGCGCCCTGTAGCGGCTGAGGTCTCaATTGAATTGAATTGAAATCGATAGATCAATT
			P2-8-R	ttgaagctctaatttgtgagtttagtatacatgcatttacAACGAACGCAGAATTTTCG
	P9	S288C	P2-9-F	AAGTTTAATAACTCGAAAATTCTGCGTTCGTTgtaaatgcatgtatactaaactcacaa
			P2-9-R	GCTCCCGGAGACGGTCACAGCTTGTCTGTGTTTAAACcgtttaagggcaaatgtactct
P3-vector Yeast assembly	P1	P1-vector	P3-1-F	gggcataatagcttctgtattcttggtggtctGTTTAAACAACAGTTGCGCAGCCTGA
			P3-1-R	GATTGCGCATACTTTGTGAAC
	P2	P1-vector	P3-2-F	TAGAAGAAACCGTTCATAATTTTCTG
			P3-2-R	agcttcgaatgccactacctagaccattGTTTAAACTACTAGAGCTCCAGCTTTTGTTC
	P3	CENPK2-1D	P3-3-F	ACTAAAGGGAACAAAAGCTGGAGCTCTAGTAGTTTAAACaatggtctaggtagtggcat
			P3-3-R	acattatacgaagttatattaagggttgtcgacctgcagcgacgggagtggaaagaacg
	P4	HygR	P3-4-F	caatctcgatagttggtttcccgttctttccactcccgtcgctgcaggtcgacaaccct
			P3-4-R	AGGGAAAAGCTGAATGGGCAGTTCGAATAccactagtggatctgatatcacctaataa
	P5	S288C	P3-5-F	tatacgaagttattaggtgatatcagatccactagtggTATTCGAACTGCCCATTCAGC
			P3-5-R	aaaaagtaagaatttttgaaaattcaatataaGTCTGAAGAATGAATGATTTGATGATT
	P6	CENPK2-1D	P3-6-F	TCATCAAATCATTCATTCTTCAGACttatattgaattttcaaaaattcttactttttt
			P3-6-R	GGCGTAATCATGGTGGTCTCACATggtctcatatagttttttctccttgacgttaaagt
	P7	pCas	P3-7-F	actttaacgtcaaggagaaaaaactatatgagaccATGTGAGACCACCATGATTACGCC

			P3-7-R	AAAAATTGATCTATCGATTTCAATTCAATTCAATtGAGACCTCAGCCGCTACAGGGCGC
	P8	CENPK2-1D	P3-8-F	GGGACGCGCCCTGTAGCGGCTGAGGTCTCaATTGAATTGAATTGAAATCGATAGATCAA
			P3-8-R	ttcgctgcactgggggccaagcacagggcaagatgcttAACGAACGCAGAATTTTCGAG
	P9	CENPK2-1D	P3-9-F	GCGTATTTTAAGTTTAATAACTCGAAAATTCTGCGTTCGTTaagcatcttgccctgtgc
			P3-9-R	CCATTCGCCATTCAGGCTGCGCAACTGTTGTTTAAACagaccaccaagaatacagaag
pTPS-C15-21 golden gate	P1	C15-TPS21	TPS21-F	AAAGGTCTCACTGTTTAAGCAGCGAAATGTCTAGATAAA
			TPS21-R	AAAGGTCTCaaATGTCTCAAATCACTTTGCCAG
	P2	P1-vector		
pTPS-C15-30 golden gate	P1	C15-TPS30	TPS30-F	AAAGGTCTCACTGTTTATTACTGGACATAATTTTTCAACAAACAA
			TPS30-R	AAAGGTCTCaaATGACACCAGATTTGGTTGC
	P2	P1-vector		
pTPS-C15-61 golden gate	P1	C15-TPS61	TPS61-F	AAAGGTCTCACTGTTTATTAAGCACATGGTTTCAAAAACAA
			TPS61-R	AAAGGTCTCaaATGGATGGTGATATTGCTGC
	P2	P1-vector		
pTPS-C15-69 Yeast assembly	P1	C15-TPS69	TPS69-F	ATGATATCGACAAAGGAAAAGGGGCCTGTTTATTAATATGGAACAGATTCAACCAACAA
			TPS69-R	aaaaaaagtaagaatttttgaaaattcaatataaATGGAATCTTGTTTGTCTTTCTCTT
	P2	P1-vector-bsai		
pTPS-C15-07 golden gate	P1	C15-TPS07	TPS07-F	GGGTCTCACTGTTTATTACAATAAAATTGGATCAACCAGCAA
			TPS07-R	AAAGGTCTCaaATGGAAGCTTTAGGTAATTTCGA
	P2	P1-vector		
pTPS-C15-47 golden gate	P1	C15-TPS47	TPS47-F	AAAGGTCTCACTGTTTATTAAGCCATAATTGGATCAACGAC
			TPS47-R	AAAGGTCTCaaATGTCTTTGCAAGAAAACGTTATC
	P2	P1-vector		
pTPS-C15-53	P1	C15-TPS53	TPS53-F	AAAGGTCTCACTGTTTATTACATCAAAACTGGTTTAACGTATAA
			TPS53-R	AAAGGTCTCaaATGGCTGCTCCAACATTGA

golden gate	P2	P1-vector		
pTPS-C15-65 golden gate	P1	C15-TPS65	TPS65-F	AAAGGTCTCACTGTTTATTAAGCAGATCTTTGAGCTGGA
			TPS65-R	AAAGGTCTCaaATGGAACCAGAATTAACAGTTCCA
	P2	P1-vector		
pTPS-C15-49 golden gate	P1	C15-TPS49	TPS49-F	AAAGGTCTCACTGTTTATTAGATTTTTTTAATAACTTCATGAACATCTG
			TPS49-R	AAAGGTCTCaaATGTTGGCTACTCCAACTTT
	P2	P1-vector		
pYH389 PCR	P1	30000B	3891-F	AGTTCATATTACTTTGGCCTGTCTTA
			3891-R	CTATAGGGCGAATTGGGTACTTTGCGAAACCCTATGCTC
	P2	PRS426	3892-F	AGAGCATAGGGTTTCGCAAAGTACCCAATTCGCCCTATAG
			5281-R	ATATATATAGTAATGTCGTTTCACAGCTTGTCTGTAAGCG
	P3	30000B	5282-F	CGCTTACAGACAAGCTGTGAAACGACATTACTATATATATAATATAGGAAGCATTTAAT
			3892-R	GCTCATCCCGACCTTCCATTCCTGATGCGGTATTTTCTCC
	P4	30000B	3893-F	AGGAGAAAATACCGCATCAGGAATGGAAGGTCGGGATGAG
			3893-R	CCGATGAACGCAAATGAAG
pZY528 PCR	P1	30000B	5281-F	cgcctgtctacaggataaagacgg
			5211-R	CGACTCACTATAGGGCGAATTGGGTACgacgggagtggaaagaacgg
	P2	30000B	5212-F	tcccgttctttccactcccgtcGTACCCAATTCGCCCTATAGTGAG
			5281-R	ATATATATAGTAATGTCGTTTCACAGCTTGTCTGTAAGCG
	P3	30000B	5282-F	CGCTTACAGACAAGCTGTGAAACGACATTACTATATATATAATATAGGAAGCATTTAAT
			5282-R	gttcgctgcactgggggccaagcacagggcaagatgcttCCTGATGCGGTATTTTCTCC
	P4	30000B	5283-F	TACCGCACAGATGCGTAAGGAGAAAATACCGCATCAGGaagcatcttgccctgtgcttg
			5283-R	aaatatgacccccaatatgagaaatt
pZY521 PCR	P1	30000B	5201-F	caatggtctaggtagtggcattcg
			5211-R	CGACTCACTATAGGGCGAATTGGGTACgacgggagtggaaagaacgg
	P2	hygR	5212-F	tcccgttctttccactcccgtcGTACCCAATTCGCCCTATAGTGAG

			5212-R	gccaagcacagggcaagatgcttTCACAGCTTGTCTGTAAGCGGA
	P3	30000B	5213-F	GCATCCGCTTACAGACAAGCTGTGAaagcatcttgccctgtgctt
			5203-R	gagaccaccaagaatacagaagctattat
pZY566 PCR	P1	30000B	5201-F	caatggtctaggtagtggcattcg
			5661-R	CTATAGGGCGAATTGGGTACgacgggagtggaaagaacg
	P2	30000B	5661-F	ccgttctttccactcccgtcGTACCCAATTCGCCCTATAGT
			5662-R	ATATATATAGTAATGTCGTTTCACAGCTTGTCTGTAAGCG
	P3	30000B	5662-F	TCCGCTTACAGACAAGCTGTGAAACGACATTACTATATATATAATATAGGAAGCAT
			5663-R	gccaagcacagggcaagatgcttCCTGATGCGGTATTTTCTCC
	P4	30000B	5663-F	GGAGAAAATACCGCATCAGGaagcatcttgccctgtgc
			5203-R	gagaccaccaagaatacagaagctattat
pKZ761 golden gate	P1	PentS.sc	7611-F	AAAGGTCTCACTGTTTAATGAGCTGAAGAACCTAATTCTTCCAAG
			7611-R	AAAGGTCTCaaATGCCACAAGATGTTGATTTTCATATTC
	P2	P1-vector		
pKZ764 golden gate	P1	PentS.sc	7641-F	AAAGGTCTCAtataATGCCACAAGATGTTGATTTTCATATTC
			7641-R	AAAGGTCTCACAATTAATGAGCTGAAGAACCTAATTCTTCC
	P2	P2-vector		
pKZ762 golden gate	P1	Sco5222-sc	7621-F	AAAGGTCTCACTGTTTATTTTTCACCTGCAGCTTCATTATTAACG
			7621-R	AAAGGTCTCaaATGCATGCATTTCCACATGGTAC
	P2	P1-vector		
pKZ765 golden gate	P1	Sco5222-sc	7651-F	AAAGGTCTCAtataATGCATGCATTTCCACATGGTAC
			7651-R	AAAGGTCTCACAATTATTTTTCACCTGCAGCTTCATTATTAACG
	P2	P2-vector		
pKZ771 golden gate	P1	Sco5222-sc	7711-F	AAAGGTCTCAtataATGCATGCATTTCCACATGGTAC
			7652-R	AAAGGTCTCACAATTTATTTTTCACCTGCAGCTTCATTATTAACG
	P2	P3-vector		
pXZ136 golden	P1	Cgl06493-COP	1361-F	aaaggtctcaCTGTTcAAACACCTGGAGATGGC
			1361-R	aaaggtctcaaATGTCTTCAGATGATCAATACAGAG

gate	P2	P1-vector		
pXZ137 golden gate	P1	Cgl06493 -COP	1371-F	AAAGGTCTCATATAATGTCTTCAGATGATCAATACAGAG
			1371-R	AAAGGTCTCACAATTTAAACACCTGGAGATGGCAA
	P2	P2-vector		
pXZ139 golden gate	P1	Cgl06493 -COP	1371-F	AAAGGTCTCATATAATGTCTTCAGATGATCAATACAGAG
			1371-R	AAAGGTCTCACAATTTAAACACCTGGAGATGGCAA
	P2	P3-vector		
pZY901 Yeast assembly	P1	AFS	901-F	TGATATCGACAAAGGAAAAGGGGCCTGTTTAATTAACCAATGGTTGAAATAACAAAGAC
			901-R	aaaaaaaaagtaagaatttttgaaaattcaatataaATGGAATTCAGAGTTCATTTGCA
	P2	P1-vector-bsai		
pZY902 Yeast assembly	P1	AFS	902-F	acctctatactttaacgtcaaggagaaaaaactataATGGAATTCAGAGTTCATTTGCA
			902-R	TTGATCTATCGATTTCAATTCAATTCAATTTAATTAACCAATGGTTGAAATAACAAAGA
	P2	P2-vector-bsai		
pZY903 Yeast assembly	P1	pRS426	9031-F	attggatctattgtctgcatccactgacggtttGTTTAAACACAGACAAGCTGTGACCG
			9031-R	aatcacaagaaatgggtggctggacgtttGTTTAAACGCTTTTGTTCCCTTTAGTGAGG
	P2	HIS	9032-F	GCGCAATTAACCCTCACTAAAGGGAACAAAAGCGTTTAAACaaacgtccagccacccat
			903-4-R	CTCCCGGCATCCGCTTACAGACAAGCTGTGAtttagtatattcttcgaagaaatcacat
	P3	TRP	903-1-F	tatataaagtaatgtgatttcttcgaagaatatactaaaTCACAGCTTGTCTGTAAGCG
			903-1-R	CATGAGGTCGCTCTTATTGACCACACCTCTACCGGGTACCCAATTCGCCCTATAGTGAG
	P4	CENPK2-1D	903-2-F	GCGCGTAATACGACTCACTATAGGGCGAATTGGGTACCCGGTAGAGGTGTGGTCAATAA
			9032-R	GTTATTTCAACCATTGGTTAATTAAGCGAATTTCTTATGATTTATGATTTTTATTATTA
	P5	AFS	9033-F	TAAAAATCATAAATCATAAGAAATTCGCTTAATTAACCAATGGTTGAAATAACAAAGAC
			9033-R	aaaaaaaaagtaagaatttttgaaaattcaatataaATGGAATTCAGAGTTCATTTGCA
	P6	CENPK2-1D	9034-F	CTTGCAAATGAACTCTGAATTCCATttatattgaattttcaaaaattcttacttttttt
			903-2-R	AAATCTTTGACTATTCAATCATTGCGCtatagtttttctccttgacgttaaagtatag
	P7	CENPK2-1D	903-3-F	acctctatactttaacgtcaaggagaaaaaactataGCGCAATGATTGAATAGTCAAAG
			903-3-R	accacaactaactttttcccgtcctccatctcttATTTGACACTTGATTTGACACTTCT
	P8	HIS	903-4-F	TAAATAAAAAAAAAAAGAAGTGTCAAATCAAGTGTCAAATaagagatggaggacgggaa

			9034-R	TGCAGCTCCCGGAGACGGTCACAGCTTGTCTGTGTTTAAACaaaccgtcagtggatgca
pTPS-Omp 7 golden gate	P1	omp 7	OMP7-F	AAAGGTCTCACTGTTCATAATTCGGAGATGTCAACGA
			OMP7-R	AAAGGTCTCaaATGCCAGAAACTTTTTACTTACCA
	P2	P1-vector		
pTPS-SantS golden gate	P1	SantS	SANTA-F	AAAGGTCTCACTGTTCAGTCATCCAATTTGACTGGG
			SANTA-R	AAAGGTCTCaaATGTCTACTCAACAAGTTTCTAGTG
	P2	P1-vector		
pTPS-Copu 2 golden gate	P1	Copu 2	COPA-F	AAAGGTCTCACTGTTCATTCAATTTCGGTTTCAACTTTAC
			COPA-R	AAAGGTCTCaaATGTCTACCATGGACCCATC
	P2	P1-vector		
pTPS-BarS Yeast assembly	P1	BarS	BarS-F	GGAAAAGGGGCCTGTTCATAATAAGATTGGATCGACCAAC
			BarS-R	gaaaattcaatataaATGGAAGCCTTGGGTAACTTTGATT
	P2	P1-vector BsaI		
pPS01 Yeast assembly	P1	omp 7	pYeast-Protoill-F1	TCATAAGAAATTCGCTCATAATTCGGAGATGTCAACGATT
			pYH328-Protoill-R1	gaaaattcaatataaATGCCAGAAACTTTTTACTTACCAG
	P2	pYeast39 39	pYH328-Protoill-P1-F1	AAAAAGTTTCTGGCATttatattgaattttcaaaaattct
			pYeast-P1-R1	GGCCGATTCATTAATGCAGCTGGCACGACA
	P3	pYeast39 39	pYeast-P2-F1	TGTCGTGCCAGCTGCATTAATGAATCGGCC
			pYeast-Protoill-P2-R1	ATCTCCGAATTATGAGCGAATTTCTTATGATTTATGATTT
pSS01 Yeast assembly	P1	SantS	pYeast-Santa-F1	TCATAAGAAATTCGCTCAGTCATCCAATTTGACTGGGTCC
			pYH328--Santa-R1	gaaaattcaatataaATGTCTACTCAACAAGTTTCTAGTG
	P2	pYeast39 39	pYH328--Santa-P1-F1	TTGTTGAGTAGACATttatattgaattttcaaaaattctt
			pYeast-P1-R1	GGCCGATTCATTAATGCAGCTGGCACGACA
	P3	pYeast39	pYeast-P2-F1	TGTCGTGCCAGCTGCATTAATGAATCGGCC

		39	pYeast-Santa-P2-R1	AAATTGGATGACTGAGCGAATTTCTTATGATTTATGATTT
pCS01 Yeast assembly	P1	Copu 2	pYeast-Copaene-F1	TCATAAGAAATTCGCTCATTCAATTTCGGTTTCAACTTTA
			pYH328--Copaene-R1	gaaaattcaatataaATGTCTACCATGGACCCATCTGAAT
	P2	pYeast39 39	pYH328--Copaene-P1-F1	GTCCATGGTAGACATttatattgaattttcaaaaattctt
			pYeast-P1-R1	GGCCGATTCATTAATGCAGCTGGCACGACA
	P3	pYeast39 39	pYeast-P2-F1	TGTCGTGCCAGCTGCATTAATGAATCGGCC
			pYeast-Copaene-P2-R1	ACCGAAATTGAATGAGCGAATTTCTTATGATTTATGATTT
pBS01 Yeast assembly	P1	BarS	pYeast-Thujo-F1	TCATAAGAAATTCGCTCATAATAAGATTGGATCGACCAAC
			pYH328--Thujo-R1	gaaaattcaatataaATGGAAGCCTTGGGTAACTTTGATT
	P2	pYeast39 39	pYH328--Thujo-P1-F1	ACCCAAGGCTTCCATttatattgaattttcaaaaattctt
			pYeast-P1-R1	GGCCGATTCATTAATGCAGCTGGCACGACA
	P3	pYeast39 39	pYeast-P2-F1	TGTCGTGCCAGCTGCATTAATGAATCGGCC
			pYeast-Thujo-P2-R1	CCAATCTTATTATGAGCGAATTTCTTATGATTTATGATTT

Table S10 Sources of terpenoid synthases.

synthase	Gene source	Sequence number
BarS	*Arabidopsis thaliana*	NCBI-NP_199276.1
BarS	*Arabidopsis thaliana*	NCBI-NP_199276.1
SCO5222	*Streptomyces*	NCBI-WP_011030119.1
PentS	*Streptomyces exfoliatus*	NCBI-Q55012.4
Cgl06493-COP	*Colletotrichum gloeosporioides*	N/A
Copu2	*Coniophora puteana*	NCBI-XP_007771895.1
AFS	*Malus domestica*	NCBI-Q84LB2.2
SantS	*Clausena lansium*	NCBI-ADR71055.1
OMP7	*Omphalotus olearius*	MUStwsD_GLEAN_10000831
TPS2	*valerian plant*	UniProtKB-J9R5V4 (TPS2_VALOF)
SGR2079	*Streptomyces griseus*	UniProtKB-B1W019 (GCOA_STRGG)
CLM1	*Fusarium graminearum*	UniProtKB-I1S104 (CLM1_GIBZE)
TPS6-B73	*Zea mays*	UniProtKB-Q5GJ60 (TPS6_MAIZE)
SSDG_02809	*Streptomyces pristinaespiralis*	UniProtKB-B5HDJ6 (SEDS_STRE2)
ZmTps21	*Zea mays*	UniProtKB-A0A291LSD6 (TPS21_MAIZE)
Anec	*Aspergillus aculeatus*	UniProtKB-A0A1L9WUI2 (ANEC_ASPA1)
MrTPS2	*Chamomilla recutita*	UniProtKB-I6R4V5 (TPS2_MATCR)

Table S11 the literatures of 122 sesquiterpenoids

Saturated compound No.	compound	References
1	Farnesene	Li R, *et al*. Reprogramming the chemodiversity of terpenoid cyclization by remolding the active site contour of epi-isozizaene synthase. *Biochemistry* **53**(7), 1155-1168 (2014).
2	Germacrene D	Agger S, Lopez-Gallego F, Schmidt-Dannert C. Diversity of sesquiterpene synthases in the basidiomycete Coprinus cinereus. *Molecular microbiology* **72**(5), 1181-1195(2009).
3	Helminthogermacrene	Agger S, Lopez-Gallego F, Schmidt-Dannert C. Diversity of sesquiterpene synthases in the basidiomycete Coprinus cinereus. *Molecular microbiology* **72**(5), 1181-1195(2009).
4	α-Humulene	Chen F, Tholl D, D'Auria J C, *et al*. Biosynthesis and emission of terpenoid volatiles from Arabidopsis flowers. *The Plant Cell* **15**(2), 481-494 (2003).
5	β-Elemene	Chen F, Tholl D, D'Auria J C, *et al*. Biosynthesis and emission of terpenoid volatiles from Arabidopsis flowers. *The Plant Cell* **15**(2), 481-494 (2003).
6	(E)- β-Caryophyllene	Chen F, Tholl D, D'Auria J C, *et al*. Biosynthesis and emission of terpenoid volatiles from Arabidopsis flowers. *The Plant Cell* **15**(2), 481-494 (2003).

7	3，7-di-*epi*-trifara-9,14-diene	Sonwa M M, König W A, Lahlou E H, *et al*. Sesquiterpene hydrocarbons with trifarane backbone in the liverwort Trocholejeunea sandvicensis. *Phytochemistry* **57**(4), 499-506 (2001).
8	Acora-3,5-diene	Li R, Chou W K W, Himmelberger J A, *et al*. Reprogramming the chemodiversity of terpenoid cyclization by remolding the active site contour of epi-isozizaene synthase. *Biochemistry* **53**(7), 1155-1168 (2014).
9	Acoradiene	Faraldos J A, O’Maille P E, Dellas N, *et al*. Bisabolyl-Derived Sesquiterpenes from Tobacco 5-Epi-aristolochene Synthase-Catalyzed Cyclization of (2 Z, 6 E)-Farnesyl Diphosphate. *Journal of the American Chemical Society* **132**(12),4281-4289 (2010).
10	Aristolochene	Cane D E, Kang I. Aristolochene synthase: purification, molecular cloning, high-level expression in Escherichia coli, and characterization of the Aspergillus terreus cyclase. *Archives of biochemistry and biophysics* **376**(2), 354-364 (2000).
11	Asteriscene	Fricke C, Hardt I H, König W A, *et al*. Sesquiterpenes from Lippia integrifolia essential oil. *Journal of natural products* **62**(5), 694-696 (1999).
12	Bazzanene	Hong Y J, Tantillo D J. Branching out from the bisabolyl cation. Unifying mechanistic pathways to barbatene, bazzanene, chamigrene, chamipinene, cumacrene, cuprenene, dunniene, isobazzanene, iso-γ-bisabolene, isochamigrene, laurene, microbiotene, sesquithujene, sesquisabinene, thujopsene, trichodiene, and widdradiene sesquiterpenes. *Journal of the American Chemical Society* **136**(6), 2450-2463 (2014).
13	Bicycloelemene	Trung H D, Thang T D, Ban P H, *et al*. Terpene constituents of the leaves of five Vietnamese species of Clausena (Rutaceae). *Natural Product Research* **28**(9), 622-630 (2014).
14	Bicyclogermacrene	Köpke D, Schröder R, Fischer H M, *et al*. Does egg deposition by herbivorous pine sawflies affect transcription of sesquiterpene synthases in pine?. *Planta* **228**(3), 427-438 (2008).
15	Brasila 1,10-diene	Quin M B, Flynn C M, Wawrzyn G T, *et al*. Mushroom hunting by using bioinformatics: application of a predictive framework facilitates the selective identification of sesquiterpene synthases in basidiomycota. *ChemBioChem* **14**(18), 2480-2491 (2013).
16	Brasila 5,6-diene	Melching S, König W A. Sesquiterpenes from the essential oil of the liverwort Conocephalum conicum. *Phytochemistry* **51**(4), 517-523 (1999).
17	Cumacrene	Cool L G. Sesquiterpenes from Cupressus macrocarpa foliage. *Phytochemistry* **66**(2), 249-260 (2005).
18	Dactylene	Elyakov G B, Stonik V A, Makar'Eva T N. Heterocyclic compounds of marine organisms. *Chemistry of Heterocyclic Compounds* **13**(4), 345-359 (1977).
19	Daucene	Bartelt R J, Zilkowski B W, Cossé A A, *et al*. Male-produced aggregation pheromone of the lesser mealworm beetle, Alphitobius diaperinus. *Journal of chemical ecology* **35**(4), 422-434 (2009).
20	Drimenene	Javidnia K, Miri R, Soltani M, et al. Chemical constituents of the essential oil of Ajuga austro-iranica Rech. f.(Lamiaceae) from Iran. *Journal of Essential Oil Research* **22**(5), 392-394 (2010).
21	Epi-zonarene	Li R, Chou W K W, Himmelberger J A, *et al*. Reprogramming the chemodiversity of terpenoid cyclization by remolding the active site contour of epi-isozizaene synthase. *Biochemistry* **53**(7),1155-1168 (2014).
22	Eremophilene	Schifrin A, Ly T T B, Gϋnnewich N, *et al*. Characterization of the Gene Cluster CYP264B1-geoA from Sorangium cellulosum So ce56: Biosynthesis of (+)-Eremophilene and Its Hydroxylation. *ChemBioChem* **16**(2), 337-344

		(2015).
23	Fusariumdiene	Bian G, Hou A, Yuan Y, *et al*. Metabolic engineering-based rapid characterization of a sesquiterpene cyclase and the skeletons of fusariumdiene and fusagramineol from Fusarium graminearum. *Organic letters* **20**(6), 1626-1629 (2018).
24	Gleenene	Klapschinski T A, Rabe P, Dickschat J S. Pristinol, a sesquiterpene alcohol with an unusual skeleton from Streptomyces pristinaespiralis. *Angewandte Chemie International Edition* **55**(34), 10141-10144 (2016).
25	Gorgonene	Hong C Y, Tsao N W, Wang S Y, *et al*. Cloning and functional characterization of three sesquiterpene synthase genes from Chamaecyparis formosensis Matsumura. *Plant Science* 111315 (2022).
26	Guaiadiene	Niculau EDS, Alves PB, Nogueira PCL, *et al*. Chemical Profile and Use of the Peat as an Adsorbent for Extraction of Volatile Compounds from Leaves of Geranium (Pelargonium graveolens L' Herit). *Molecules* **25**(21),4923 (2020).
27	Guaie-9,11-diene	Niculau EDS, Alves PB, Nogueira PCL, *et al*. Chemical Profile and Use of the Peat as an Adsorbent for Extraction of Volatile Compounds from Leaves of Geranium (Pelargonium graveolens L' Herit). *Molecules* **25**(21),4923 (2020).
28	Herbertene	Matsuo A, Yuki S, Nakayama M, *et al*. (–)-Herbertene, an aromatic sesquiterpene with a novel carbon skeleton from the liverwort Herberta adunca. Journal of the Chemical Society, *Chemical Communications* **16**, 864-865 (1981).
29	Himachala-9,11-diene	Beran F, Rahfeld P, Luck K, *et al*. Novel family of terpene synthases evolved from trans-isoprenyl diphosphate synthases in a flea beetle. *Proceedings of the National Academy of Sciences* **113**(11), 2922-2927 (2016).
30	Illudalene	Kokubun T, Scott-Brown A, Kite G C, *et al*. Protoilludane, illudane, illudalane, and norilludane sesquiterpenoids from Granulobasidium vellereum. *Journal of natural products* **79**(6), 1698-1701 (2016).
31	Isobazzanene	König W A , Rieck A , Saritas Y , *et al*. Sesquiterpene hydrocarbons in the essential oil of Meum athamanticum. *Phytochemistry* **42**(2),461-464 (1996).
32	Isochamigrene	Hong Y J, Tantillo D J. Branching out from the bisabolyl cation. Unifying mechanistic pathways to barbatene, bazzanene, chamigrene, chamipinene, cumacrene, cuprenene, dunniene, isobazzanene, iso-γ-bisabolene, isochamigrene, laurene, microbiotene, sesquithujene, sesquisabinene, thujopsene, trichodiene, and widdradiene sesquiterpenes. *Journal of the American Chemical Society* **136**(6), 2450-2463 (2014).
33	Isodaucene	Aladedunye F A, Benn M H, Okorie D A. Sesquiterpenes from Culcasia scandens P. Beauv. *Natural Product Research* **22**(10), 879-883 (2008).
34	Macrocarpene	Köllner T G, Schnee C, Li S, *et al*. Protonation of a neutral (S)-β-bisabolene intermediate is involved in (S)-β-macrocarpene formation by the maize sesquiterpene synthases TPS6 and TPS11. *Journal of biological chemistry* **283**(30), 20779-20788 (2008).
35	Nardosina-7,9,11-triene	de Sena Filho J G, Quin M B, Spakowicz D J, *et al*. Genome of Diaporthe sp. provides insights into the potential inter-phylum transfer of a fungal sesquiterpenoid biosynthetic pathway. *Fungal biology* **120**(8), 1050-1063 (2016).
36	Neotrifaradiene	Sonwa M M, König W A, Lahlou E H, *et al*. Sesquiterpene hydrocarbons with trifarane backbone in the liverwort Trocholejeunea sandvicensis. *Phytochemistry* **57**(4), 499-506 (2001).
37	Pinguisene	Coulerie P, Thouvenot L, Nour M, *et al*. Chemical originalities of New Caledonian liverworts from Lejeuneaceae family. *Natural Product Communications* **10**(9), 1934578X1501000903(2015).
38	sandvicene	Sonwa M M, König W A, Lahlou E H, *et al*. Sesquiterpene hydrocarbons with trifarane backbone in the liverwort Trocholejeunea sandvicensis. *Phytochemistry* **57**(4), 499-506 (2001).

39	Selina-4,11-diene	Quin M B, Michel S N, Schmidt-Dannert C. Moonlighting Metals: Insights into Regulation of Cyclization Pathways in Fungal Δ6-Protoilludene Sesquiterpene Synthases. *ChemBioChem* **16**(15), 2191-2199 (2015).
40	Trichodiene	Vedula L S, Jiang J, Zakharian T, *et al*. Structural and mechanistic analysis of trichodiene synthase using site-directed mutagenesis, Probing the catalytic function of tyrosine-295 and the asparagine-225/serine-229/glutamate-233–Mg2+ B motif . *Archives of biochemistry and biophysics* **469**(2), 184-194 (2008).
41	Valencene	Schifrin A, Ly T T B, Günnewich N, *et al*. Characterization of the Gene Cluster CYP264B1-geoA from Sorangium cellulosum So ce56, Biosynthesis of (+)-Eremophilene and Its Hydroxylation. *ChemBioChem* **16**(2), 337-344 (2015).
42	Valerene	Dominguez R A. Valerian: its value as a sedative hypnotic. Natural medications for psychiatric disorders, considering the alternatives. 1st ed. Philadelphia, Lippincott Williams and Wilkins 132-146 (2002).
43	Vetispiradiene	Trapp S C, Croteau R B. Genomic organization of plant terpene synthases and molecular evolutionary implications. *Genetics* **158**(2), 811-832 (2001).
44	Widdrene	Kerth C R , Wall K R , Miller R K , *et al*. Effects of feeding juniper as a roughage on feedlot performance, carcass measurements, meat sensory attributes, and volatile aroma compounds of yearling Rambouillet wethers. *Journal of Animal Science* **97**(2019).
45	α-Bulnesene	Faraldos J A, Wu S, Chappell J, *et al*. Doubly deuterium-labeled patchouli alcohol from cyclization of singly labeled [2-2H1] farnesyl diphosphate catalyzed by recombinant patchoulol synthase. *Journal of the American Chemical Society* **132**(9), 2998-3008 (2010).
46	α-Chamigrene	Tholl D, Chen F, Petri J, *et al*. Two sesquiterpene synthases are responsible for the complex mixture of sesquiterpenes emitted from Arabidopsis flowers. *The Plant Journal* **42**(5), 757-771 (2005).
47	α-Cuprenene	Agger S, Lopez-Gallego F, Schmidt-Dannert C. Diversity of sesquiterpene synthases in the basidiomycete Coprinus cinereus. *Molecular microbiology* **72**(5), 1181-1195 (2009).
48	α-Himachalene	Weyerstahl, P., Kaul, V. K., Weirauch, M., *et al*. Volatile Constituents of Artemisia vestita Oil1. *Planta medica* **53**(1), 66–72 (1987).
49	α-Muurolene	Lee S, Chappell J. Biochemical and genomic characterization of terpene synthases in Magnolia grandiflora . *Plant Physiology* **147**(3), 1017-1033 (2008).
50	α-Neocallitropsene	Cavaleiro C , Gon?Alves M J , Serra D , *et al*. Composition of a volatile extract of Eryngium duriaei subsp. juresianum (M. Laínz) M. Laínz, signalised by the antifungal activity. *J Pharm Biomed Anal* **54**(3),619-622 (2011).
51	α-Selinene	Wang L, Wu Y, Huang T, *et al*. Chemical compositions, antioxidant and antimicrobial activities of essential oils of Psidium guajava L. Leaves from different geographic regions in China. *Chemistry & biodiversity* **14**(9), e1700114 (2017).
52	β-acoradiene	Li R, Chou W K W, Himmelberger J A, *et al*. Reprogramming the chemodiversity of terpenoid cyclization by remolding the active site contour of epi-isozizaene synthase. *Biochemistry* **53**(7), 1155-1168 (2014).
53	β-selinene	Wang L, Wu Y, Huang T, *et al*. Chemical compositions, antioxidant and antimicrobial activities of essential oils of Psidium guajava L. Leaves from different geographic regions in China. *Chemistry & biodiversity* **14**(9), e1700114 (2017).
54	δ-Cadinene	Sonwa M M, König W A. Chemical study of the essential oil of Cyperus rotundus. *Phytochemistry* **58**(5), 799-810 (2001).
55	γ-Muurolene	Sonwa M M, König W A. Chemical study of the essential oil of Cyperus rotundus. *Phytochemistry* **58**(5), 799-810 (2001).
56	Amorphadiene	Köpke D, Schröder R, Fischer H M, *et al*. Does egg deposition by herbivorous pine sawflies affect transcription of sesquiterpene synthases in pine?. *Planta* **228**(3), 427-438 (2008).

57	4-epi-β-patchoulene	Faraldos J A, Wu S, Chappell J, *et al*. Doubly deuterium-labeled patchouli alcohol from cyclization of singly labeled [2-2H1] farnesyl diphosphate catalyzed by recombinant patchoulol synthase. *Journal of the American Chemical Society* **132**(9), 2998-3008 (2010).
58	African-1-ene	Reddy N S, Goud T V, Venkateswarlu Y. Seco-sethukarailin, a novel diterpenoid from the soft coral Sinularia dissecta. *Journal of natural products* **65**(7), 1059-1060 (2002).
59	Aristolene	Tada A , Rgac A , Emilly J.S.P. de Lima b, *et al*. In vitro and in vivo inhibition of HCT116cells by essential oils from bark and leaves of Virola surinamensis (Rol. ex Rottb.) Warb. (Myristicaceae) - ScienceDirect. *Journal of Ethnopharmacology* (2020).
60	Aromadendrene	Wang L, Wu Y, Huang T, *et al*. Chemical compositions, antioxidant and antimicrobial activities of essential oils of Psidium guajava L. Leaves from different geographic regions in China. *Chemistry & biodiversity* **14**(9), e1700114 (2017).
61	Bicycloopposítene	Klapschinski T A, Rabe P, Dickschat J S. Pristinol, a sesquiterpene alcohol with an unusual skeleton from Streptomyces pristinaespiralis. *Angewandte Chemie International Edition* **55**(34), 10141-10144 (2016).
62	Capnellene	Jean Y H, Chen W F, Sung C S, *et al*. Capnellene, a natural marine compound derived from soft coral, attenuates chronic constriction injury-induced neuropathic pain in rats. *British journal of pharmacology* **158**(3), 713-725 (2009).
63	Caryolene	Hong Y J, Tantillo D J. Feasibility of intramolecular proton transfers in terpene biosynthesis–guiding principles. *Journal of the American Chemical Society* **137**(12), 4134-4140 (2015).
64	Clovene	Ferguson G, Hawley D M, McKillop T F W, *et al*. The structure and synthesis of pseudoclovene. *Chemical Communications* **21**, 1123-1125(1967).
65	Cyclobazzanene	Asakawa Y, Baser K H C, Erol B, *et al*. Volatile components of some selected Turkish liverworts. *Natural Product Communications* **13**(7), 1934578X1801300729 (2018).
66	Cyclocaryophyllene	Tkachev A V. The chemistry of caryophyllene and related compounds. *Chemistry of Natural Compounds* **23**(4), 393-412 (1987).
67	Duprezianene	Barrero A F, Alvarez-Manzaneda E, Lara A. Novel tricyclic sesquiterpenes from Juniperus thurifera L. Chemical confirmation of the duprezianane skeleton. *Tetrahedron letters* **37**(21), 3757-3760 (1996).
68	epi-fusagramineol	Bian G, Hou A, Yuan Y, *et al*. Metabolic engineering-based rapid characterization of a sesquiterpene cyclase and the skeletons of fusariumdiene and fusagramineol from Fusarium graminearum. *Organic letters* **20**(6), 1626-1629 (2018).
69	epi-isozizaene	Li R, Chou W K W, Himmelberger J A, *et al*. Reprogramming the chemodiversity of terpenoid cyclization by remolding the active site contour of epi-isozizaene synthase. *Biochemistry* **53**(7), 1155-1168 (2014).
70	Helifolene	Li R, Chou W K W, Himmelberger J A, *et al*. Reprogramming the chemodiversity of terpenoid cyclization by remolding the active site contour of epi-isozizaene synthase. *Biochemistry* **53**(7), 1155-1168 (2014).
71	Hirsutene	Quin M B, Flynn C M, Wawrzyn G T, *et al*. Mushroom hunting by using bioinformatics: application of a predictive framework facilitates the selective identification of sesquiterpene synthases in basidiomycota. *ChemBioChem* **14**(18), 2480-2491 (2013).
72	Illudene	Kokubun T, Scott-Brown A, Kite G C, *et al*. Protoilludane, illudane, illudalane, and norilludane sesquiterpenoids from Granulobasidium vellereum. *Journal of natural products* **79**(6), 1698-1701 (2016).
73	Isocedrene	Zdero C, Bohlmann F, Niemeyer HM. Isocedrene and Guaiane Derivatives from Pleocarphus revolutus. *J Nat Prod* **51**(3),509-512 (1988).
74	Isocyclobazzanene	Wu C L, Liu S. New Sesquiterpenes from Liverworts and from the Rearrangement of β-Bazzanene. *Tetrahedron* **39**(16), 2657-2661 (1983).
75	Isolactarene	Ishihara A, Ashida C, Ube N, *et al*. Isolation of isolactarane sesquiterpenes from a Phlebia tremellosa culture filtrate and their growth promotion effects on lettuce roots. *J Pestic Sci* **44**(1),9-14 (2019).

76	Isolongifolene	Chen CY, Chien SC, Tsao NW, *et al*. Metabolite Profiling and Comparison of Bioactivity in Antrodia cinnamomea and Antrodia salmonea Fruiting Bodies. *Planta Med* **82**(3),244-249 (2016).
77	Italicene	Zheljazkov VD, Semerdjieva I, Yankova-Tsvetkova E, *et al*. Chemical Profile and Antimicrobial Activity of the Essential Oils of Helichrysum arenarium (L.) Moench. and Helichrysum italicum (Roth.) G. Don. *Plants* (Basel) **11**(7),951 (2022).
78	Kelsoene	Fietz-Razavian S, Schulz S, Dix I, Jones PG. Revision of the absolute configuration of the tricyclic sesquiterpene (+)-kelsoene by chemical correlation and enantiospecific total synthesis of its enantiomer. *Chem Commun* (Camb) (20),2154-2155 (2001).
79	Khusiene	Sharma M K, Banwell M G, Willis A C. Generation of (+)-prezizanol,(+)-prezizaene, and the ent-β-isopipitzol framework via cationic rearrangement of khusiol and related compounds. *Asian Journal of Organic Chemistry* **3**(5), 632-637 (2014).
80	Longibornene	Calderón-Oropeza M A, Ramírez-Briones E, Rodríguez-García G, *et al*. Metabolic Correlations of Salvia dugesii Fernald and Salvia gesneriiflora Lindl. & Paxton with Native Salvia Plants from Four Continents Using Essential Oils Compositions. *Records of Natural Products* **15**(4), 323 (2021).
81	Longifolene	Köpke D, Schröder R, Fischer H M, *et al*. Does egg deposition by herbivorous pine sawflies affect transcription of sesquiterpene synthases in pine?. *Planta* **228**(3), 427-438 (2008).
82	Maaliene	Elgamal AM, Ahmed RF, Abd-ElGawad AM, *et al*. Chemical Profiles, Anticancer, and Anti-Aging Activities of Essential Oils of Pluchea dioscoridis (L.) DC. and Erigeron bonariensis L. *Plants (Basel)* **10**(4),667 (2021).
83	Marasmene	Liermann JC, Thines E, Opatz T, Anke H. Drimane sesquiterpenoids from Marasmius sp. inhibiting the conidial germination of plant-pathogenic fungi. *J Nat Prod*. **75**(11),1983-1986 (2012).
84	Microbiotene	Hong Y J, Tantillo D J. Branching out from the bisabolyl cation. Unifying mechanistic pathways to barbatene, bazzanene, chamigrene, chamipinene, cumacrene, cuprenene, dunniene, isobazzanene, iso-γ-bisabolene, isochamigrene, laurene, microbiotene, sesquithujene, sesquisabinene, thujopsene, trichodiene, and widdradiene sesquiterpenes. *Journal of the American Chemical Society* **136**(6), 2450-2463 (2014).
85	Modephene	Joseph-Nathan P, Reyes-Trejo B, Morales-Ríos MS. Molecular rearrangements of (-)-modhephene and (-)-isocomene to a (-)-triquinane. *J Org Chem*. **71**(12),4411-4417 (2006).
86	Neoclovene	Richter R, Basar S, Koch A, *et al*. Three sesquiterpene hydrocarbons from the roots of Panax ginseng CA Meyer (Araliaceae). *Phytochemistry* **66**(23), 2708-2713 (2005).
87	Panaginsene	Quin M B, Flynn C M, Wawrzyn G T, *et al*. Mushroom hunting by using bioinformatics: application of a predictive framework facilitates the selective identification of sesquiterpene synthases in basidiomycota. *ChemBioChem* **14**(18), 2480-2491 (2013).
88	Panasinsene	Richter R, Basar S, Koch A, *et al*. Three sesquiterpene hydrocarbons from the roots of Panax ginseng CA Meyer (Araliaceae). *Phytochemistry* **66**(23), 2708-2713 (2005).
89	Pentalenene	Agger S, Lopez-Gallego F, Schmidt-Dannert C. Diversity of sesquiterpene synthases in the basidiomycete Coprinus cinereus. *Molecular microbiology* **72**(5), 1181-1195 (2009).
90	Presilphiperfol-1-ene	Quin M B, Flynn C M, Wawrzyn G T, et al. Mushroom hunting by using bioinformatics: application of a predictive framework facilitates the selective identification of sesquiterpene synthases in basidiomycota. ChemBioChem 14(18), 2480-2491 (2013).
91	Prezizaene	Faraldos J A, O'Maille P E, Dellas N, *et al*. Bisabolyl-Derived Sesquiterpenes from Tobacco 5-Epi-aristolochene Synthase-Catalyzed Cyclization of (2 Z, 6 E)-Farnesyl Diphosphate. *Journal of the American Chemical Society* **132**(12), 4281-4289 (2010).
92	Rotundene	Sonwa M M , Knig W A . Chemical study of the essential oil of Cyperus

		rotundus. *Phytochemistry* **58**(5),799-810 (2001).
93	Sativene	Agger S, Lopez-Gallego F, Schmidt-Dannert C. Diversity of sesquiterpene synthases in the basidiomycete Coprinus cinereus. *Molecular microbiology* **72**(5), 1181-1195 (2009).
94	Seychellene	Faraldos J A, Wu S, Chappell J, *et al*. Doubly deuterium-labeled patchouli alcohol from cyclization of singly labeled [2-2H1] farnesyl diphosphate catalyzed by recombinant patchoulol synthase. *Journal of the American Chemical Society* **132**(9), 2998-3008 (2010).
95	Silphinene	Pinedo Rivilla C, González Collado I, Wang C M, *et al*. The Sesquiterpene Synthase from the Botrydial Biosynthetic Gene Cluster of the Phytopathogen Botrytis cinerea. *ACS CHEMICAL BIOLOGY* **3**(12), 791-801 (2008).
96	Silphiperfolene	Pinedo Rivilla C, González Collado I, Wang C M, *et al*. The Sesquiterpene Synthase from the Botrydial Biosynthetic Gene Cluster of the Phytopathogen Botrytis cinerea. *ACS CHEMICAL BIOLOGY* **3**(12), 791-801 (2008).
97	Sterpurene	Quin M B, Flynn C M, Wawrzyn G T, *et al*. Mushroom hunting by using bioinformatics: application of a predictive framework facilitates the selective identification of sesquiterpene synthases in basidiomycota. *ChemBioChem* **14**(18), 2480-2491 (2013).
98	Thujopsene	Chen F, Tholl D, D'Auria J C, *et al*. Biosynthesis and emission of terpenoid volatiles from Arabidopsis flowers. *The Plant Cell* **15**(2), 481-494 (2003).
99	Zizaene	Li R, Chou W K W, Himmelberger J A, *et al*. Reprogramming the chemodiversity of terpenoid cyclization by remolding the active site contour of epi-isozizaene synthase. *Biochemistry* **53**(7), 1155-1168 (2014).
100	α-Barbatene	Chen F, Tholl D, D'Auria J C, *et al*. Biosynthesis and emission of terpenoid volatiles from Arabidopsis flowers. *The Plant Cell* **15**(2), 481-494 (2003).
101	α-Cedrene	Faraldos J A, O'Maille P E, Dellas N, *et al*. Bisabolyl-Derived Sesquiterpenes from Tobacco 5-Epi-aristolochene Synthase-Catalyzed Cyclization of (2 Z, 6 E)-Farnesyl Diphosphate. *Journal of the American Chemical Society* **132**(12), 4281-4289 (2010).
102	α-Copaene	Chen F, Tholl D, D'Auria J C, *et al*. Biosynthesis and emission of terpenoid volatiles from Arabidopsis flowers. *The Plant Cell* **15**(2), 481-494 (2003).
103	α-isocomene	Pinedo Rivilla C, González Collado I, Wang C M, *et al*. The Sesquiterpene Synthase from the Botrydial Biosynthetic Gene Cluster of the Phytopathogen Botrytis cinerea. *ACS CHEMICAL BIOLOGY* **3**(12), 791-801 (2008).
104	α-santalene	Srivastava P L, Daramwar P P, Krithika R, *et al*. Functional characterization of novel sesquiterpene synthases from Indian sandalwood, Santalum album. *Scientific reports* **5**(1), 1-12 (2015).
105	β-Bourbonene	Crock J, Wildung M, Croteau R. Isolation and bacterial expression of a sesquiterpene synthase cDNA clone from peppermint (Mentha x piperita, L.) that produces the aphid alarm pheromone (E)-β-farnesene. *Proceedings of the National Academy of Sciences* **94**(24), 12833-12838 (1997).
106	β-Cubebene	Agger S, Lopez-Gallego F, Schmidt-Dannert C. Diversity of sesquiterpene synthases in the basidiomycete Coprinus cinereus. *Molecular microbiology* **72**(5), 1181-1195 (2009).
107	β-Longipinene	Quin M B, Flynn C M, Wawrzyn G T, *et al*. Mushroom hunting by using bioinformatics: application of a predictive framework facilitates the selective identification of sesquiterpene synthases in basidiomycota. *ChemBioChem* **14**(18), 2480-2491 (2013).
108	β-Terrecyclene	Barquera-Lozada J E , Cuevas G . Role of Carbocation's Flexibility in Sesquiterpene Biosynthesis: Computational Study of the Formation Mechanism of Terrecyclene. *Journal of Organic Chemistry* **76**(6),1572-7 (2011).
109	Δ^6-Protoilludene	Quin M B, Michel S N, Schmidt-Dannert C. Moonlighting Metals: Insights into Regulation of Cyclization Pathways in Fungal Δ6-Protoilludene Sesquiterpene Synthases. *ChemBioChem* **16**(15), 2191-2199 (2015).
110	Isoishwarene	Raga DD, Espiritu RA, Shen CC, Ragasa CY. A bioactive sesquiterpene from Bixa orellana. *Journal of natural medicines* **65**(1), 206–211 (2012).

111	myltaylene	Harinantenaina L, Asakawa Y. Chemical constituents of Malagasy liverworts. 6. A myltaylane caffeate with nitric oxide inhibitory activity from Bazzania nitida. *Journal of natural products* **70**(5), 856-858 (2007).
112	Modhephene	Pinedo Rivilla C, González Collado I, Wang C M, *et al*. The Sesquiterpene Synthase from the Botrydial Biosynthetic Gene Cluster of the Phytopathogen Botrytis cinerea. *ACS CHEMICAL BIOLOGY* **3**(12), 791-801 (2008).
113	Prenopsene	M Meléndez-Rodríguez, CM Cerda-García-Rojas, Joseph-Nathan P. Quirogane, Prenopsane, and Patzcuarane Skeletons Obtained by Photochemically Induced Molecular Rearrangements of Longipinene Derivatives. *Journal of Natural Products* **65**(10),1398-411 (2002).
114	Longiborneol	Schmidt R, Durling MB, de Jager V, *et al*. Deciphering the genome and secondary metabolome of the plant pathogen Fusarium culmorum. *FEMS Microbiol Ecol* **94**(6),10.1093 (2018).
115	Koraiol	Hoogendoorn, K., Barra, L., Waalwijk, C., *et al*. Evolution and Diversity of Biosynthetic Gene Clusters in Fusarium. *Frontiers in microbiology* **9**, 1158 (2018).
116	alpha-gurjunene	Schmidt, C. O , Bouwmeester, *et al*. Isolation, Characterization, and Mechanistic Studies of (-)-α-Gurjunene Synthase fromSolidago canadensis. *Archives of Biochemistry and Biophysics* **364**(2), 167-177(1999).
117	Cycloaromadendrene	María Jesús Durán-Pea, Ares J , Hanson J R , *et al*. Biological activity of natural sesquiterpenoids containing a gem-dimethylcyclopropane unit. *Natural Product Reports* **32**(8),1236-1248 (2015).
118	Cycloseychellene	Akhila A , Sharma P K , Thakur R S . Biosynthetic relationships of patchouli alcohol, seychellene and cycloseychellene in Pogostemon cablin. *Phytochemistry* **27**(7),2105-2108 (1988).
119	Ishwarene	Ghomi J S, Masoomi R, Kashi F J, *et al*. In vitro bioactivity of essential oils and methanol extracts of Salvia reuterana from Iran. *Natural Product Communications* **7**(5), 1934578X1200700527 (2012).
120	Longicyclene	Köpke D, Schröder R, Fischer H M, *et al*. Does egg deposition by herbivorous pine sawflies affect transcription of sesquiterpene synthases in pine?. *Planta* **228**(3), 427-438 (2008).
121	cyclomytaylene	Harinantenaina L, Asakawa Y. Chemical constituents of Malagasy liverworts. 6. A myltaylane caffeate with nitric oxide inhibitory activity from Bazzania nitida. *Journal of natural products* **70**(5), 856-858 (2007).
122	Anastreptene	Ng S Y, Kamada T, Suleiman M, *et al*. A new cembrane-type diterpenoid from Bornean liverwort Chandonanthus hirtellus . *Journal of Asian natural products research* **18**(7), 690-696 (2016).

Cgl06493-COP sequence
ATGTCTTCAGATGATCAATACAGAGTTTCATGCAAGAGAACTTCTACAGATATGGATCC
ATCTTCAAACGATGTTGCACCAACAAAGAGAATCATGTTGGGTAGAAGACCATCTATTA
CTACATCTTCAGATATTTCACCATCTGAATCTTCATCTCCACCAGCTACTCCAGTTTCAG
AATACCAAGCTACTGCAACACAAACTTCACCATCTGATCATGGTTCTGCTGCATACGAT
AGAAATTTGAGAGATATCATGGCTGCAGATGCTAAGTACGTTAGAATCCCAGATTGTTT
TTCATCTATCATGTCAGTTGAACCAGCAATGAACGTTAACTGGGAAAGATTGAAGGAA
GAAGCTAACGCATGGATCAAGGATATCTATCATTTGTCAGATGCTCAAGCTAAGAAACA
TTCTAGAGCTAACTTCGCTTTTATGAACGCTATGTGGATTCCATACGCAGATGAAGAATC
TTTTAGAGTTATGTTGGATTGGAACAACTGGGTTTTCGCTTTCGATGATCAATTCGATGA
AGGTCATTTGAAAGATGATCCAGTTAAAGCACAAAAAGAATTGGATGCTCATATGGCAA
TTTTAGAAGATACAAATCCACCAGTTCAAAGAGATGATAACCCAATCCATTACGTTTTC
CAAACTACATGGGATAGATTCAAGAAAAGAACTTCACCAGAATTGCAAGCTAGATATA
GAGCATCTATGAAAGGTTACTTTGAAGGTTTGATTGGTCAAGTTAAGGTTCAAGAATCA
CAAAAAGCATTGAAGATCTCTGTTAAGCAATACATGGATTTCAGAAGAGCTACAATTGC
ATGTGAACCATGTTATGCTTTAGTTGAATACGCACATGGTATCTCAATCTCTCAAGAACA
AGTTGATCATGAATCAGTTCAAACATGTATGCAAACTGCTTCTGATTTGGTTATTTTGGT
TAACGATATCTTGTCATACAGAAAAGATTTGGAACAAGGTGTTGATCATAATTTGATTTC
ATTGTTGAAAGCTCAAGGTTACTCTACTCAAGCTGCAGTTGATAAGATCGGTGACATGA
TCGATGAATGTTACAAGAGATGGTACGGTGCAATGTCTAGAATGCCATTATGGGGTGAA
AAGATTGATAGAGAAGTTTTGAGATATTTGGATGGTTGTAGAAACATCGCTTTGGGTAA
TTTGCATTGGTCATATGAATCTGGTAGATACTTGGGTGCTGAAGGTGCAGAAGTTAGAC
AAACAAGAATCATGAGATTGCCATCTCCAGGTGTTTGA

Supplemental References

1. Zhang, Y. *et al.* A gRNA-tRNA array for CRISPR-Cas9 based rapid multiplexed genome editing in Saccharomyces cerevisiae. *Nature communications* **10**, 1053, doi:10.1038/s41467-019-09005-3 (2019).
2. Siemon, T. *et al.* Semisynthesis of Plant-Derived Englerin A Enabled by Microbe Engineering of Guaia-6,10(14)-diene as Building Block. *Journal of the American Chemical Society* **142**, 2760-2765, doi:10.1021/jacs.9b12940 (2020).
